# STORM imaging reveals the spatial arrangement of transition zone components and IFT particles at the ciliary base in Tetrahymena

**DOI:** 10.1038/s41598-021-86909-5

**Published:** 2021-04-12

**Authors:** Khodor S. Hazime, Zhu Zhou, Ewa Joachimiak, Natalia A. Bulgakova, Dorota Wloga, Jarema J. Malicki

**Affiliations:** 1 Bateson Centre and the Department of Biomedical Science, University of Sheffield, Western Bank, Sheffield, S10 2TN UK; 2 Nencki Institute of Experimental Biology Polish Academy of Sciences - Laboratory of Cytoskeleton and Cilia Biology, 3 Pasteur Street, 02-093 Warsaw, Poland

**Keywords:** Computational biology and bioinformatics, Genetics, Mutation, Sequencing, Developmental biology, Ciliogenesis, Biological techniques, Biological models, Imaging, Microscopy, Sequencing, Software

## Abstract

The base of the cilium comprising the transition zone (TZ) and transition fibers (TF) acts as a selecting gate to regulate the intraflagellar transport (IFT)-dependent trafficking of proteins to and from cilia. Before entering the ciliary compartment, IFT complexes and transported cargoes accumulate at or near the base of the cilium. The spatial organization of IFT proteins at the cilia base is key for understanding cilia formation and function. Using stochastic optical reconstruction microscopy (STORM) and computational averaging, we show that seven TZ, nine IFT, three Bardet–Biedl syndrome (BBS), and one centrosomal protein, form 9-clustered rings at the cilium base of a ciliate *Tetrahymena thermophila*. In the axial dimension, analyzed TZ proteins localize to a narrow region of about 30 nm while IFT proteins dock approximately 80 nm proximal to TZ. Moreover, the IFT-A subcomplex is positioned peripheral to the IFT-B subcomplex and the investigated BBS proteins localize near the ciliary membrane. The positioning of the HA-tagged N- and C-termini of the selected proteins enabled the prediction of the spatial orientation of protein particles and likely cargo interaction sites. Based on the obtained data, we built a comprehensive 3D-model showing the arrangement of the investigated ciliary proteins.

## Introduction

Cilia are microtubule-based, finger-like cell protrusions which can be roughly divided into two categories: the motile cilia whose primary function is cell motility or translocation of materials along the surface of the ciliated epithelial cell^1^ and the sensory cilia which house a variety of signal transduction pathways and thus are essential in cell-environment communication and cell response to stimuli^2–4^. Cilia emerge from basal bodies, centriole-homologous structures that anchor and position cilia within the cell surface^5^. The region where the outermost microtubules of the basal body triplets are terminated, marks the most proximal part of the cilium, called the transition zone (TZ). The TZ has a unique ultrastructural organization and protein composition. The characteristic feature of TZ is Y-links, the Y-shaped structures that connect microtubule doublets of the axoneme to the ciliary membrane^6^. The biochemical and genetic analyses of the TZ revealed a network of interactions between TZ proteins and led to the classification of the TZ proteins into two main modules: “MKS/MKSR” and “NPHP”^7–9^.

The TZ together with the transition fibers (TF) that attach basal body to the ciliary membrane, act as a “selecting gate” regulating intraflagellar transport (IFT)-dependent trafficking of proteins to and from cilia (anterograde and retrograde transport, respectively) and thus controlling ciliary protein composition^7–10^. The main mechanism that transports proteins inside cilia is IFT, mediated by large protein complexes, known as IFT particles. The transport of the IFT particles along the microtubules within the ciliary shaft is powered by heterotrimeric kinesin-II (anterograde) and cytoplasmic dynein-1b/2 (retrograde) motors^11^. The IFT particles are composed of two main subcomplexes, IFT-A and IFT-B (subdivided into IFT-B1 and IFT-B2)^12,13^. The IFT-B subcomplex is essential for anterograde transport and cilia assembly^14,15^ whereas the IFT-A subcomplex plays a role in the retrograde transport^16–18^ and in the anterograde transport of some membrane proteins^19,20^. A complex of Bardet–Biedl Syndrome (BBS) proteins, the so-called BBSome, mediates IFT interactions with transmembrane cargo, and also associates with the IFT particle^21,22^. These octameric complexes function as adaptors mediating mainly the removal of some ciliary transmembrane and peripheral membrane proteins^12,13^.

IFT particles carry cargo protein complexes, such as dynein arms^23^ and the most abundant ciliary component, tubulin^24^. The assembly of IFT particles and trains takes place at the cilia base^25,26^. Transmission electron microscopy (TEM) immunogold localization studies have revealed that in *Chlamydomonas*^25^ and mice photoreceptor cells^27^, IFT52, IFT57, IFT88, and IFT140 concentrate at the distal end of the basal body (BB) and the TFs. Moreover, IFT particles assemble into even larger arrays, known as IFT trains^28^. TEM and cryoelectron-tomographic analyses of the IFT particles in *Chlamydomonas* flagella revealed two types of IFT trains, short and long, with approximate lengths of 250 nm and 700 nm, respectively^28^. Similar heterogeneity of the IFT trains was observed in *Trypanosoma* flagella^29^. Recently, it was also shown that in primary cilia assembled by MDCKII cells the estimated length of the IFT trains is approximately 900 nm^30^.

Recent emergence of several super-resolution imaging tools that are suitable to pinpoint subtle differences in the arrangement and distribution of proteins enabled the detailed analyses of some of the TZ, IFT and TF proteins^31–35^. However, it is still unclear where exactly the IFT and BBS proteins assemble to form particles and dock before they gain access to the cilium. Answering this is essential for understanding how IFT/BBS is regulated at the cilia base to control cilia formation and function and how it is affected by disease-related mutations.

Here we studied the spatial organization of TZ, IFT and BBS proteins at the cilia base of a ciliate *Tetrahymena thermophila.* This unicellular eukaryote assembles several hundred cilia^36,37^ which can be visualized in lateral (side) and top views. Such a high number of identical structures within a single cell facilitates averaging of many super-resolution images to increase localization accuracy and enables multi-dimensional reconstruction.

Using stochastic optical reconstruction microscopy (STORM) and computational averaging, we estimated the radial and axial positions of over 30 epitope tags fused with seven components of the ciliary TZ, nine components of the IFT particle, three components of the BBSome, and an ortholog of OFD1, the centrosomal/basal body protein that localizes to the transition fibers (distal appendages). Our analysis revealed that most of the investigated proteins are organized in ninefold clustered rings with different radii at the cilia base. We show the relative arrangement of major IFT particle subcomplexes: IFT-A, IFT-B and BBS, and localization of the IFT motor protein, kinesin II, as well as tubulin cargo binding sites. We estimated that the IFT particles reside approximately 80 nm proximal to the MKS module of the TZ. Moreover, IFT-A proteins localize peripheral to IFT-B proteins in the radial dimension, while BBS proteins localize close to the ciliary membrane. Furthermore, we show that non-lethal IFT mutations causing human ciliopathies likely affect either stability or localization of the mutated protein. Such alterations can trigger architectural changes in IFT complexes and the impairment of ciliary transport. Taking together, we generated a comprehensive 3D-model, at 15–30 nm resolution, of the organization of several TZ, IFT and BBS proteins at the *Tetrahymena* cilia base.

## Results

### Spatial organization of *Tetrahymena* TZ proteins

**Figure 1:**
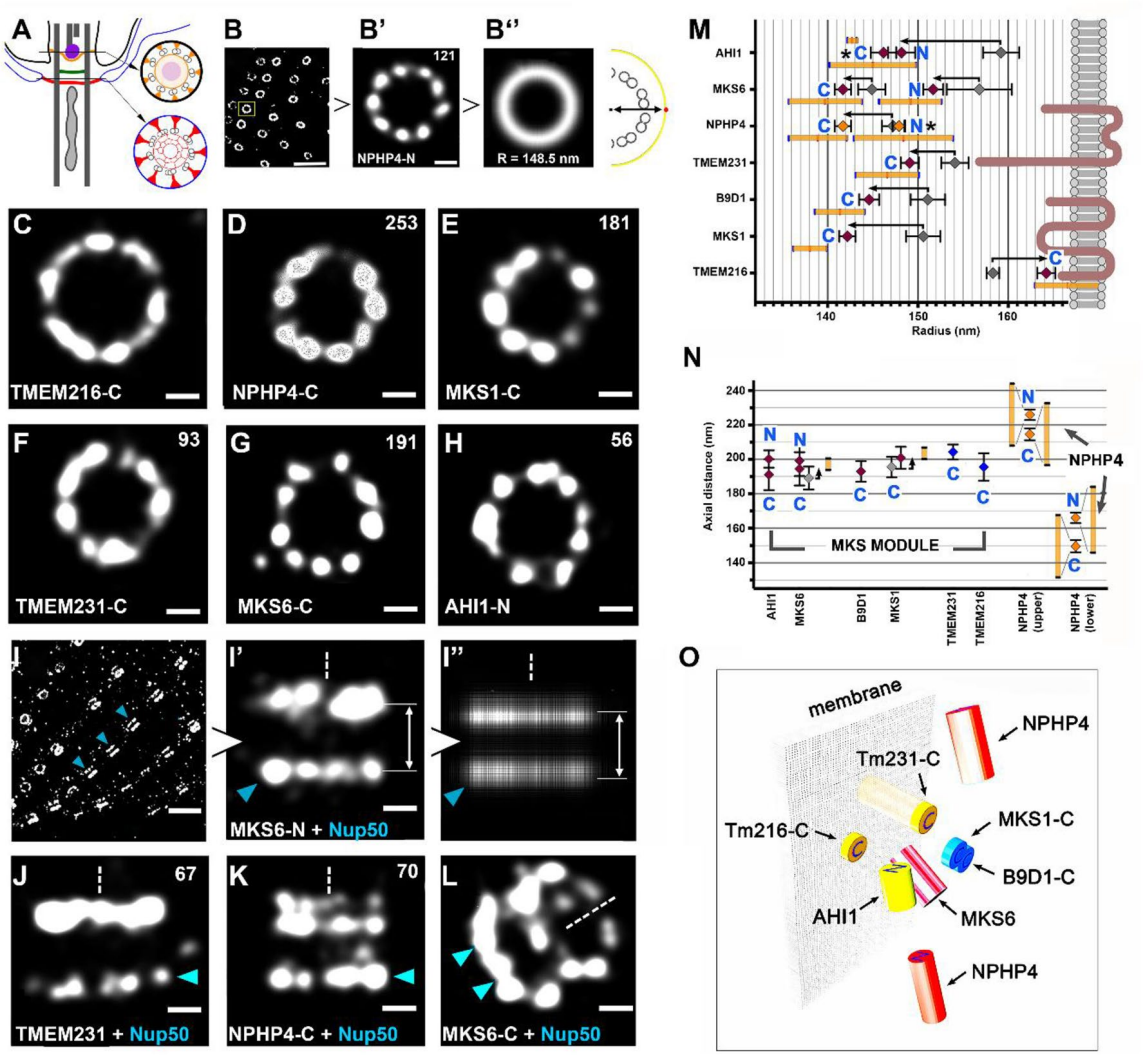
Localization of the transition zone proteins. (**A**) The TZ of *Tetrahymena thermophila*. The TZ in Tetrahymena comprises three plates: distal, intermediate, and terminal. The distal plate (in orange) is positioned just below an axosome (in violet), the electron-dense area anchoring one of two central microtubules. The cross-section at the distal plate shows sites known as a ciliary necklace, nine clusters of the electron-dense material connecting the outer doublets with the ciliary membrane. The terminal plate (in red) is the most proximal plate of the TZ and marks the border between basal body and cilium. At the level of the terminal plate, the outer doublet microtubules are connected with the membrane of the alveoli (flattened cisternae that underlie plasma membrane, here in blue) by nine clusters of the electron-dense material resembling inverted triangles. The central part of the terminal plate has a characteristic “lace-like” structure (drawing was prepared based on the TEM images from^90,91^). Please see also TEM localization studies of TZ proteins in Paramecium summarized in **G**^76^. (**B**–**B**′′) Overview of image collection and processing method for top view images. (**B**) Low magnification view of the surface of a *Tetrahymena* cell expressing a tagged TZ component. (**B**′) Higher magnification of a single cilium base at the TZ level in cells expressing N-terminally tagged NPHP4. (**B**′′) Image of tagged NPHP4 averaged from 121 cilia bases. To the right is a schematic representation of the radial position (R). (**C–H**) STORM images of the top view of six TZ fusion proteins (as indicated) with either N- or C-terminally attached 3HA tag. (**I–I**′′) Overview of image collection and processing method for side view images. NUP50 was used as a stipulated reference marker and indicated by a blue arrowhead. (**J,K**) Examples of side views for two proteins as indicated. NUP50 positive ring indicated by a blue arrowhead. (**L**) Oblique view of MKS6 with a C-terminal 3HA tag. Dashed line indicates cilium axis. A NUP50-positive ring indicated by blue arrowheads. (**M**) Radial positions of TZ proteins localized using direct (indicated in colored diamonds) and indirect (indicated in grey diamonds) staining. Black arrows indicate differences between the two localization methods and show the shift between the two measurements. Asterisks indicate no statistically significant difference between direct and indirect measurements. (**N**) Axial positions of TZ proteins relative to NUP50. Note that MKS and NPHP4, a component of the NPHP module, proteins localize differently. Yellow bars in (**M,N**) show the uncertainty of epitope locations. (**O**) A 3D model of analyzed TZ proteins. Scale bars in (**B,I**), 1 µm; in (**B**′,**C–H**,**J–L**), 100 nm. In (**B**′**,C–H,J,K**), the numbers of rings (or side views) used to calculate the average radius (or axial distance**)** of each protein are shown (top right). The rings/side views were collected from at least 10 different cells from 3 independent experiments.

TZ proteins are well conserved in evolution (Supplementary Table S1, Supplementary Fig. S1). Thus, the analysis conducted in simple model organisms such as a ciliate *Tetrahymena thermophila*, can contribute to the general knowledge and understanding of ciliary proteins’ localization and function. Therefore, we aimed to characterise and reveal the nanoscale organization of the TZ proteins at the cilia base in *Tetrahymena thermophila* (Fig. 1A). For this purpose, we used STORM microscopy to reveal the localization of selected *Tetrahymena* TZ proteins and to compare their spatial organization within the TZ region with the arrangement of TZ proteins described in mammalian motile and primary cilia.

Using a bioinformatics approach, we identified *Tetrahymena* orthologs of the TZ proteins (Supplementary Fig. S1) and engineered *Tetrahymena* cells expressing selected proteins as C-terminally 3HA-tagged fusions under the control of the respective native promoters (Supplementary Fig. S2). Those were: two presumptive transmembrane proteins, TMEM216/MKS2 and TMEM231/JBTS20, two B9 domain-containing proteins, B9D1 and MKS1, a coiled-coil domain-containing protein, MKS6/CC2D2A, a WD repeat domain-containing protein AHI1/JBTS3 and NPHP4, a protein reported to have C2 domains^38^ and ASH/MSP domains^39^. Additionally, we engineered cells expressing AHI1, MKS6, and NPHP4 proteins with 3HA tag attached to their N-terminal end under the control of an exogenous promoter (Supplementary Fig. S2). Cells expressing HA-tagged TZ proteins were stained either directly with anti-HA antibodies conjugated with fluorophore or indirectly using anti-HA antibodies followed by fluorophore-conjugated secondary antibodies.

**Figure 2:**
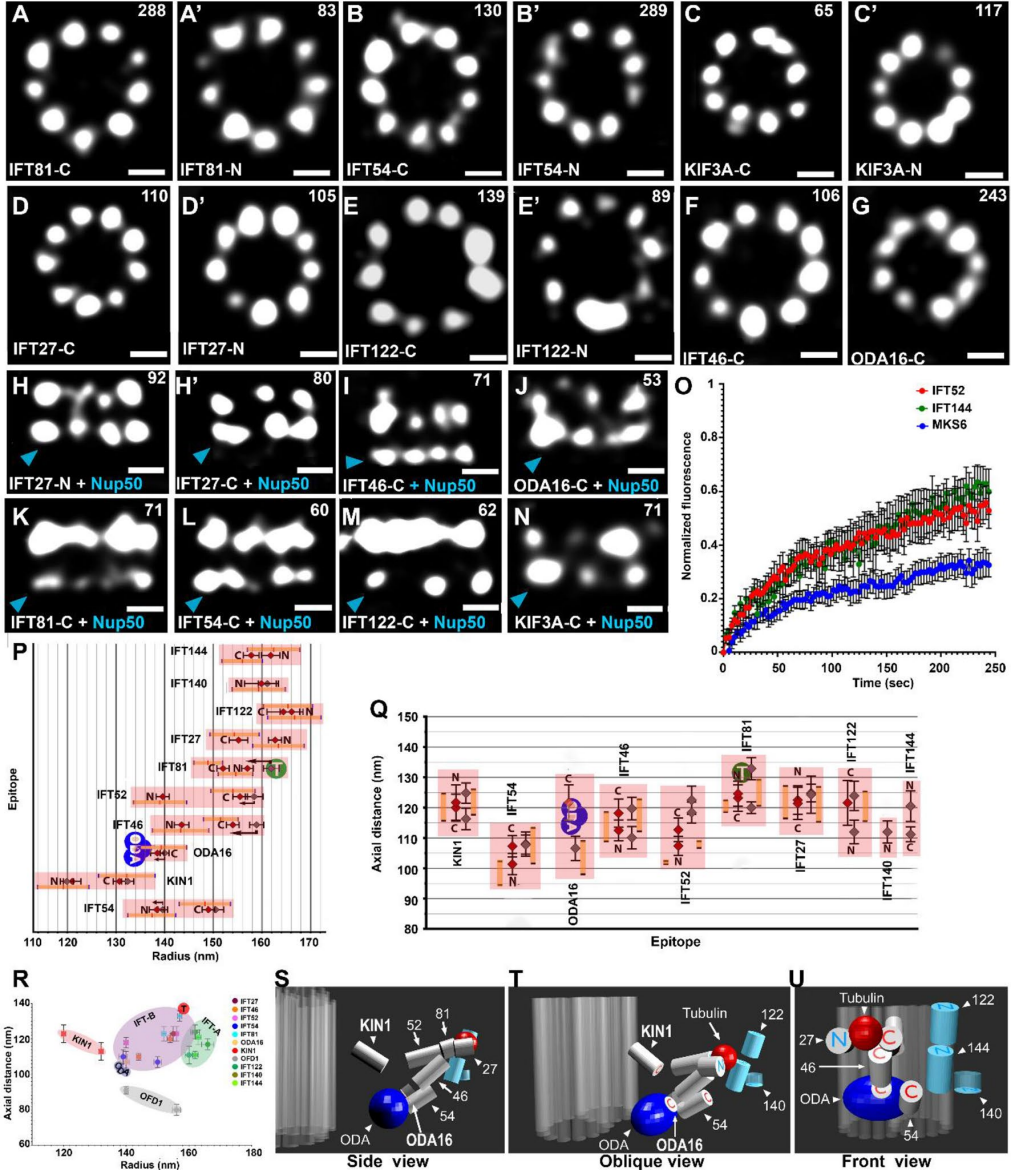
Localization of IFT particle proteins. (**A–N**) STORM images of top and side (lateral) views of N- or C-terminally 3HA tagged IFT proteins, KIN1/KIF3A kinesin and an adaptor protein ODA16 as indicated. (**O**) FRAP analysis of two fluorescent IFT proteins: IFT52 and IFT144 compared to MKS6, a TZ component. (**P**) Radial positions of IFT proteins localized using direct (indicated in color) and indirect (in grey) staining. Arrows indicate differences between the two localization methods. (**Q**) Axial positions of IFT proteins relative to NUP50 (indicated by a blue arrowhead). In (**P,Q**) Predicted positions of tubulin (T) and outer dynein arms (ODA) cargo are indicated. Yellow bars in (**P,Q**) show the uncertainty of epitope locations. (**R**) Graph showing radial versus axial positions of all investigated IFT proteins. Means ± SEM are shown for each protein. (**S–U**) 3D model of IFT proteins’ localization. Side, oblique, and front views are provided. Scale bars in (**A–N**), 100 nm. In panels (**A–N**), the numbers of rings (or side views) used to calculate the average radius (or axial distance) of each protein are indicated (top right). The rings/side views were collected from at least 10 different cells in at least three independent experiments.

The confocal imaging of *Tetrahymena* cells double-labelled with anti-HA and either anti-tubulin or anti-centrin antibodies showed that selected TZ proteins localized at the ciliary base distal to the basal body (Supplementary Fig. S2B–C′). When HA-tagged TZ proteins were visualized using STORM, they appeared mostly as 9-cluster rings (Fig. 1B,B′,C–H, Supplementary Fig. S3A–C). The radii of these rings corresponded to a distance between the antigen (tag fused to the protein) and the cilium center (Fig. 2A′′). The measurements based on indirect versus direct detection of the analyzed epitopes differed by 1–11 nm (Fig. 1M). This agrees with the antibody size of 12 nm^40^ and provides a method to evaluate the size and direction of a linkage error introduced by antibody labelling (see “Materials and methods” section, Fig. 1M).

TMEM216 and TMEM231, are tetraspan and two-pass transmembrane proteins, respectively. The C-terminal tail of human TMEM216, exposed to the ciliary lumen, is three amino acids long^41^. Therefore, a fluorescent signal of the 3HA tag attached to its C-terminus is a good marker to estimate the distance between the cilium center and the ciliary membrane. The tail of *Tetrahymena* TMEM216 is predicted to be composed of 12 amino acid residues (http://www.cbs.dtu.dk/services/TMHMM/). STORM imaging revealed that *Tetrahymena* TMEM216 formed the largest ring among all investigated TZ proteins, with a radius of 164 ± 5 nm (Fig. 1C,M). The 30 amino acid C-terminus of *Tetrahymena* TMEM231 localized at a radius of 149 ± 6 nm, approximately 15 nm away from TMEM216-C terminus (Fig. 1F,M).

The investigated luminal TZ components formed rings of smaller diameters. The C-terminal ends of two B9 domain proteins, MKS1 (Fig. 1E) and B9D1 (Supplementary Fig. S3A), localized close to each other, with radii of 143 ± 6 and 144 ± 5 nm, respectively (Fig. 1M). An examination of the N- and C-terminal epitopes of the remaining three investigated TZ proteins, MKS6 (Fig. 1G), NPHP4 (Fig. 1B,B′,D), and AHI1 (Fig. 1H, Supplementary Fig. S3B), revealed that the N-termini of MKS6 and AHI1 localized closer to the cilium membrane compared to their respective C-terminal ends (Fig. 1M), whereas in the case of NPHP4, the radii of both C- and N-termini localized about 20 nm from the ciliary membrane (Fig. 1M). Notably, in the radial dimension, the N-terminus of MKS6 was only ~ 2 nm apart from the C-terminus of TMEM231 (Fig. 1M). These data suggest that TMEM231 could participate in the anchoring of MKS6 and the B9 protein complex (MKS1/B9D1/B9D2) to the membrane.

Next, we sought to localize the TZ proteins along the cilium axis (side views). In *Tetrahymena* cells, an anti-NUP50 antibody detected protein(s) that localized proximal to the TZ proteins (Supplementary Fig. S3D–D′′). In STORM, the signal from anti-NUP50 antibody formed consistent ninefold symmetrical rings at the cilia base (Supplementary Fig. S3E). We used the signal generated by anti-NUP50 antibody as an independent stipulated reference to estimate the axial position of the investigated TZ proteins. The transgenic *Tetrahymena* cells were double-labelled with anti-HA and anti-NUP50 antibodies and imaged using STORM (Fig. 1I–L, Supplementary Fig. S3F–M). Multiple images were collected and computationally averaged (see “Materials and methods” section, Fig. 1I–I′′). The distance between TZ proteins and NUP50 antibody signal varied between 180 and 210 nm. Thus, the analyzed TZ proteins localized to a narrow region of approximately 30 nm along the cilium axis (Fig. 1N). In contrast to other analyzed TZ proteins, NPHP4 was detected in two locations (Supplementary Fig. S3L–M): distal and proximal to other TZ components (Fig. 1K,N). In summary, the analysis of the average radial and axial positions of the proteins enabled us to generate a comprehensive super-resolution model of selected *Tetrahymena* TZ components at the cilia base (Fig. 1O).

### STORM imaging reveals IFT particles docked at the ciliary base

Before travelling to cilia, the IFT proteins concentrate at the base of the cilium. This area acts as a “selecting gate” that regulates IFT-dependent trafficking of proteins to and from cilia. To visualize the IFT particles and estimate their docking sites before entering cilia, we engineered *Tetrahymena* cells expressing either N- or C-terminally 3HA-tagged IFT proteins, five belonging to the complex B (IFT27, IFT46, IFT52, IFT54 and IFT81) and three to the complex A (IFT122, IFT140 and IFT144). Moreover, we analyzed the localization of ODA16, an IFT cargo adapter protein^42,43^, and KIN1, the *Tetrahymena* ortholog of KIF3a^44^, the major anterograde transport motor^45,46^. The C-terminally tagged proteins were expressed under the control of the respective native promoter while the N-terminally tagged proteins were under the control of an exogenous promoter.

Confocal imaging revealed that in full-length cilia the IFT proteins were mainly detected at the ciliary base/basal body and cilia tip (Supplementary Fig. S4A–F′) in agreement with earlier localization studies of IFT52, IFT57, IFT80, IFT122 and IFT172^47–50^. The IFT signal along the ciliary length was undetectable, likely due to very low concentration of the IFT particles in the full-length cilia^51^. STORM imaging of the cells expressing IFT fusion proteins showed that at the cilia base the IFT subunits localized at nine distinct sites, which we will further refer to as “docking sites” (Fig. 2A–G, Supplementary Fig. S5A–D). These observations imply that prior to their entry into the ciliary shaft, the IFT proteins dock with high affinity at nine sites at the cilia base^52^.

*Tetrahymena* cilia can be experimentally removed (deciliation) and synchronously regenerated^53^. The full-length, approximately 6 µm long cilia are formed within about 2 h after deciliation. To examine the localization of IFT proteins in growing cilia *Tetrahymena* cells were deciliated and after 30 min cells were fixed and immunostained. TIRF imaging revealed prominent IFT signal in the entire ciliary shaft of regenerating cilia (Supplementary Fig. S4G,G′) in agreement with previous reports^50^. STORM imaging of these regenerating cilia revealed that although some IFT proteins were still present at their docking sites, the vast majority of the IFT proteins translocated from cilia base into the ciliary shaft (Supplementary Fig. S4H–I′).

To further investigate the dynamics of the IFT resting at their docking sites, we expressed IFT52-GFP, a component of the IFT-B complex, and IFT144-GFP, a component of the IFT-A complex, both under the control of their respective native promoters. For comparison, we also expressed the TZ component GFP-MKS6. Fluorescence Recovery After Photobleaching (FRAP) analysis revealed that IFT proteins anchored at their docking sites in cells with full-length cilia, were, as expected, more dynamic, compared to the structural TZ component (Fig. 2O, Supplementary Fig. S4J–J′′). These observations are in agreement with transient resting of the IFT particles before entering into the ciliary shaft^52^. The observed recovery of the IFT proteins at the docking sites can occur either by proteins entering cilia for the anterograde transport or leaving the cilium through the retrograde transport.

Having established the status of IFT proteins at the cilia base, next we analyzed the localization of the IFT proteins in their docking sites (Fig. 2A–G, Supplementary Fig. S5A–D). The computational averaging of all rings collected per IFT protein allowed to estimate the radial position of different IFTs (Fig. 2P). The investigated IFT-B proteins (Fig. 2A–B′,D,D′,F, Supplementary Fig.S5A,B) spanned a radial distance of 139–162 nm, where 162 ± 6 nm corresponds to the largest radius of IFT81 N-terminus (3HA-IFT81) (Fig. 2A′), whereas the smallest radius of 139 ± 7 nm corresponds to the IFT54 N-terminus (3HA-IFT54) (Fig. 2B′). In contrast, the investigated IFT-A proteins occupied a narrower space of 160–166 nm from the center (Fig. 2E,E′, Supplementary Fig. S5C,D) and did not overlap radially with most IFT-B proteins (Fig. 2P–R). The N-terminus of IFT122-N (3HA-IFT122) displayed the largest radius (166 ± 9 nm) (Fig. 2E′), whereas the N-terminus of IFT140 (3HA-IFT140) the smallest radius (160 ± 8 nm) (Supplementary Fig. S5D). Thus, IFT-A complex proteins are located peripheral to IFT-B complex in the radial dimension (Fig. 2P–R). This agrees with previous finding that IFT-A localizes between IFT-B and the ciliary membrane^52,54^ and supports the idea that IFT-A proteins are involved in the transport of membrane proteins^19,55^.

Analysis of the lateral localization of IFT proteins revealed that A and B sub-complexes partly overlap along cilium axis and span a combined axial distance of approximately 30 nm at the cilia base (Fig. 2H–N,Q,R, Supplementary Fig. S5E–L′). To extend this analysis, we localized kinesin KIN1, the motor protein that powers the anterograde IFT. Like IFT proteins, KIN1 accumulated in 9 sites around the cilium base (Fig. 2C,C′) and overlapped with IFT-B proteins in the lateral dimension (Fig. 2N,Q,R). Our data show that the N-terminus of KIN1 (motor domain) localized very close to the expected location of the peripheral microtubules, whilst its C-terminus (cargo-interacting domain) localized near IFT proteins (Fig. 2P), which is consistent with KIN1’s role in binding and transporting the IFT particle in a microtubule-dependent manner. In rotary shadowing electron-micrographs, murine Kif3a/b kinesin is 50 nm long^56^. Our studies reveal that in its *Tetrahymena* ortholog, KIN1, the distance between the N- and C-termini is much shorter, suggesting that KIN1 might assume a more compact conformation at the docking site.

The IFT-cargo interactions are frequently mediated by the ciliary proteins that are not constitutively associated with the IFT particle. The interaction of the IFT particle with the preassembled outer dynein arms (ODA) is mediated by ODA16, an adaptor protein that binds IFT46^43^ and provides a binding interface for outer dynein arm cargo via its C-terminal β-propeller domain^42,43^. We found that IFT46 N-terminus and ODA16 C-terminus localized more centrally than the investigated IFT components (Fig. 2G,J,P,Q), implying that outer dynein arms are transported in the vicinity of microtubules.

The IFT subunits can also directly interact with cargo proteins. Tubulin, the most abundant cilia component, is transported by binding the N-termini of IFT74 and IFT81^57^. Our analysis suggested that the IFT81 N-terminus (Fig. 2A′,P), and consequently the tubulin binding site, localized to the outer, membrane-directed surface of the investigated IFT proteins (Figs. 2P, 4). On the other hand, the N-terminus of IFT54 has been also postulated to play a role in the tubulin binding^58^. If so, it would provide a binding site, which is independent of IFT74/81 as it is located close to microtubule doublets (Fig. 2P).

**Figure 3:**
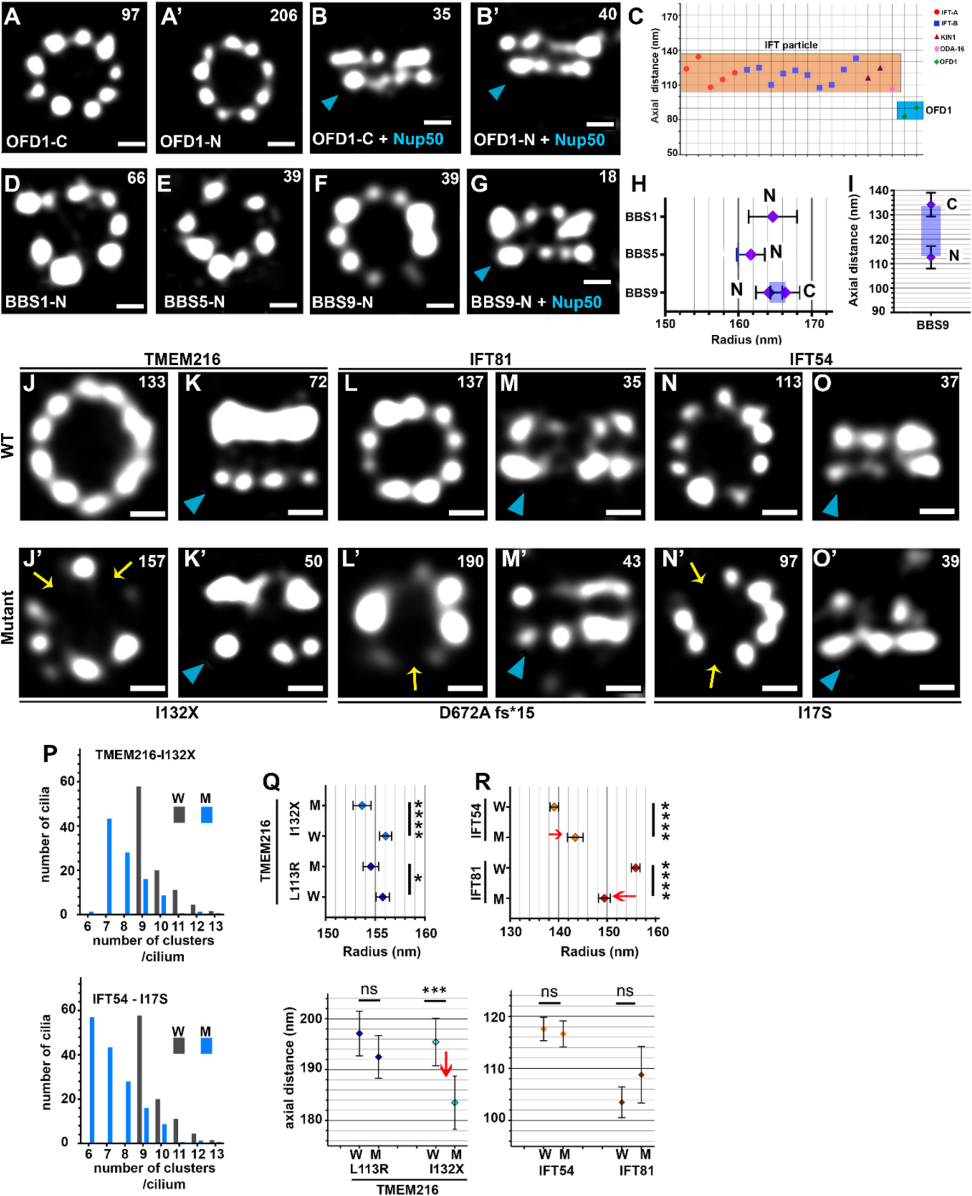
Localization of BBSome, OFD1, and selected TZ and IFT proteins carrying mutations causing ciliopathies in humans. (**A,A**′) STORM images of the top views of the centriole/basal body protein, OFD1, tagged at the C- and N-termini. (**B,B**′) Side views of OFD1 relative to NUP50 (indicated by a blue arrowhead). (**C**) Axial position of OFD-1 relative to the IFT particle. (**D–G**) STORM images of top (**D–F**) and side views relative to NUP50 (indicated by a blue arrowhead) (**G**) of 3HA-tagged BBS proteins as indicated. (**H**) Radial positions of BBS proteins localized using direct staining. (**I**) Axial positions of BBS9 C- and N-termini. (**J–O**′) STORM images of top (**J–J**′**,L–L**′**,N–N**′) and side views relative to NUP50 (indicated by a blue arrowhead) (**K–K**′**,M–M**′**,O–O**′) of IFT and TZ mutant proteins as indicated. Yellow arrows in (**J**′,**L**′,**N**′) indicate missing clusters that are visible in (**J,L,N**). (**P**) Histogram of TMEM216 (top) and IFT54 (bottom) showing the difference in number of clusters in rings between wild-type ((**W**), grey) and mutant (**M**), blue) proteins. (**Q–R**) Radial (top) and axial (bottom) positions of IFT and TZ wild-type (W) and mutant (M) proteins as indicated. Red arrows indicate differences in measurement of the radial or axial positions between wild-type and mutant proteins as indicated. Scale bars in (**A–B**′**,D–H,J–O**′), 100 nm. In panels (**A–B**′,**D–G**,**J–O**′), the numbers of rings (or side views) used to calculate the average radius (or axial distance) of each protein are indicated (top right). The rings/side views were collected from at least 10 different cells from at least 3 independent experiments.

**Figure 4:**
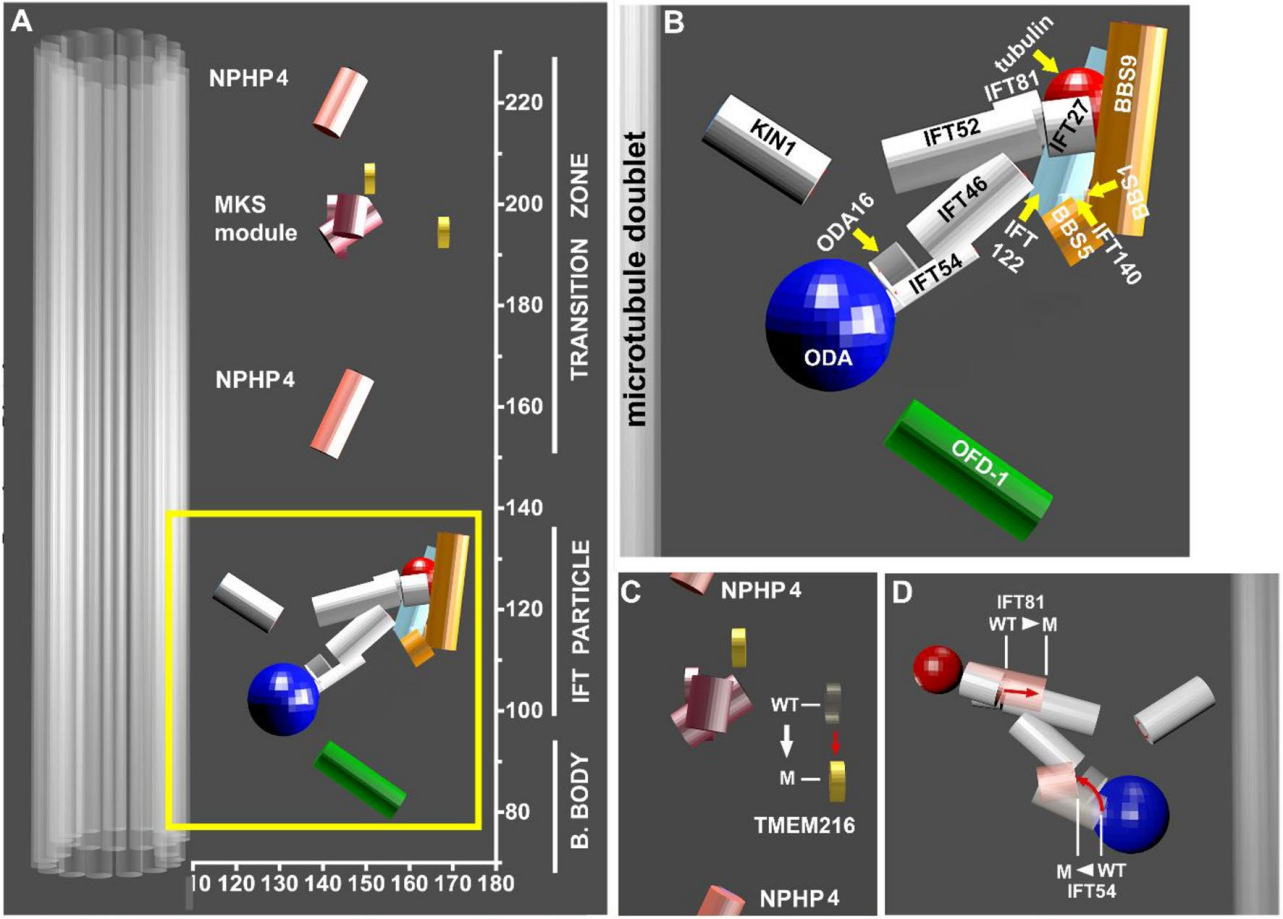
Model of the spatial arrangement of the TZ, IFT and BBS proteins at the cilia base of *Tetrahymena thermophila*. (**A**) A 3D model constructed based on STORM data. The arrangement of proteins in circumferential dimension takes into account published protein–protein interactions. (**B**) Inset in (**A**) showing the position of the BBS proteins with respect to the IFT particle with indicated proteins. (**C,D**) A 3D model showing the shift of radial and axial positions in TZ and IFT mutant proteins.

As an additional reference, we also localized OFD1, a centrosomal/basal body protein known to localize to the distal ends of the centrioles^59,60^. In top views, both C- and N-terminus of OFD1 localized in ninefold symmetrical rings at the cilia base (Fig. 3A,A′). In the axial dimension, OFD1 localized proximal to the IFT particle (Figs. 3B,C, 4A, green cylinders) suggesting that IFT particle is located between the distal end of basal body and the TZ. The relative localization studies of IFT proteins using anti-NUP50 antibodies and tagged-OFD1 enabled us to generate a model of the IFT particle anchored in its docking site (Figs. 2S–U, 4).

### BBS proteins localize near the ciliary membrane at the cilia base

The transport of the ciliary transmembrane proteins relies on the BBSome^61^, a complex closely associated with IFT transport^22,62,63^. IFT27, a component of the IFT-B subcomplex, serves as an anchorage point onto which the BBSome is connected to the IFT particle^10,21,64,65^.

To shed light on the spatial relationship between IFT and BBS complexes at the ciliary base, we engineered *Tetrahymena* cells expressing BBSome core proteins as fusions with 3HA tag attached to either N-terminus of BBS1 and BBS5, or N- and C-terminus of BBS9. STORM imaging revealed that radially and axially, these epitopes localized in the vicinity of IFT complex A proteins, at the external face of the IFT particle (Fig. 3D–I) which is consistent with their role in the interactions with transmembrane proteins^62^. We also found that the pattern of BBS proteins at the cilia base was not as regular as that of IFT proteins and more difficult to visualize. Presumably, it was due to less stable association of the BBSome with the IFT particle, or because the abundance of the BBSome was lower than that of the IFT particle as shown in previous quantitative proteomic studies^66^.

### Mutations causing ciliopathies in humans trigger architectural changes in the TZ and IFT proteins at the cilia base

Mutations in genes encoding TZ, IFT or BBS proteins cause syndromic ciliopathies and affect cilia function in many cell types, tissues and organs^1, 67–70^. To investigate the outcome of the pathogenic mutations in five genes: BBS5 (Ala323CysfsX57)^71^, IFT54 (I17S)^72^), IFT81 (D672Afs)^73^, TMEM216 (L114R and L133X)^74^, and TMEM231 (Ile232SerfsX)^75^ that were reported to cause ciliopathies in humans but not analyzed in details, we replaced the wild-type copies of the *Tetrahymena* genes with the allele carrying a ciliopathy-related mutation (Supplementary Fig. S2A, Supplementary Table S3). Corresponding mutations in *Tetrahymena* TMEM231 (Lys209SerfsX12) and BBS5 (Ala332Cysfs57) most likely caused protein instability as we were unable to detect these proteins using western blot or imaging methods. In contrast, mutated *Tetrahymena* TMEM216 (L113R and I132X), IFT54 (I17S) and IFT81 (D709Afs) were stable and could be visualized in *Tetrahymena* cells.

STORM analyses revealed partial loss or mis-positioning of the mutated proteins within cilia base region (Fig. 3J–R). The L133X mutation in TMEM216 was identified in individuals with Joubert syndrome^74^. This low-frequency variant of TMEM216 results in a truncation of the protein within the fourth transmembrane domain. Compared to wild-type TMEM216 (Figs. 1C,M, 3J,K) the truncated variant of *Tetrahymena* TMEM216 (I132X) still localized as rings, but the radii of the rings were smaller and the number of clusters per ring was reduced (Fig. 3J′,P, upper panel, Fig. 3Q, upper panel). Moreover, the mutated protein seems to be shifted axially closer to anti-NUP50 signal (Fig. 3K′,Q, lower panel).

The deletion of 5 nucleotides and in consequence a shift of several amino acid residues in human IFT81 (D672Afs) causes blindness and intellectual disability in affected individuals^73^. When the corresponding mutation was introduced into *Tetrahymena* gene encoding IFT81 (Fig. 3L–M′,R), the mutated 3HA tagged protein still formed rings but in the radial dimension, its C-terminus seemed to be displaced toward the cilium center by approximately 9 nm (150 nm in mutants vs. 159 for wild-type).

IFT54, a component of the IFT-B subcomplex, contains an N-terminal calponin-homology (CH) domain, an arginine-rich motif and C-terminal coiled-coil domain. Most of the ciliopathies-causing mutations were identified within the CH domain suggesting that this domain plays a role in tubulin and potentially other cargo transport^72^. It was proposed that I17S substitution in human IFT54 may affect protein stability and IFT54-cargo (tubulin) interactions^72^. The *Tetrahymena* IFT54 protein with introduced corresponding missense mutation (I17S) localized to rings that appear to have larger diameters than those seen in wild-type cells (Fig. 3N–O′,R).

In conclusion, the investigated mutations in the proteins cause changes on average protein localization. Thus, one can hypothesize that in consequence, mutations interfere with the normal function and protein entry into cilia.

## Discussion

A significant number of proteins that build the ciliary transition zone as well as IFT and BBS subunits are highly evolutionarily conserved from protists to mammals, including humans^76^. It is likely that such a high level of protein similarity correlates with similar localization and function of these proteins in evolutionarily distant organisms. The localization and interactions of selected TZ proteins were investigated using super-resolution tools and biochemical approaches in various models including mammalian cells assembling either primary^33,34,77^ or motile cilia^35,78^, in the sensory cilia of the worm *C. elegans*^32^, and recently in a ciliate, *Paramecium tetraurelia*^76^. Besides RPGRIP1L/MKS5, these studies focused mostly on different sets of proteins (Table 1). This impedes comparative analyses of the spatial arrangement of TZ proteins in evolutionarily distant species.

**Table 1:** Comparison of TZ proteins diameters in *Tetrahymena* and other organisms.

	Paramecium STED^76^	mTEC STORM^35^	mTEC STED^78^	RPE1 STED^34^	Tetrahymena STORM 3HA (This study)
Tubulin	242 ± 18	–	–	–	–
MKS1	–	–	–	335 ± 24	286 ± 12 (C)
MKS2/TMEM216	288 ± 23	–	–		328 ± 10 (C)
MKS3/TMEM67/meckelin	–	–	–	374 ± 30	–
AHI1	–		232 ± 18	–	292 ± 15 (C)
					296 ± 12 (N)
B9D1	–	312 ± 25	–	–	288 ± 10 (C)
MKS6	–	–	–	–	290 ± 16 (C)
					303 ± 11 (N)
TMEM107	326 ± 23		–	–	–
TMEM231	–	328 ± 10	–	–	298 ± 12 (C)
TMEM237	–		210 ± 22	–	–
NPHP1	–	283 ± 12	–	–	–
NPHP4	253 ± 24	–	–	–	283 ± 12 (C)
					296 ± 11 (N)
RPGRIP1L/NPHP8/MKS5	248 ± 22	271 ± 9	–	284 ± 40	–

Here, using STORM imaging and computational averaging we estimated radial and proximodistal arrangement of the selected 34 epitopes in 21 ciliary proteins, including subunits of the TZ complexes, IFT particles, and the BBSome in a free-living ciliate *Tetrahymena thermophila*.

*Tetrahymena* cells can be easily manipulated genetically which enables fast protein tagging and localization of proteins of interest even if commercial antibodies are not available^53^. The determination of the radial and axial positions of both N- and C-termini of the analyzed proteins allowed us to generate a model showing not only likely spatial position of the investigated proteins within the TZ/basal body region, but also the orientation of the proteins at the cilia base. Thus, the obtained data and the generated 3D-model of the cilia base in *Tetrahymena thermophila* extend the current knowledge of the structure of the ciliary gate and transport into a ciliary shaft.

In top views, the analyzed *Tetrahymena* TZ proteins were generally visible as rings composed of nine, most often evenly distributed clusters. The subunits of the MKS module (TMEM216, TMEM231, MKS6, AHI1, B9D1, and MKS1) formed rings with decreasing diameters, whereas in the axial dimension, the investigated TZ proteins seemed to span only about 30 nm as assessed using anti-NUP50 antibody as a reference. The tetraspan transmembrane protein, TMEM216, was localized only in another ciliate, *Paramecium tetraurelia*^76^. Unexpectedly, in *Tetrahymena*, a ring formed by TMEM216-3HA had the diameter approximately 40 nm larger compared to that of *Paramecium* TMEM216-GFP. Similarly, the estimated diameter of the tagged NPHP4 was smaller in *Paramecium* (NPHP4-GFP) than in *Tetrahymena* (NPHP4-3HA). Such differences in the diameter of the protein localization between the two ciliates is puzzling. However, when we compared data obtained in *Paramecium* with those obtained in mTEC^35^ and RPE1 cells^34^, we noticed that the diameter of the RPGRIP1L/NPHP8/MKS5 ring in *Paramecium* was also smaller than those estimated in cilia assembled by the mammalian cells (Table 1), suggesting that observed differences could be due to the visualization/detection or measuring methods.

In *Tetrahymena*, a two-pass transmembrane protein, TMEM231, had an estimated diameter approximately 30 nm smaller than that reported in mouse tracheal cilia (298 ± 12 versus 328 ± 10 nm^35^). However, in mTEC cells, TMEM231 was detected using a polyclonal antibody obtained against the fragment that is predicted to be positioned outside the membrane. In the case of *Tetrahymena*, TMEM231 was detected using a C-terminal 3HA tag located in the ciliary lumen. In *Tetrahymena*, the C-terminus of TMEM231 is likely positioned close to the N-terminal end of MKS6 (approximately 2 nm apart in radial dimension (Fig. 1M)). This agrees with biochemical studies on the role of TMEM231 in organizing the MKS complex^75^ and leads to a prediction that TMEM231 might anchor MKS6 to the membrane.

Also, consistent with TZ proteins binding studies^77,79^, the two *Tetrahymena* B9 domain proteins, MKS1 (Fig. 1E) and B9D1 (Supplementary Fig. S3A) were assessed to localize close to each other, with radii of 143 ± 6 and 144 ± 5 nm, respectively (Fig. 1M). These data suggest that these two proteins might form a complex and physically interact with each other as previously reported^80,81^. Our radial measurement of B9-domain proteins at the cilia base of *Tetrahymena* is slightly different to that obtained by^35^ (B9D1) and^34^ (MKS1). Shi et al*.*^35^ and Yang et al.^34^, utilised antibodies against the endogenous proteins, while, in our studies we relied on antibodies against a C-terminal tag, which may account for the difference. The B9 domain resembles the C2 phospholipid binding domain and was hypothesized to bind membrane lipids^81^. Our data show that in *Tetrahymena* the C-terminal end of MKS1 and B9D1 localize approximately 20 nm away from the ciliary membrane (Fig. 1M). However, we cannot exclude that the N-termini of those proteins are positioned close to the ciliary membrane and allow for their interaction at the cilia base, as was previously suggested^80,81^.

The N- and C-terminal epitopes of the remaining three investigated TZ proteins, MKS6 (Fig. 1G), NPHP4 (Fig. 1B,B′,D), and AHI1 (Fig. 1H, Supplementary Fig. S3B), revealed that the N-termini of MKS6 and AHI1 localized closer to the cilium membrane compared to their respective C-terminal ends (Fig. 1M) in agreement with previous findings^77^. In the case of NPHP4, both C- and N-terminus localized on average about 20 nm from the ciliary membrane (Fig. 1M,O) suggesting that NPHP4 might have different spatial organization in *Tetrahymena* TZ than previously observed in mouse primary cilia, where N-terminus of NPHP4 interacts with the ciliary membrane protein SSTR3, and the C-terminus is directed towards the ciliary lumen^77^.

In contrast to MKS module proteins, NPHP4, the subunit of the NPHP module, was detected in *Tetrahymena* as two 9-cluster rings, one distal and one proximal to the MKS module rings. Such a localization pattern agrees with analysis in *C. elegans*, where NPHP-4 and NPHP-1 have a dual localization, one at TZ (overlapping with other TZ proteins), and another at the transition fibres (proximal to TZ)^67^. In a ciliate *Paramecium,* the axial position of the NPHP4-GFP was visualized using either confocal microscopy or immunogold labelling^76^ that likely do not permit for resolving the two localization sites. It could be hypothesized that NPHP4 proximal to MKS could interfere with IFT protein function as previously reported^82^. The relevance of such two-layer localization of a component of the NPHP module is a fascinating question for future studies.

To our knowledge the size of the IFT trains in a ciliate *Tetrahymena* is unknown. The IFT trains could be much shorter in *Tetrahymena* compared to IFT trains described in *Chlamydomonas* and *Trypanosoma* flagella and dock to and move along all nine axonemal outer doublets to support sufficient intraciliary transport. Although a formation of short IFT trains in *Tetrahymena* would correlate with the approximately 30 nm axial positioning of the IFT proteins near the BB distal end, undoubtedly further analyses are required to support this presumption.

The data concerning the IFT docking sites are limited. The immuno-EM studies showed that in mice rod photoreceptor cells IFT57, IFT88, and IFT140 accumulate near the cilium basal body^27,68^ and in *Chlamydomonas*, the IFT52 associates with the transition fibers, especially at their distal ends^25^. The co-localization of the IFT88 with TF was also observed using dual-color STED^34^ and STORM^33^ imaging in human RPE1 cells assembling primary cilia. Its distribution was described as “broad angular without a clear nine-fold symmetric pattern” at the level of the TF^33^.

Using STORM imaging and computational averaging, we visualized *Tetrahymena* IFT proteins as nine distinct clusters at the cilium base which we called “docking sites”. In the radial dimension, the IFT particles including KIN1, the *Tetrahymena* ortholog of KIF3a, spanned 40-50 nm. IFT-B proteins occupied a space of about 20 nm, while IFT-A proteins localised within 5 nm from each other in the radial dimension. IFT-A subcomplex appeared to be positioned peripheral to IFT-B complex, as was previously reported^54^. This suggests that IFT-A proteins are involved in the transport of membrane proteins, in agreement with data showing that IFT complex A indirectly mediates ciliary entry of membrane proteins^19,55^.

The IFT particles transport cargoes to cilia. For example, tubulin, which is the most abundant component of cilia, is transported by binding to the N-termini of IFT74 and IFT81^24^. We show that IFT81-N terminus likely localizes to the external periphery of the IFT particle, implying that this is where tubulin cargo might bind. In contrast, outer dynein arms binding site appears to attach to the side of the IFT particle that is closer to the peripheral doublet microtubule side, as suggested by ODA16-C terminus localization. This reveals that IFT-cargo sites at the cilium base localize at distinct positions.

Cryo-electron microscopy studies estimate radial dimensions of IFT particle to be 25 nm^28^. Based on our STORM data, we estimate an average radial distance between KIN1 and IFT81 tubulin binding site to be more than 40 nm. It is tempting to speculate that while residing in the docking site, the IFT particle assumes less compact (i.e. open) conformation.

A transport of the ciliary transmembrane proteins relies on a complex of BBS proteins^61^. BBS genes have been identified as defective in Bardet–Biedl Syndrome^63^. Because of its medical importance and a role in cargo interactions, we localized three subunits of the BBSome: BBS1, BBS5, and BB9. These proteins localized to the same region as IFT-A complex, consistent with their role in the interactions with transmembrane proteins^62^. Both BBS1 (N-terminus) and BB9 (C- and N-termini) seem to be peripheral, supporting the previously reported association of the BBSome with the membrane^83^. IFT27, a GTPase that belongs to the IFT-B complex, was shown to anchor the BBSome to the IFT particle and is thought to mediate the exit of BBSome from cilia^21,64,65^. Consistently with these findings, our results show that IFT27 is positioned closely to BBS proteins implying their possible association. Additionally, our model suggests that IFT27 is located near the N-terminus of IFT81, which is surprising, as biochemical studies suggested that the IFT25-IFT27 dimer binds to the C-terminal end of the IFT74-IFT81 dimer^84^. This discrepancy could be either due to differences in protein behaviours in vivo and in vitro or due to the particularly complex configuration at the cilia base.

Taken together, our analysis of the cilia base in *Tetrahymena thermophila* provides several insights into the assembly and overall spatial organization of the IFT particle, allowing better understanding of intracellular transport and ciliogenesis.

## Materials and methods

### *Tetrahymena thermophila* cell culture

*Tetrahymena* cells were grown with moderate shaking (80 rpm) in SPP medium (1% proteose peptone, 0.2% glucose, 0.1% yeast extract, and 0.003% Fe-EDTA)^85^, supplemented with 1% antibiotic–antimycotic mix (Sigma-Aldrich, A5955) at 30 °C. To assort an introduced transgene, cells were cultured in a medium supplemented with the increasing concentrations of paromomycin (Sigma, P8692) or puromycin (Sigma, P9620). Prior to experiments, cells were grown in SPP medium without drugs for 24 h.

### Immunostaining, mounting samples, and imaging

*Tetrahymena* cells (10 μl drop) were permeabilized on coverslips with 0.5–2% Triton X-100 in PHEM buffer (60 mM PIPES, 25 mM HEPES, 10 mM EGTA, 2 mM MgCl_2_, pH 6.9), for 45–60 s, fixed with 4% paraformaldehyde (Sigma, P6148) and after drying blocked with 3% BSA (Sigma, A9647) with 0.02% Tween (Sigma, P7949) in PBS for 10 min at room temperature.

For confocal microscopy, cells were incubated with primary antibodies (Supplementary Table S4) in the blocking solution (3% BSA + 0.02% Tween in PBS) overnight at 4 °C. Cells were washed 3 times with PBS and then incubated with the secondary antibodies (Supplementary Table S5) in the blocking solution for 1 h at room temperature in the dark. After washing with PBS, samples were counterstained with DAPI and mounted on glass slides in ProLong Gold (Life technologies, P36930) mounting media, and sealed with nail polish. Cells were imaged on the Olympus FV1000 confocal system using the 60x/1.42 oil lens.

To determine the radial measurements of proteins using 2D-single-colour STORM, cells were either indirectly labelled with primary antibodies for 1 h at room temperature followed by AlexaFlour 647 secondary antibody (ThermoFischer, A21235 or A27040) for 1 h at room temperature, or directly labelled with anti-HA conjugated with AlexaFlour 647 (Cell Signalling, 3444) for 1 h at room temperature in the dark.

STORM two color imaging can be challenging due to chromatic aberration^86^, which becomes significant at high resolution, and the fact that STORM fluorophores differ in buffer requirements. Therefore, to determine the axial position of the proteins of interest with respect to the reference anti-NUP50 antibody (Supplementary Table S4), we replaced the two-color studies by localizing proteins relative to our reference using single fluorophore (Alexa Fluor 647). Therefore, samples were incubated simultaneously with AlexaFluor 647-conjugated-anti-HA antibody to detect the tagged protein of interest and anti-NUP50. After washing samples were incubated overnight at 4 °C with anti-rabbit IgG-AlexaFluor 647 secondary antibody to detect anti-NUP50. Next, samples were washed (3 × PBS), postfixed with 4% paraformaldehyde or 4% paraformaldehyde with 0.1% glutaraldehyde for 10 min at room temperature, washed at least 5 times in 1xPBS, 10 min each, and mounted on depression slides containing 75 µl of GLOX buffer (100 mM glucose, 40 µg catalase (Sigma, C9322), 100 µg glucose oxidase (Sigma, 49180) and 100 mM of cysteamine (MEA) (Sigma, M9768)). The coverslips were sealed with nail polish and imaged in the TIRF mode on the Nikon STORM system using a 100×/1.49NA ApoTIRF oil lens. The samples were imaged for a maximum of 3 h after sealing, as the intensity of the signal fades after that, and therefore the switching process becomes weaker and inaccurate.

### STORM microscopy

STORM imaging was performed on a NIKON Eclipse Ti inverted Microscope using the Apo TIRF 100×/1.49 NA oil lens equipped with 405 laser and 638 lasers. Images were acquired at 4.8 ms (128 × 128 pixels) or 9.2 ms (256 × 256 pixels, pixel size: 0.157646 µm) for 20,000–50,000 frames using the ixOn Ultra EMCCD camera (Andor Technology) in the TIRF mode with no binning. EM gain was set at 17 MHz—16-bit, EM gain multiplier at 300, and conversion gain multiplier at 3. The cells were first detected at a very low laser power (0.5–1% of the 638 nm laser) and then illuminated with 100% 638 nm laser to force fluorophores to photoswitch. Blinking events could be detected within a few seconds in most of the samples. When photoswitching reduced, the 405 nm laser was used to activate the remaining AlexaFluor 647 fluorophores. This was either controlled manually or by using the “Auto LP” option provided by the NIS-Elements software.

### Image reconstruction

STORM images were reconstructed using the NIS Elements Imaging Software, version 4.51.01, provided by Nikon. The acquired raw data (nd2 files) were run on NIS Elements with following settings: “Auto ROI”, minimum height 2500–3000 and maximum height 65,535; Andor Basline was set to 100, minimum width 200 nm, maximum width 400 nm, initial fit width 300 nm, maximum axial ratio 1.3, and maximum displacement of 1 pixel. At the end of the run, a “molecule list” file in a binary format (.bin) was generated and contained parameters including the coordinates and intensity of each blinking event. The lateral drift in the images was corrected during the analysis using the automatic drift correction option that is provided in the NIS Elements software. Using autocorrelation, the entire set of molecules is used to track the drift. The coordinates of the molecules after drift correction were also automatically stored in the molecules list at the end of the analysis. Images of the rings in all the figures were displayed as normalized Gaussians to appear as spots or blobs. The appearance of the images was adjusted using the “Advanced Gaussian Rendering” option in the NIS Elements software. The resolution achieved varied between 15 and 30 nm for each investigated protein.

### Estimation of average radial position using MATLAB

#### Identifying centers of rings

Coordinates of individual fluorophore blinking events were clustered using “k-means” method with k from 6 to 15. The optimal number of clusters was evaluated using silhouette criterion^87^. The clustering was then interactively validated for being adequate. Two clusters with lowest summarized intensity of fluorophore blinking events were removed, to exclude signal coming from the background noise. The centers of remaining clusters were fit with a circle, and the center of this circle was used as a center of the ring for further analysis. Only rings with the circular fit of cluster centers with goodness-of-fit R^2^ greater than 0.9 were used.

#### Radial measurement of individual rings

The radial distances of individual fluorophore blinking events from identified ring centers were calculated. Intensities of blinking events were summed at 2 nm intervals from the center, producing a histogram with radial distribution of signal for each ring. This histogram was then fitted with either Gaussian distribution or circularly convolved Gaussian distribution^88^. Means of the best-fit distributions corresponded to radii of individual rings, and standard deviations to their width. Only the rings with a goodness-of-fit R^2^ of Gaussian distribution of 0.8 or more were used for statistical analysis and generation of an averaged ring. To generate an averaged ring, radial distribution of individual rings was summed and normalized to the number of rings. The resulting distribution was fitted with either Gaussian distribution or circularly convolved Gaussian distribution to obtain the radius and width of the averaged ring. The summed distribution of signal and the profiles of best-fit distribution were plotted radially from a center of empty images to create a visual representation of averaged rings.

### Estimation of axial positions using MATLAB

To determine the distance between two parallel rings the individual fluorophore blinking events were split into two clusters. As in this case clusters are not spherical, individual fluorophore blinking events were fit with a Gaussian mixture model. Then the clustering was interactively validated for being adequate. The brighter of two clusters was fit with an ellipse with individual blinking events being weighted proportionally to their intensity. The center of each dataset was calculated as a mean of cluster centers. Then each dataset was shifted and rotated. Namely, the coordinates of individual blinking events were shifted so that the new center of each dataset corresponded to coordinates of origin and rotated so that the major axis of the brighter cluster was aligned with X-axis.

Intensities of blinking events were summed at 2 nm intervals along X- and Y-axis, producing histograms of signal distributions along each axis. Y-axis histogram was used to fit the signal with a sum of two Gaussian distributions. The distance between means of two distributions corresponded to the distance between two rings. Only the datasets with the goodness-of-fit R^2^ being 0.7 or more were used for statistics and to generate a summarized image. The Y- and X-axes distributions of individual rings were summed and normalized to the number of datasets. The outer product of summarized distributions was plotted to produce an image of an averaged dataset.

The summarized Y-axis distribution was fit with a sum of two Gaussian distributions. The distance between mean of distributions corresponded to the distance in averaged dataset between two parallel rings.

### Estimation of linkage error using MATLAB

To estimate the uncertainty of epitope location we used the information from the distance between positions determined using directly labelled primary antibody and those determined with primary antibody followed by secondary antibody staining. We assumed the position of the directly labelled primary antibody being 6 nm away from the epitope based on the length of antibody molecule being 12 nm^89^ and the signal coming from multiple labelled sites uniformly distributed along the antibody length. For the secondary antibody we assumed that its center is 6 nm away from the end of the primary antibody opposite of the epitope. Note that such end-to-end binding assumption is likely to overestimate the error and estimates the maximum uncertainty. Then for each angle between primary antibody and epitope we calculated all possible distances between primary and secondary antibody for coplanar geometry. As above, such geometry provides the estimate of maximum uncertainty, i.e. distance between the center of primary antibody and the epitope. We collected all angles between primary antibody and the epitope where the observed distance between primary and secondary antibody was possible and calculated the range of possible positions of the epitope relatively to the primary antibody from these angle range. The minimal localization accuracy was about 2 nm in most of our experiments in all the lines investigated in this study.

### Clusters/spots counting and analysis

To assess the differences in the numbers of clusters/blobs in each ring between controls and mutant lines of TMEM216, IFT54, and IFT81, we chose at least 5 cells per line per experiment (at least 2 independent experiments). The longest row of cilia bases cells was manually picked in each cell and all individual rings in the row were chosen for further analysis. To analyze the number of clusters in each ring, the molecules list (.txt) of each ring was extracted using the NIS Elements software. The txt files were then converted into csv files and run on custom MATLAB code to determine the number of clusters in each ring. The number of clusters was confirmed manually in all the rings. The % relative frequency of the number of clusters in each ring for every experiment was calculated for control and mutant lines. The obtained data were plotted in bar graphs to show the shift in the number of clusters/ring where applicable.

### Blender

Models shown in Figs. 1, 2, and 4 were generated using Blender 2.79: https://www.blender.org/download/Blender2.79/blender-2.79b-windows64.msi/.

Rotating 3-D model in Fig. 4.
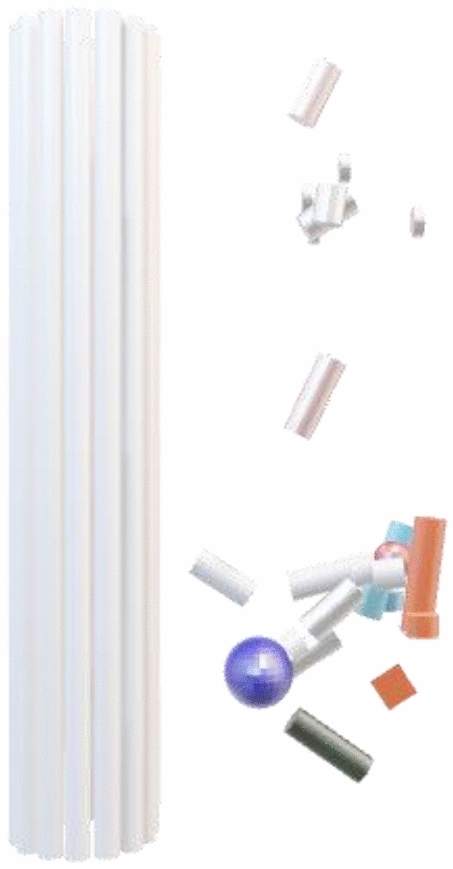


### Statistical analysis

Statistical analyses were carried out using *t*-test and Mann–Whitney test included in GraphPad Prism 7.0 software (http://www.graphpad.com/). Data are presented as mean ± 95% confidence interval (95% CI), standard deviation (SD), or standard error of the mean (SEM) as indicated. Statistical significance is indicated as follows: for *p* < 0.05, *;* p* < 0.01, **; *p* < 0.001, ***; *p* < 0.0001, ****. Graphs in Figs. 1, 2 and 3 were prepared using GraphPad Prism 7.0 software, and Adobe photoshop (CS6) was used to prepare all figures.

## Supplementary Information

Supplementary Information.

## Data Availability

Data generated or analysed during this study are included in the manuscript (and its Supplementary Information files). All MATLAB scripts used for the analyses of the data are available at https://github.com/nbul/STORM under a BSD 3-Clause License.
