# Author Correction: STORM imaging reveals the spatial arrangement of transition zone components and IFT particles at the ciliary base in Tetrahymena

**DOI:** 10.1038/s41598-022-08118-y

**Published:** 2022-03-07

**Authors:** Khodor S. Hazime, Zhu Zhou, Ewa Joachimiak, Natalia A. Bulgakova, Dorota Wloga, Jarema J. Malicki

**Affiliations:** 1 Bateson Centre and the Department of Biomedical Science, University of Shefeld, Western Bank, Shefeld, S10 2TN UK; 2 Nencki Institute of Experimental Biology Polish Academy of Sciences - Laboratory of Cytoskeleton and Cilia Biology, 3 Pasteur Street, 02-093 Warsaw, Poland

Correction to: *Scientific Reports* 10.1038/s41598-021-86909-5, published online 12 April 2021

The original version of this Article contained an error in Affiliation 2, which was incorrectly given as ‘Laboratory of Cytoskeleton and Cilia Biology, 3 Pasteur Street, 02-093, Warsaw, Poland’. The correct affiliation is listed below:

Nencki Institute of Experimental Biology Polish Academy of Sciences - Laboratory of Cytoskeleton and Cilia Biology, 3 Pasteur Street, 02-093 Warsaw, Poland.

The original Article has been corrected.

