# Supplementary material for: STORM imaging reveals the spatial arrangement of transition zone components and IFT particles at the ciliary base in Tetrahymena
Source: Sci Rep. 2021 Apr 12;11:7899. doi: 10.1038/s41598-021-86909-5 (PMC8041816; doi:10.1038/s41598-021-86909-5)
Supplement: Supplementary file 1 — Supplementary Information. [file 41598_2021_86909_MOESM1_ESM.docx]

**STORM Imaging reveals the Spatial Arrangement of Transition Zone Components and IFT Particles at the ciliary base in Tetrahymena**

Khodor S. Hazime^1^*, Zhu Zhou^1^, Ewa Joachimiak^2^, Natalia A. Bulgakova^1^*, Dorota Wloga^2^*, Jarema J. Malicki^1✝^

1 University of Sheffield Bateson Centre and the Department of Biomedical Science Western Bank Sheffield, S10 2TN, United Kingdom

2 Nencki Institute of Experimental Biology Polish Academy of Sciences, Laboratory of Cytoskeleton and Cilia Biology, 3 Pasteur Street, 02-093 Warsaw, Poland

* corresponding authors: [khodor.hazime@manchester.ac.uk](mailto:khodor.hazime@manchester.ac.uk), [n.bulgakova@sheffield.ac.uk](mailto:n.bulgakova@sheffield.ac.uk), d.wloga@nencki.edu.pl

✝ deceased

**Supplementary methods**

**Genomic DNA extraction**

*Tetrahymena* cells were grown to a final density of 3-5 x 10^5^ cells/mL and starved in 10 mM Tris-HCl (pH 7.4) for 18-24 hr. Approximately 10^7^ cells were spun at 1600 g for 5 min, resuspended in 10 mM Tris-HCl pH and lysed by addition of 7 volumes of Urea lysis buffer (42% urea, 0.35 M NaCl, 0.01 M Tris pH, 7.5, 0.01 M EDTA, and 1% SDS) [1]. Cell lysate was extracted with an equal volume of phenol:chloroform:isoamyl alcohol (25:24:1) and spun at 3000 g for 15 min. The collected water phase containing genomic DNA was extracted again with equal volume of phenol:chloroform:isoamyl alcohol (25:24:1) and next with equal volume of chloroform:isoamyl alcohol (24:1). The genomic DNA was precipitated by addition of NaCl to final concentration of 1 M and isopropyl alcohol to final concentration of 50%. The DNA precipitate was washed with 70% ethanol, air-dried, and resuspended at 55^O^C overnight in TE buffer supplemented with RNase A (10mg/mL RNAseA – ThermoFischer, EN0531).

**Knock-in constructs**

The fragments of the genes of interest were amplified using Phusion Hot Start II DNA polymerase (Thermo Fisher Scientific, Baltics, Lithuania, F549L). The PCR products were digested with appropriate restriction enzymes (Supplementary Table 6) and cloned into plasmids enabling the expression of C-terminally 3HA [2] or GFP-tagged [3] proteins under the control of the respective native promoters. To express N-terminally 3HA-tagged proteins in the native locus, approximately 1 kb fragment of the 5’UTR (with its 3’ end about 100 bp upstream to ATG) and 1 kb fragment with ATG of the coding region were amplified by PCR with the addition of the appropriate restriction sites (Supplementary Table 6). Both PCR products were cloned into pBlueScript II KS(+) vector and separated by a fragment of DNA containing neo2 cassette [4] positioned in the reverse orientation to the amplified coding region followed by 0.9 kb fragment of the MTT1 promoter [5] and a 3HA coding region. The neo2 cassette enabled selection of the transformed *Tetrahymena* cells. Before transformation plasmids were digested with the restriction enzymes to separate transgene from the plasmid backbone.

***Tetrahymena* cells transformation**

The biolistic transformation was carried out as described previously [1] using 10 µg of the digested plasmid DNA precipitated onto DNAdel Gold Carrier Particles (Seashell Technology, La Jolla, CA, USA) according to the manufacturer’s instructions and Biolistic PDS-1000/He Particle Delivery System (Bio-Rad). Cells transformed with a transgene enabling C-terminal tagging were selected in SPP medium supplemented with paromomycin (120 µg/mL) and CdCl_2_ (1 µg/mL) and cells transformed with a transgene enabling N-terminal tagging were selected on paromomycin (100 µg/mL) for 72 hr at 30^O^C. Next, selected clones were grown in SPP medium supplied with increasing concentrations of paromomycin (and decreasing concentration of CdCl_2_ in case of C-terminal tagging) to promote phenotypic assortment.

**Protein extraction, SDS-PAGE, and Western Blot**

Protease inhibitors (cOmplete^™^ Mini, EDTA-free Protease Inhibitor Cocktail (Roche)) were added to all buffers used in protein extraction procedure. *Tetrahymena* cytoskeletal proteins were isolated as previously described [6]. Briefly, *Tetrahymena* cells (approximately 3-5 x 10^6^ cells) were spun down, washed with 10 mM Tris-HCl, pH 7.4, mixed with equal volume of 2x lysis buffer (1.4 M sucrose, 4 mM EGTA, 4 mM EDTA, 20 mM Tris buffer, pH 8) and incubated for 1-2 min. on ice. Next, the cytoskeletal proteins were pelleted at 16,000 g for 10 min at 4^O^C, washed with 10 mM Tris-HCl, pH 7.4 and collected by centrifugation as before. The pellet was resuspended in 10 mM Tris-HCl, pH 7.4 and used for further analysis. Protein concentration was estimated using Pierce^TM^ BCA Protein Assay Kit (Thermo Scientific). Approximately 50 µg of cytoskeletal proteins were separated on SDS-PAGE gel and transferred into nitrocellulose membranes. The blots were blocked in 5% non-fat milk in TBST (1x TBS + 0.1% Tween) for 1 hr at RT and then incubated with mouse anti-HA (1:2000; Covance, MMS-101R) or rabbit anti-GFP (1:60 000, Abcam) primary antibody O/N at 4^O^C. After washings with TBST (4x 15 min RT), blots were incubated with the secondary antibody (HRP-conjugated anti mouse IgG 1:10,000 or anti-rabbit IgG 1:20 000, Jackson) for 1hr at RT. ECL substrate Westar ηC Ultra 2.0 (Cyanagen, Bologna, Italy) was added and chemiluminescent signals were detected using G:box XT4 Chemiluminescence and Fluorescence Imaging System (Syngene).

**Deciliation (pH Shock method) and cilia regeneration**

*Tetrahymena* cells were deciliated as previously described [7]. Briefly, cells from the logarithmic phase were spun down, washed with 10 mM Tris-HCl, pH 7.4, and resuspended in 20 mL of deciliation buffer (10 mM Tris-HCl, pH 7.4, 50 mM sucrose, 10 mM CaCl_2_, with protease inhibitors) in an Erlenmeyer flask. To shed cilia, cells were exposed to 45 sec to the pH shock caused by the addition of acetic acid (final concentration 0.012 M) followed by the neutralization with KOH (final concentration 0.011 M). During the pH shock cells were gently mixed and deciliation was controlled under light microscope (Zeiss Axio Lab1). Deciliated cells were either fixed and immunostained or allowed to regenerate cilia for 30-60 min in Tris-HCl (pH 7.4) before fixation, immunostaining, and imaging.

**Fluorescence Recovery after Photobleaching (FRAP) Imaging**

*Tetrahymena* cells carrying a transgene enabling expression of one of the following proteins: IFT52-GFP, IFT144-GFP, or GFP-MKS6 were spun at 500g for 3min, mixed with 3 volumes of the 3% low melting agarose prepared in 1x PBS and cooled down to RT and placed as one drop on a glass slide and quickly covered with a coverslip. The coverslip was pressed gently, and samples were allowed to solidify 10-15 min at RT. Cells were first examined using light microscope to ensure that they were not damaged and next FRAP imaging was performed using LSM 880 Airyscan Zeiss inverted microscope using 1.49NA 60x oil lens. The IFT docking sites (for IFT52-GFP or IFT144-GFP) or transition zones (GFP-MKS6) were selected using the ROI (region of interest) option in ZEN software. The ROIs were then simultaneously excited with 100% 488 nm and 50-100% 405 nm lasers to bleach the ROIs. Five images were obtained prior to photobleaching for 5 sec followed by bleaching the ROIs. Images were then acquired every 2.5 sec for 250 sec. To exclude the effect of acquisition photobleaching, a nonphotobleached ROI was selected throughout the acquisition. The average signal in the bleached and unbleached ROIs was determined at each time point. The intensities were normalized for acquisition bleaching for each ROI and were used to generate recovery curves.

**Supplementary** **Figure 1 – Alignments of proteins investigated in this study**

**AHI1**


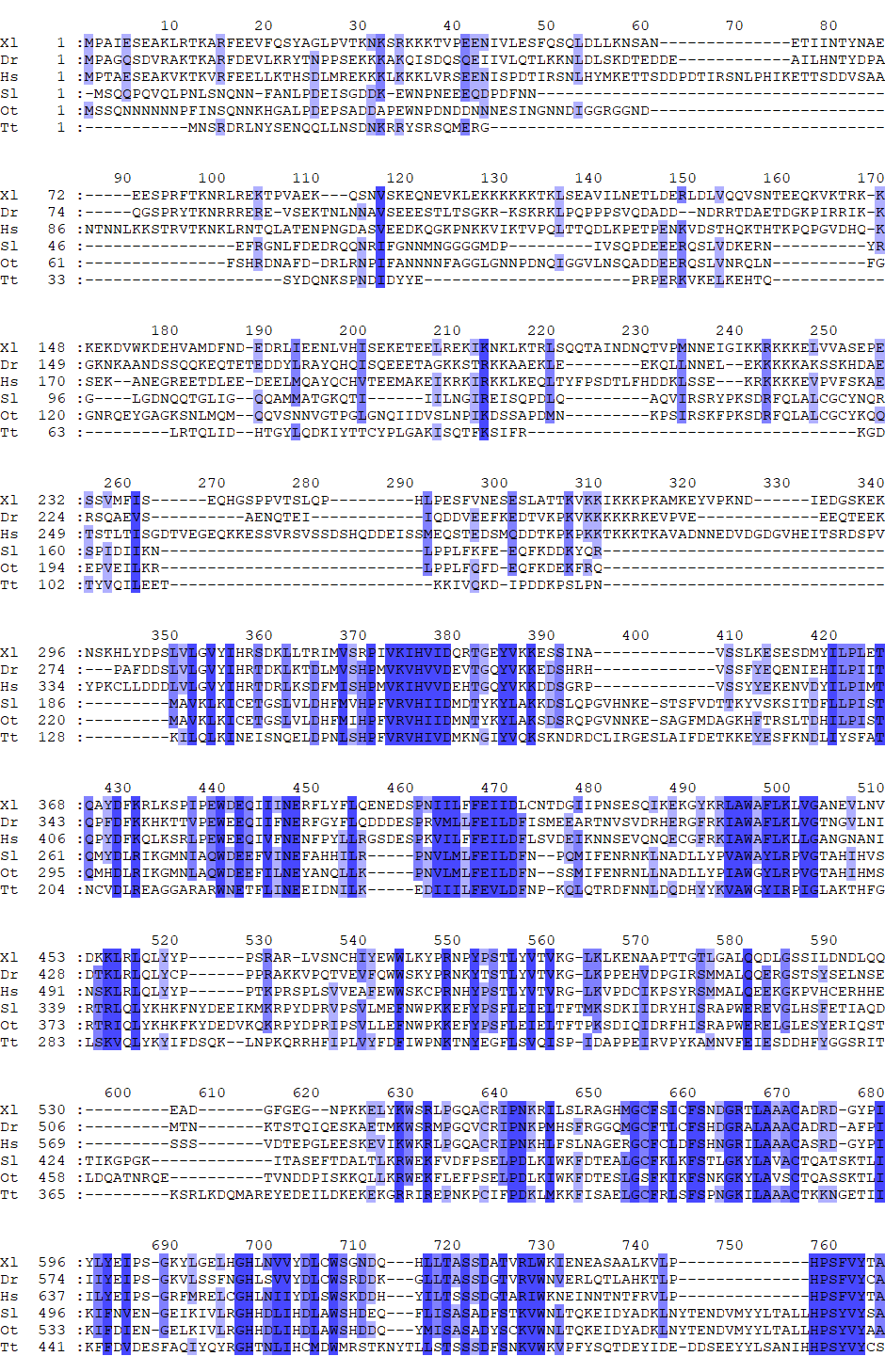


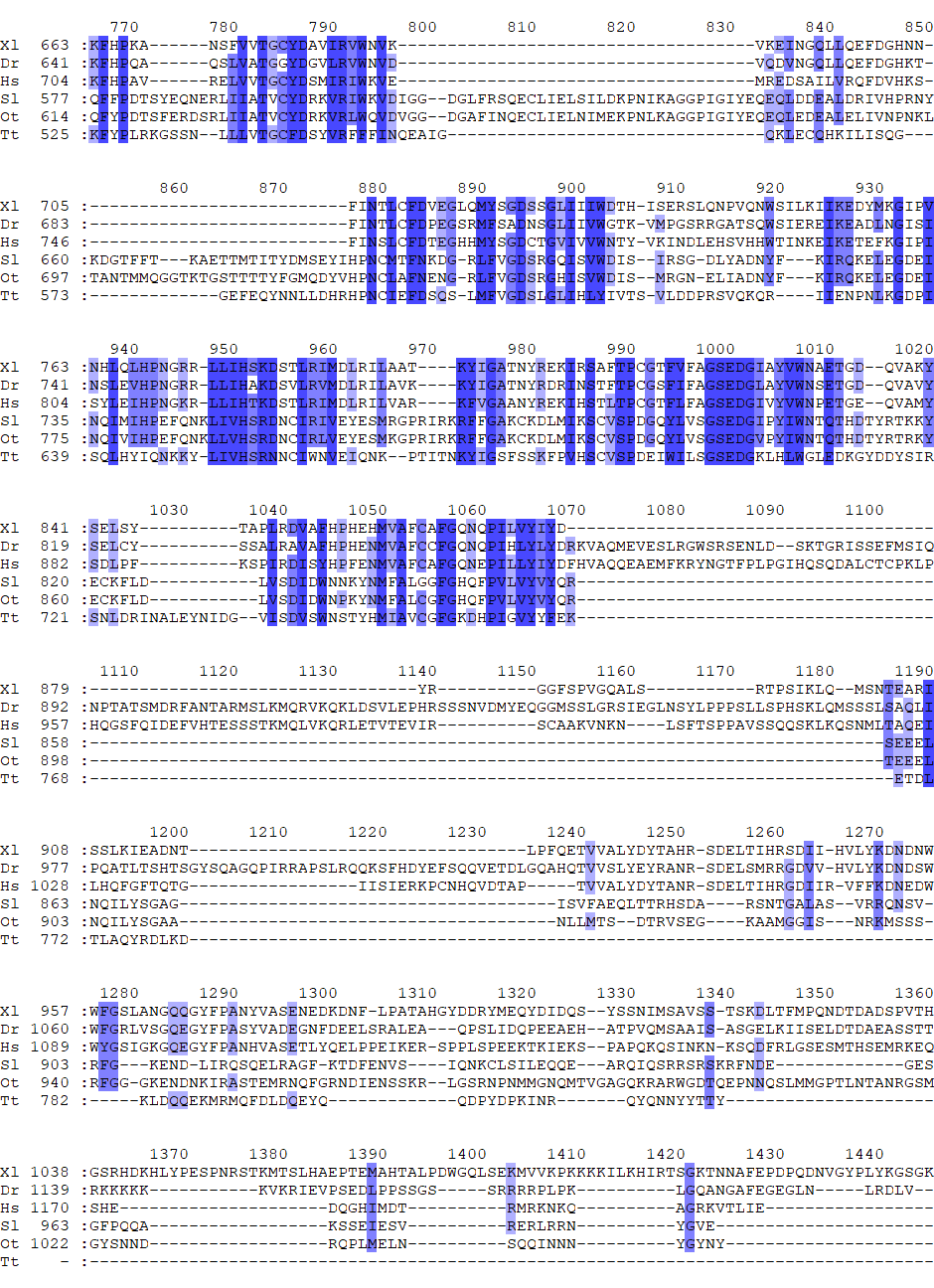


*Danio rerio* (Dr, NP_001306918.1), *Homo sapiens* (Hs, NP_060121.3), *Oxytricha trifallax* (Ot, EJY83061.1), Stylonychia lemnae (Sl, CDW91522.1 ), *Tetrahymena thermophila* (Tt, TTHERM_00460620), *Xenopus laevis* (Xl, OCT80288.1)

**B9D1**


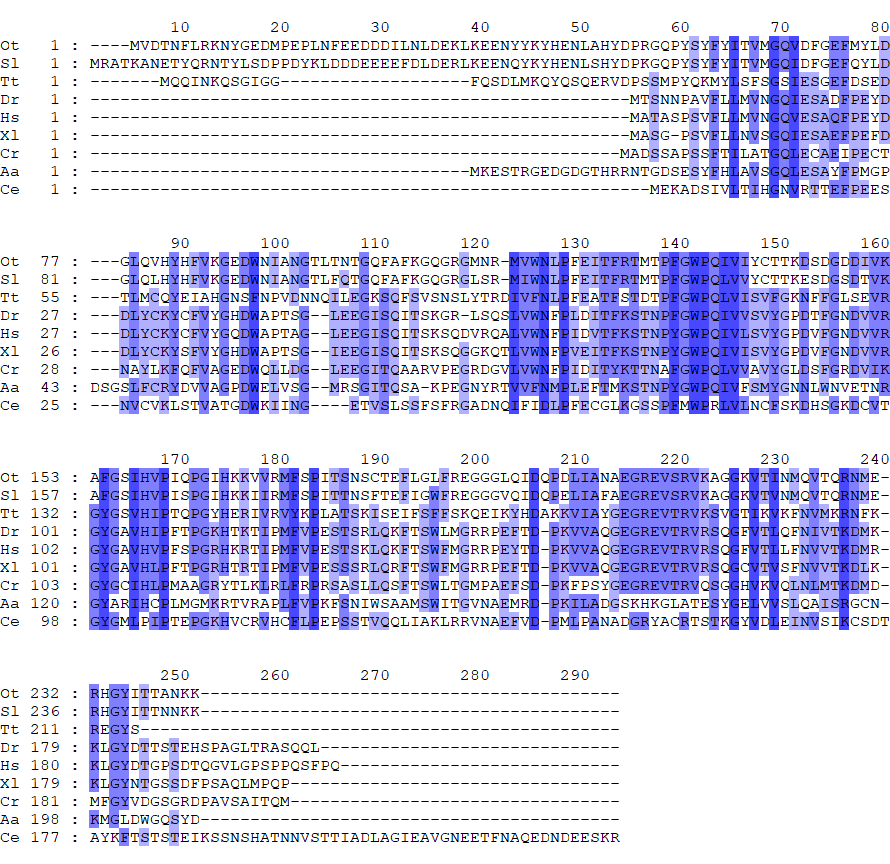


*Aedes aegypti* (Aa, XP_001657653.1), *Caenorhabditis elegans* (Ce, NP_508203.1), *Chlamydomonas reinhardtii* (Cr, XP_001695895.1), *Danio rerio* (Dr, NP_001019544.1), *Homo sapiens* (Hs, NP_056496.1), *Oxytricha trifallax* (Ot, EJY78509.1 B9), *Stylonychia lemnae* (Sl, CDW79625.1), *Tetrahymena thermophila* (Tt, TTHERM_00219110), *Xenopus laevis* (Xl, NP_001086557.1)

**MKS1**


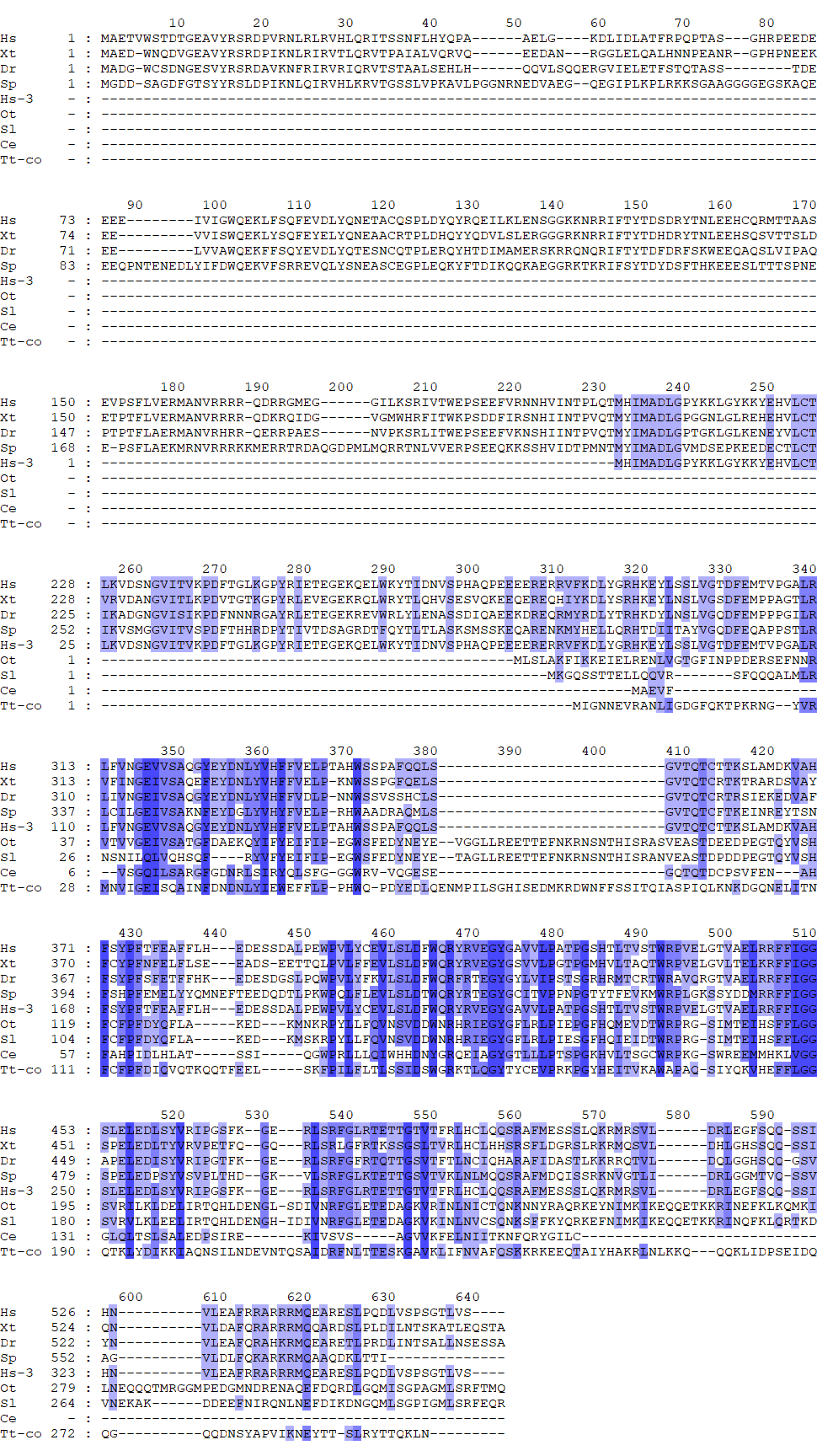


*Caenorhabditis elegans* (Ce, NP_500186.1), *Danio rerio* (Dr, NP_001070841.2), *Homo sapiens* (Hs, NP_060247.2, Hs-3, isoform 3, NP_001308197.1), *Oxytricha trifallax* (Ot, EJY76954.1), *Strongylocentrotus purpuratus* (Sp, XP_011678596.1), *Stylonychia lemnae* (Sl, CDW77997.1)*Tetrahymena thermophila* (Tt-co, TTHERM_00630490, prediction manually corrected), *Xenopus tropicalis* (Xt, XP_012812621.1)

**THERM_00630490 corrected** MIGNNEVRANLIGDGFQKTPKRNGYVRMNVIGEISQAINFDNDNLYIEWEFFLPPHWQPDYEDLQENMPILSGHISEDMKRDWNFFSSITQIASPIQLKNKDGQNELITNFCFPFDIQVQTKQQTFEELSKFPILFLTLSSIDSWGRKTLQGYTYCEVPRKPGYHEITVKAWAPAQSIYQKVHEFFLGGQTKLYDIKKIAQNSILNDEVNTQSAIDRFNLTTESKGAVKLIFNVAFQSKKRKEEQTAIYHAKRLNLKKQQQKLIDPSEIDQQGQQDNSYAPVIKNEYTTSLRYTTQKLN

**MKS6**


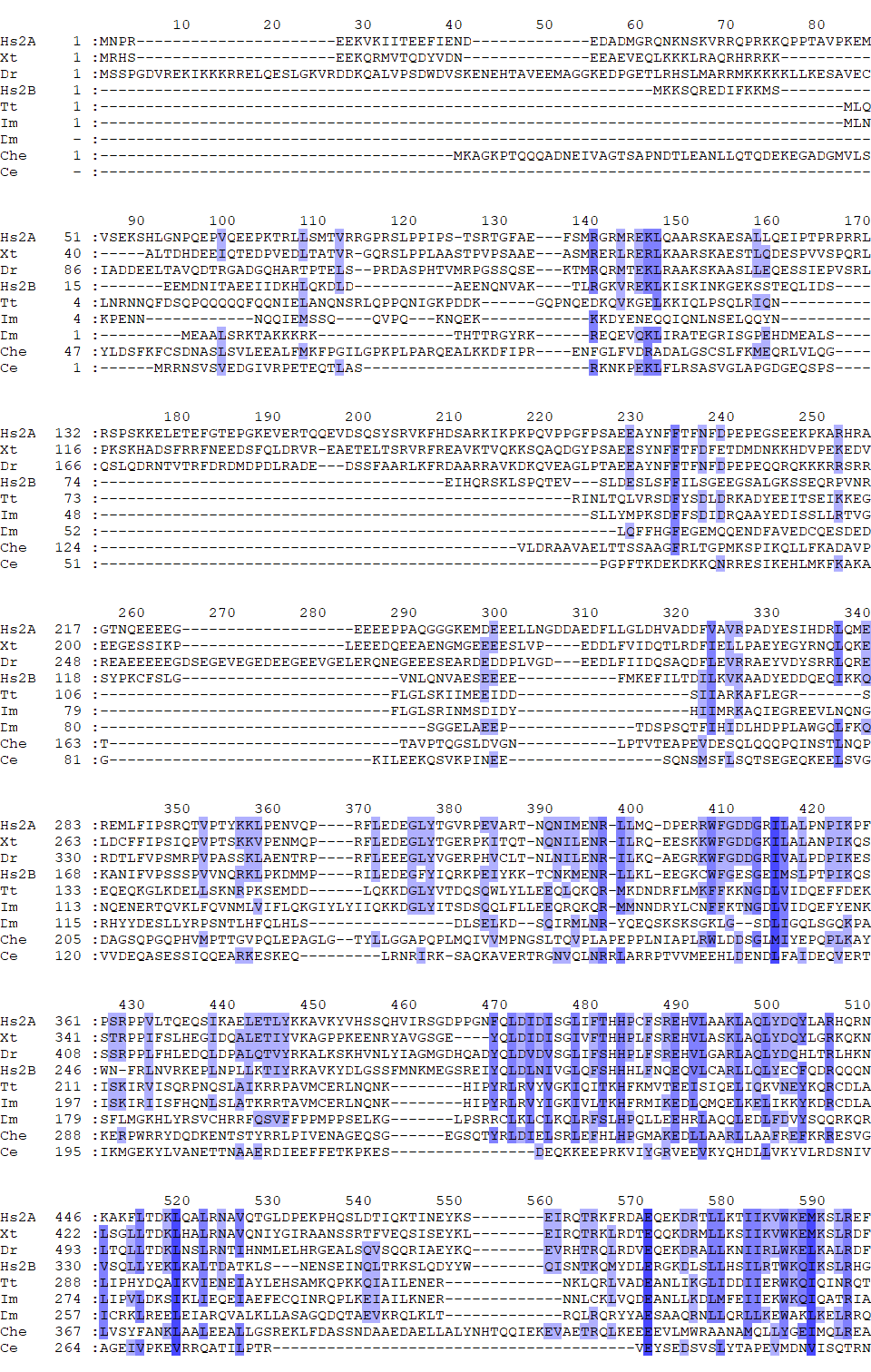


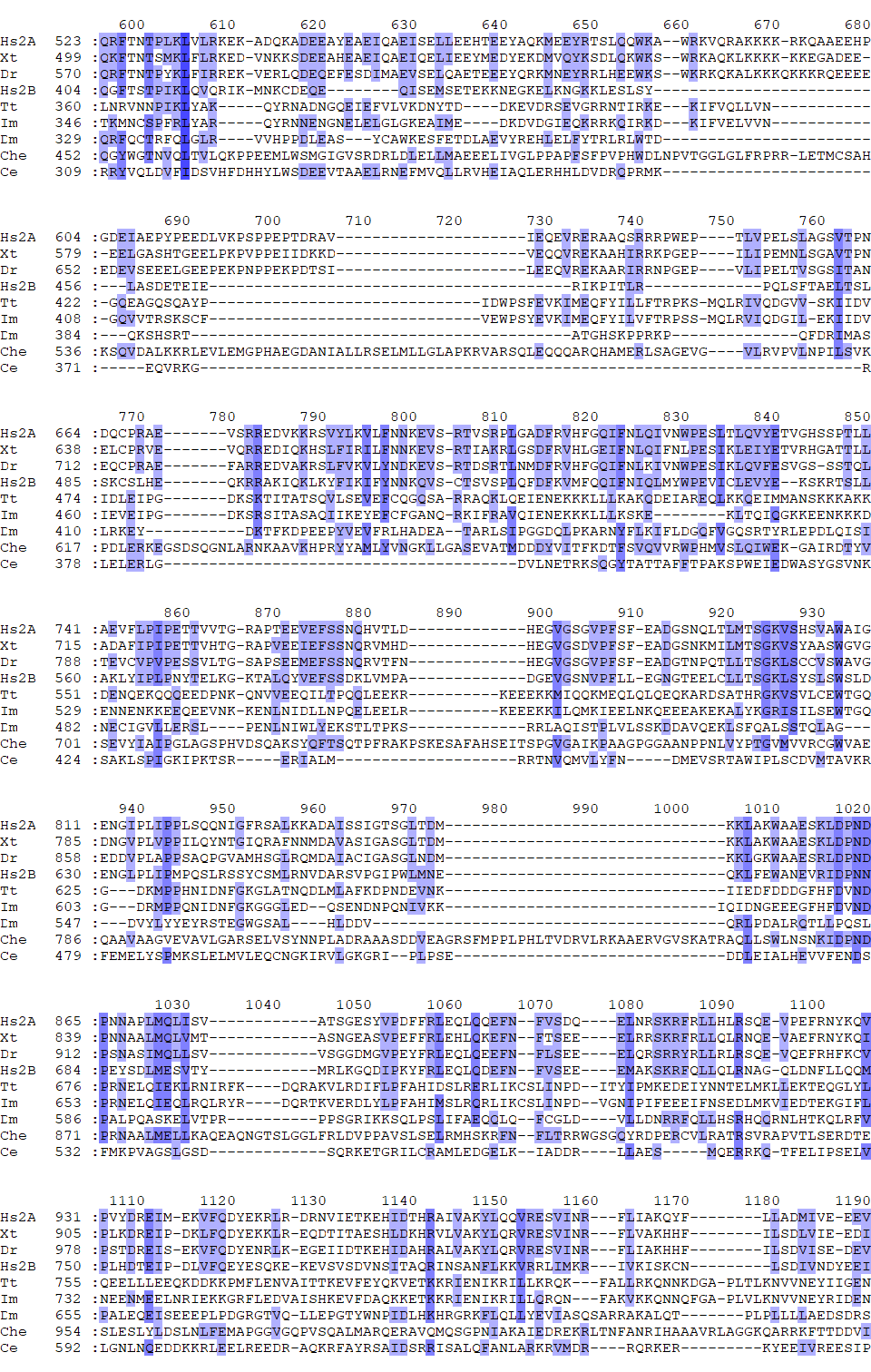


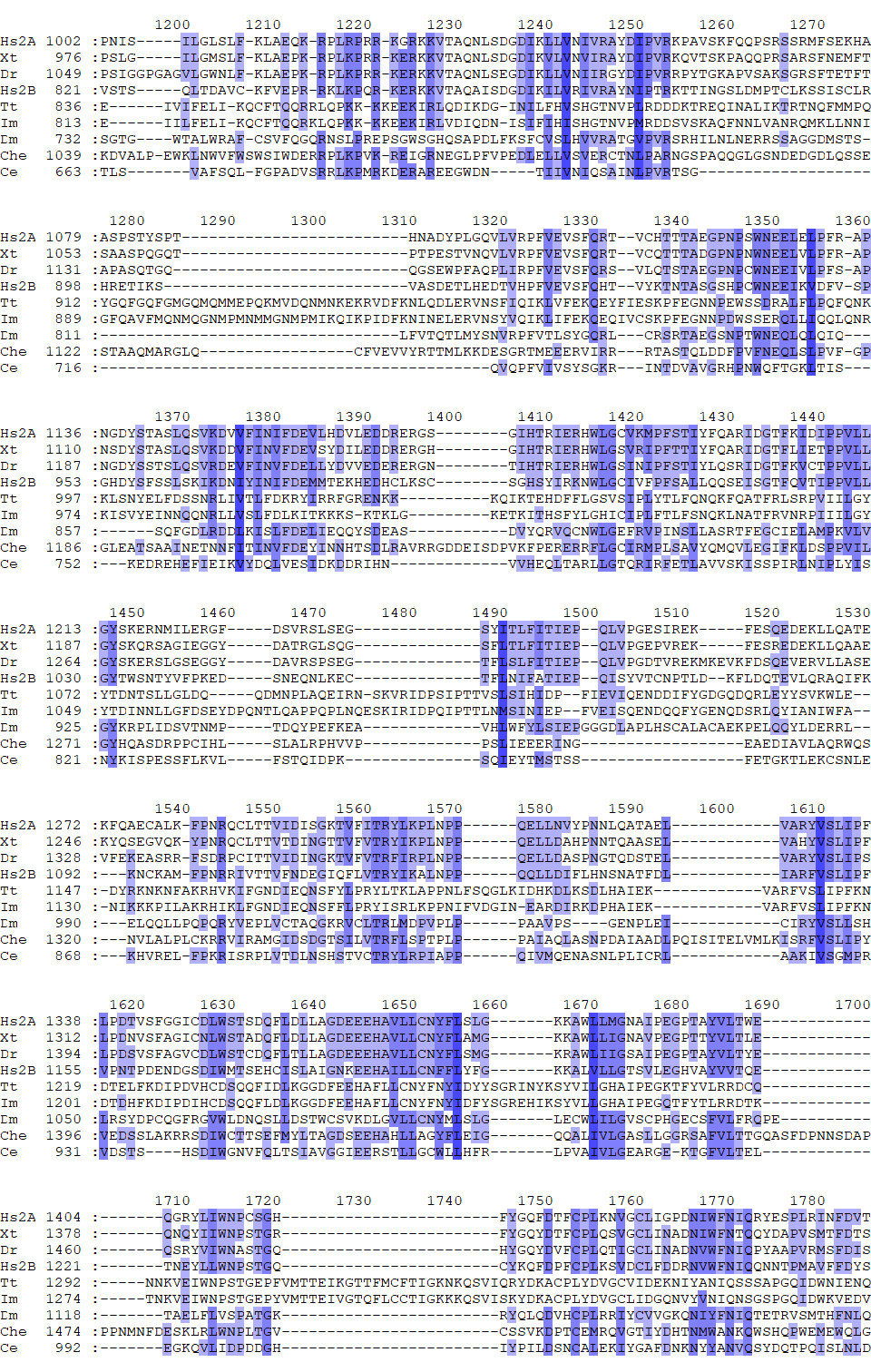


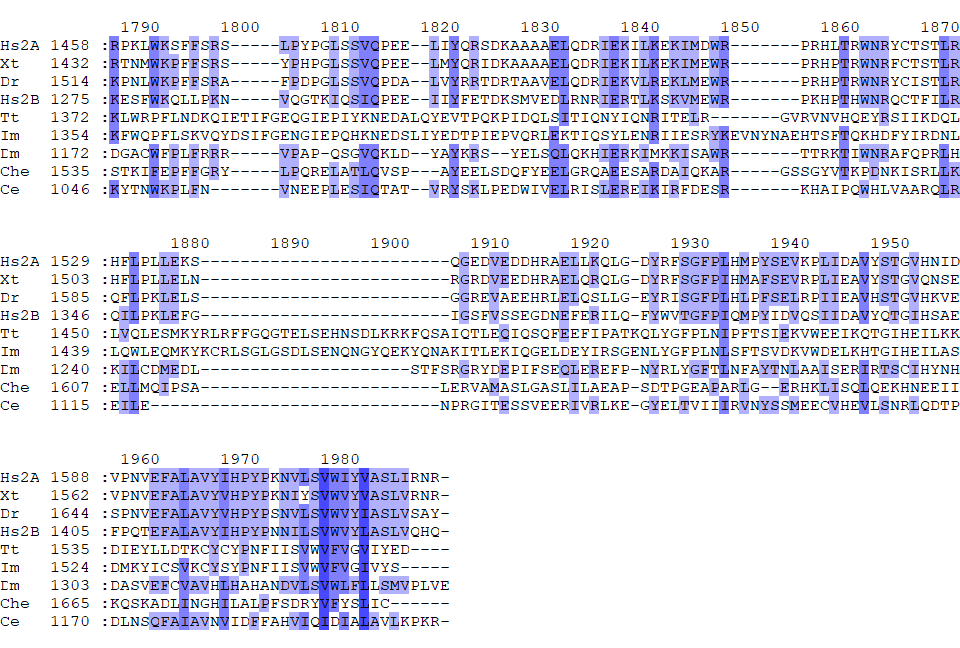


*Caenorhabditis elegans* (Ce, NP_492026.1), *Chlamydomonas eustigma* (Che, GAX76484.1), *Danio rerio* (Dr, XP_009294853.1), *Drosophila melanogaster* (Dm, NP_611229.2), *Homo sapiens* (Hs2A, NP_001073991.2, Hs2B, NP_001335937.1), *Ichthyophthirius multifiliis* (Im, XP_004027584.1), *Tetrahymena thermophila* (Tt, TTHERM_00348780), *Xenopus tropicalis* (Xt, XP_002938689.2)

**NPHP4**


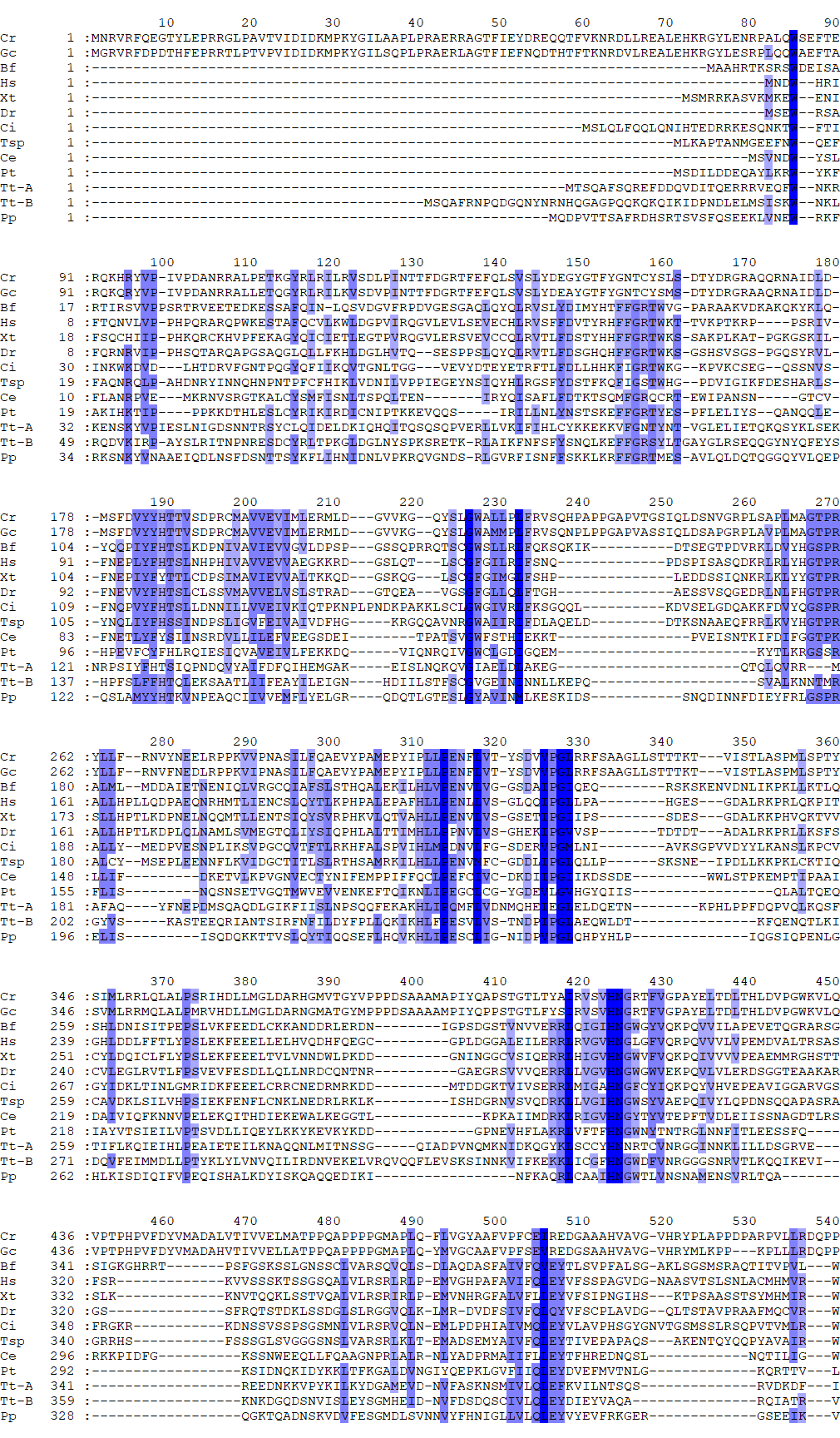


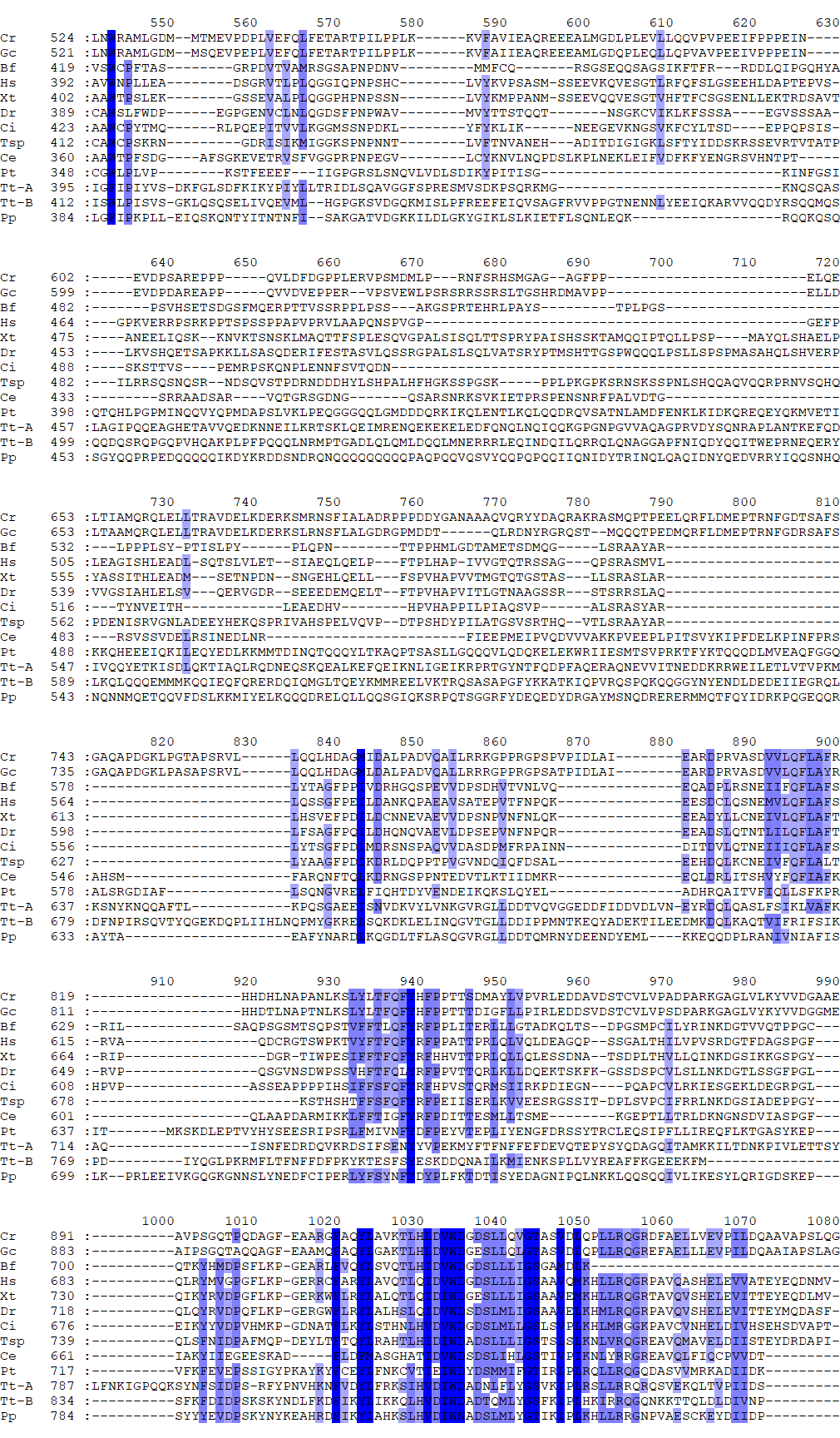


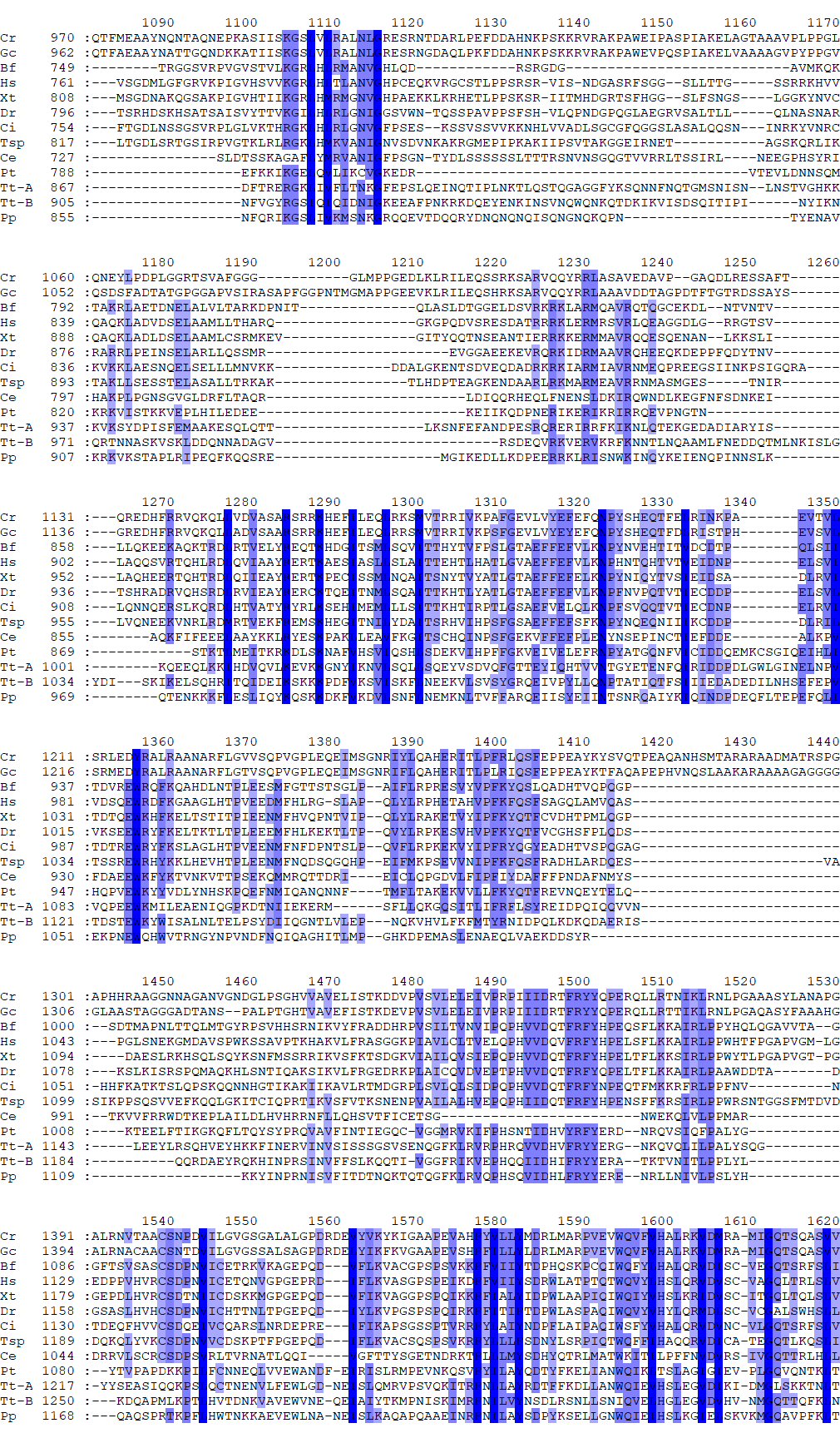


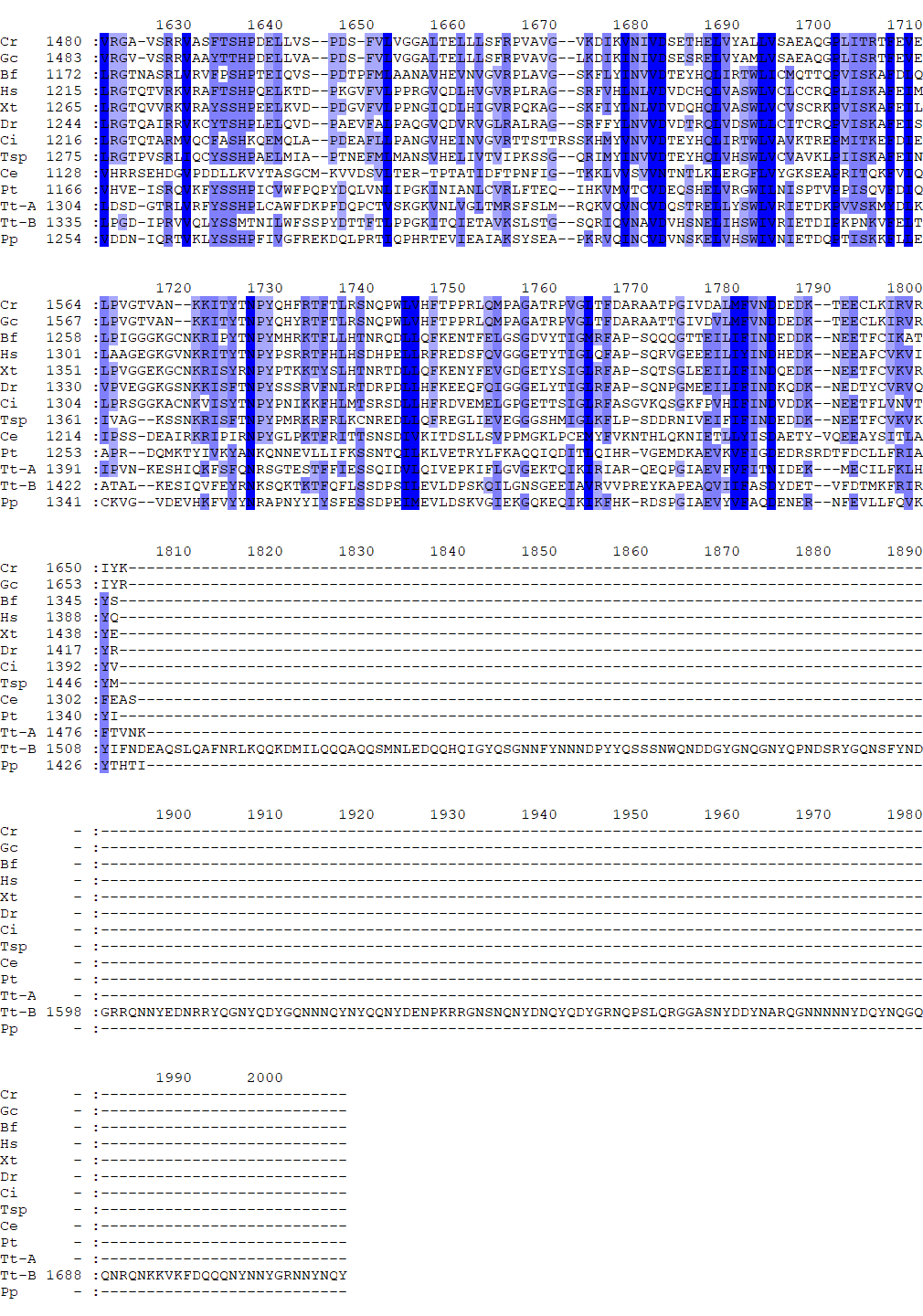


*Branchiostoma floridae* (Bf, [XP_002613821.1](https://www.ncbi.nlm.nih.gov/protein/XP_002613821.1?report=genbank&log$=protalign&blast_rank=1&RID=1GV979X601R)), *Caenorhabditis elegans* (Ce, NP_505982.2), *Chlamydomonas reinhardtii* (Cr, XP_001692920.1), *Gonium pectorale* (Gc, KXZ51260.1), *Homo sapiens* (Hs, [XP_011539517.1](https://www.ncbi.nlm.nih.gov/protein/XP_011539517.1?report=genbank&log$=protalign&blast_rank=1&RID=1GVA849201R)), *Paramecium tetraurelia* (Pt, XP_001442100)*, Pseudocohnilembus persalinus* (Pp, KRX06407.1), *Tetrahymena thermophila* (TtA, TTHERM_00148790; TtB, TTHERM_00653970), *Trichoplax* sp. H2 (Tsp, RDD44207.1), *Xenopus tropicalis* (Xt, OCA29395.1).

**TMEM216**


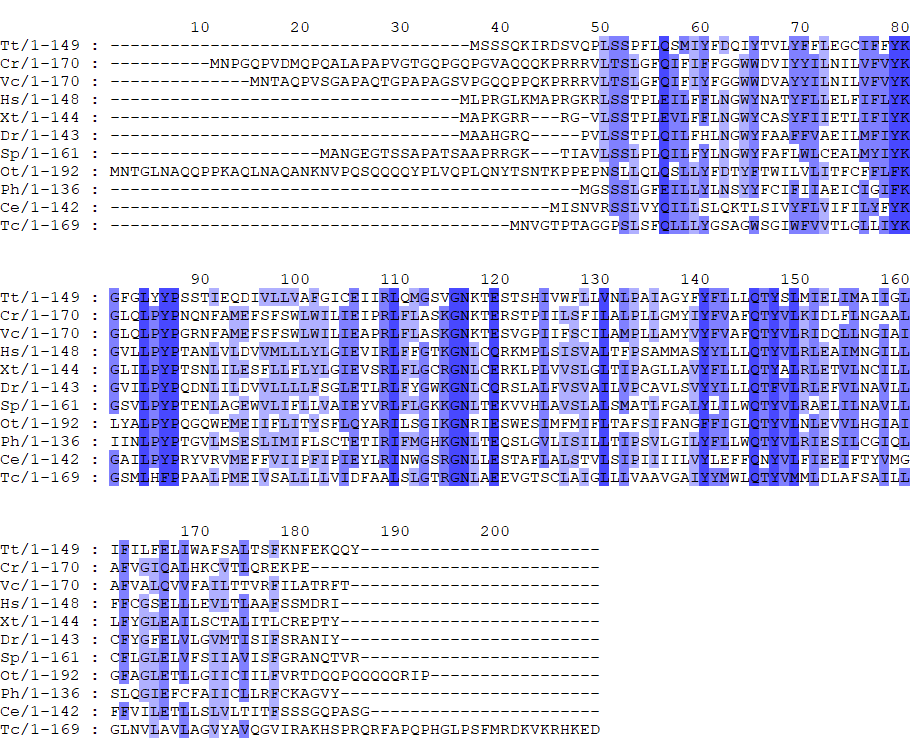


*Caenorhabditis elegans* (Ce, NP_001254108.1), *Chlamydomonas reinhardtii* (Cr, PNW80145.1, CHLRE_08g379750v5), *Danio rerio* (Dr, NP_001315069.1), *Homo sapiens* (Hs, NP_001167462.1), *Oxytricha trifallax* (Ot, EJY86232.1), *Pediculus humanus corporis* (Ph, XP_002423694.1), *Strongylocentrotus purpuratus* (Sp, [XP_011679034.1](https://www.ncbi.nlm.nih.gov/protein/XP_011679034.1?report=genbank&log$=protalign&blast_rank=1&RID=1B00051C01R)), *Tetrahymena thermophila* (Tt, TTHERM_00842510, [XP_001027267.1](https://www.ncbi.nlm.nih.gov/protein/XP_001027267.1?report=genbank&log$=protalign&blast_rank=1&RID=1AZC0EVX01R)), *Trypanosoma cruzi strain CL Brener* (Tc, XP_813387.1), *Volvox carteri f. nagariensis* (Vc, XP_002952957.1), *Xenopus tropicalis* (Xt, KAE8628293.1).

Red frame indicates the position of the ciliopathy-causing mutations: Hs L114R, L133X corresponding to Tt L113R, L132X.

**TMEM231**


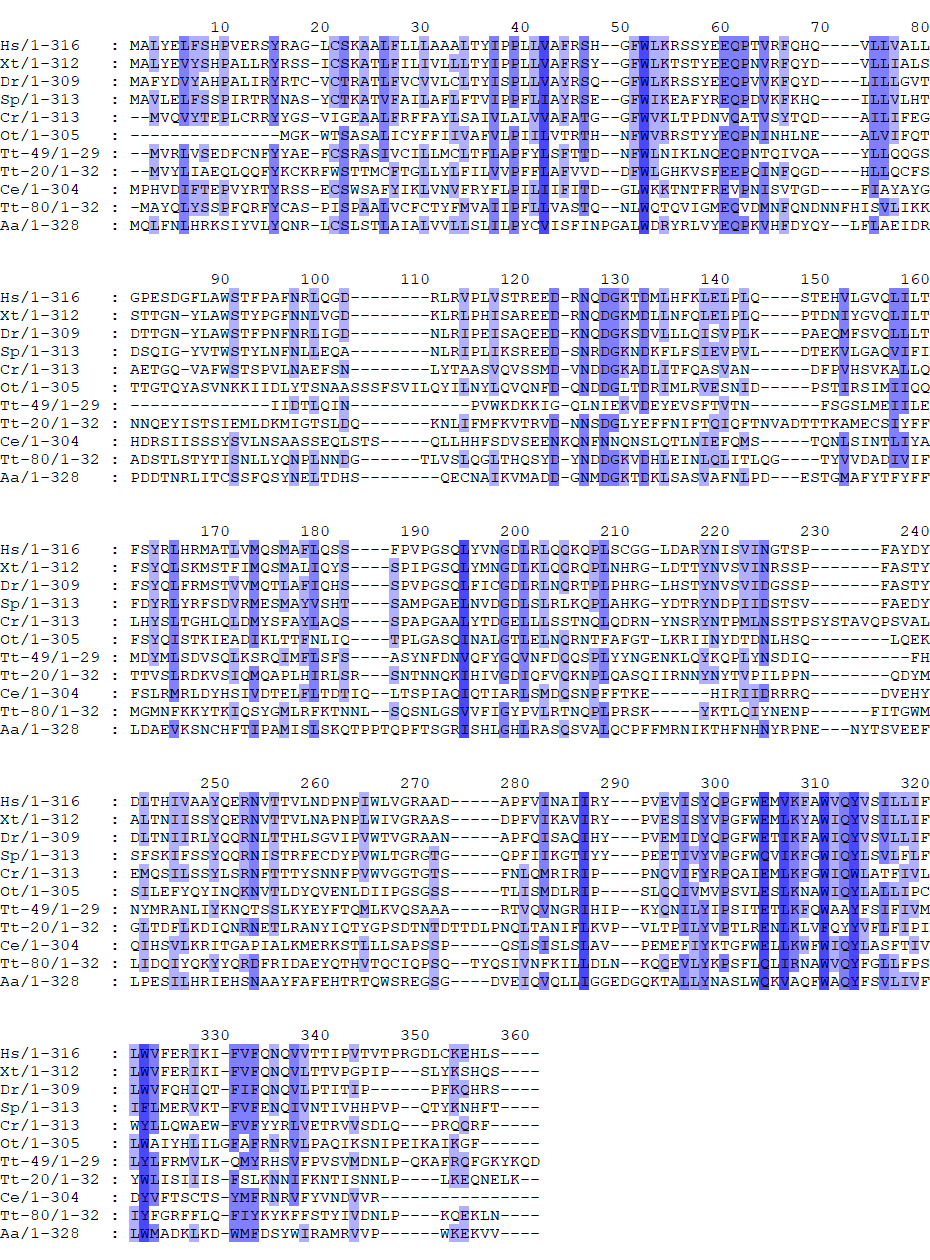


*Aedes aegypti* (Aa, XP_001661482.1), *Caenorhabditis elegans* (Ce, NP_501434.2), *Chlamydomonas reinhardtii* (Cr, XP_001690530.1), *Danio rerio* (Dr, NP_956681.1), *Homo sapiens* (Hs, NP_001070886.1), *Oxytricha trifallax* (Ot, EJY67475.1), *Strongylocentrotus purpuratus* (Sp, XP_011667727.1), *Tetrahymena thermophila* (Tt, TTHERM_000441849), *Tetrahymena thermophila* (Tt, TTHERM_00563980), *Tetrahymena thermophila* (Tt, TTHERM_00452020), *Xenopus tropicalis* (Xt, NP_001120108.1)

Red frame indicates the position of the ciliopathy-causing mutations: Hs I232SerfsX corresponding to Tt K209SerfsX).

**IFT27**


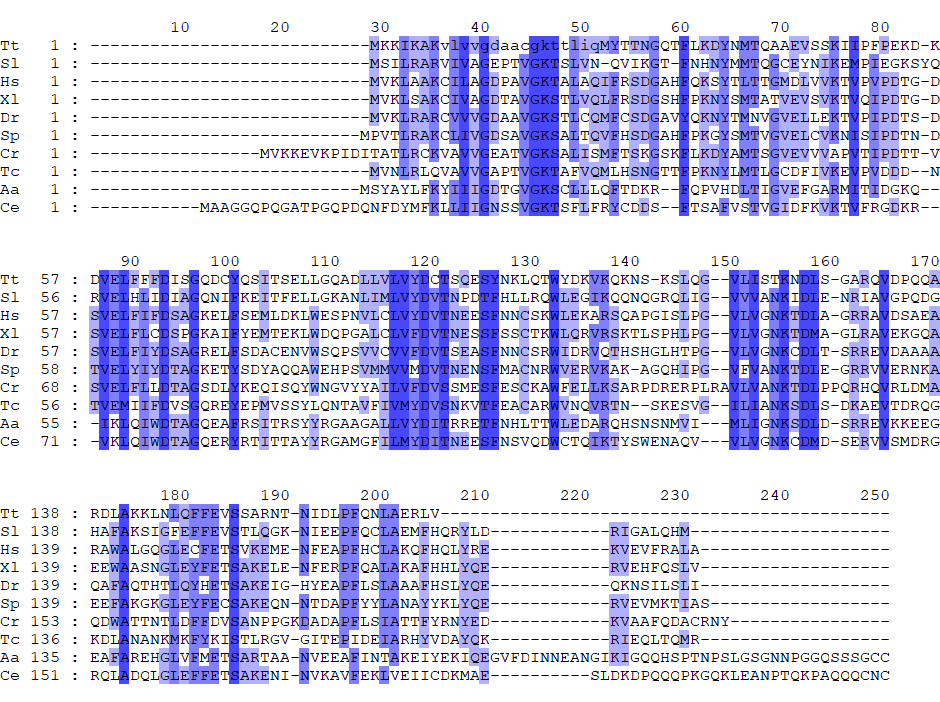


*Aedes aegypti* (Aa, XP_001650005.1), *Caenorhabditis elegans* (Ce, NP_001021974.1), *Chlamydomonas reinhardtii* (Cr, XP_001689745.1), *Danio rerio* (Dr, NP_001008588.1), *Homo sapiens* (Hs, NP_001171172.1), *Strongylocentrotus purpuratus* (Sp, XP_786289.1), *Stylonychia lemnae* (Sl, CDW76182.1), *Tetrahymena thermophila* (Tt, TTHERM_00298510), *Trypanosoma cruzi strain CL Brener* (Tc, XP_844145.1), *Xenopus laevis* (Xl, XP_018112476.1)

**IFT46**


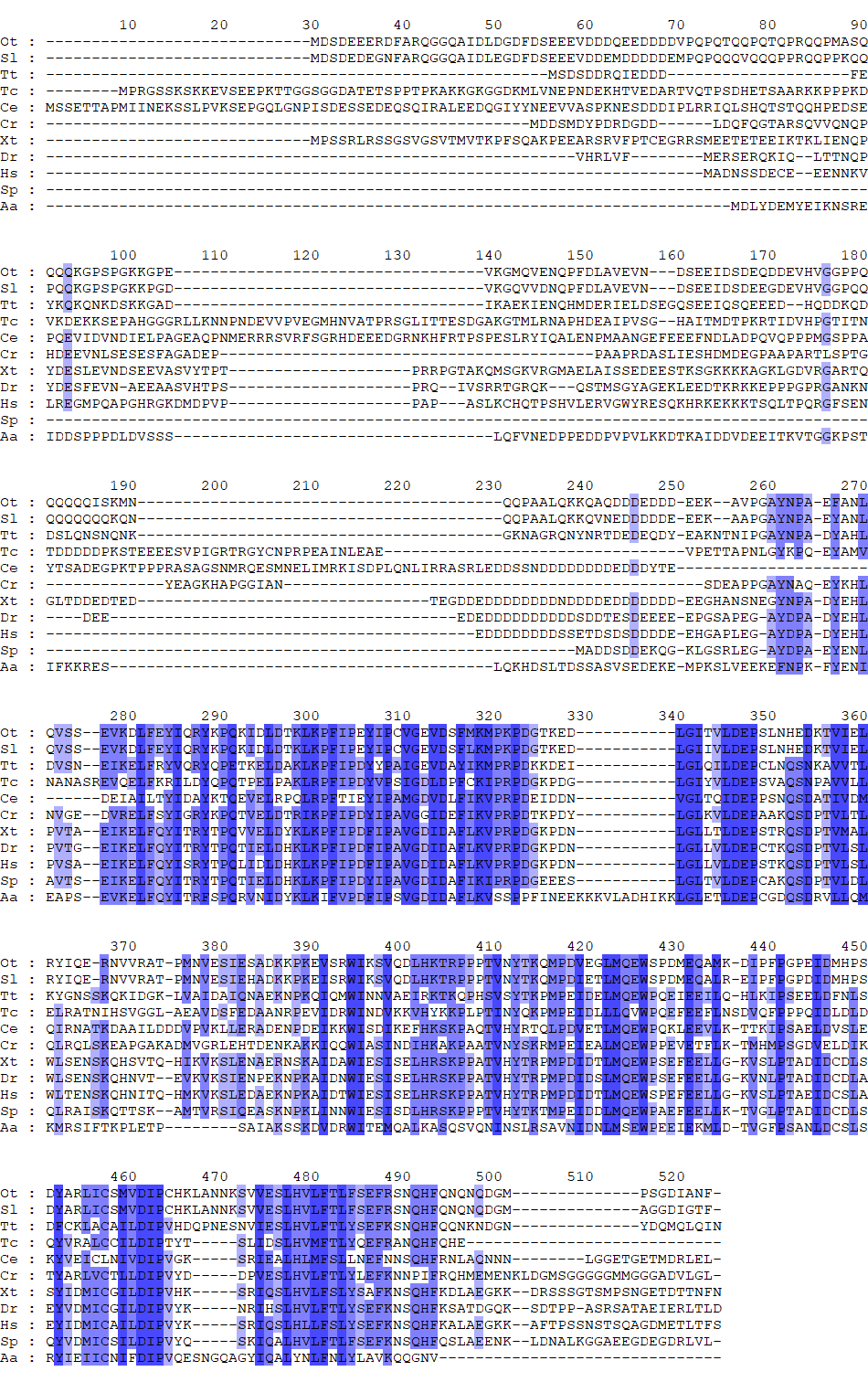


*Aedes aegypti* (Aa, EAT47766.1), *Caenorhabditis elegans* (Ce, NP_001076767.1), *Chlamydomonas reinhardtii* (Cr, A2T2X4.1), *Danio rerio* (Dr, AAI22333.1), *Homo sapiens* (Hs, NP_064538.3), *Oxytricha trifallax* (Ot, EJY85020.1), *Strongylocentrotus purpuratus* (Sp, XP_011663917.1), *Stylonychia lemnae* (Sl, CDW77990.1), *Tetrahymena thermophila* (Tt, TTHERM_00193580), *Trypanosoma cruzi strain CL Brener* (Tc, XP_845431.1), *Xenopus tropicalis* (Xt, XP_004916113.1)

**IFT52**


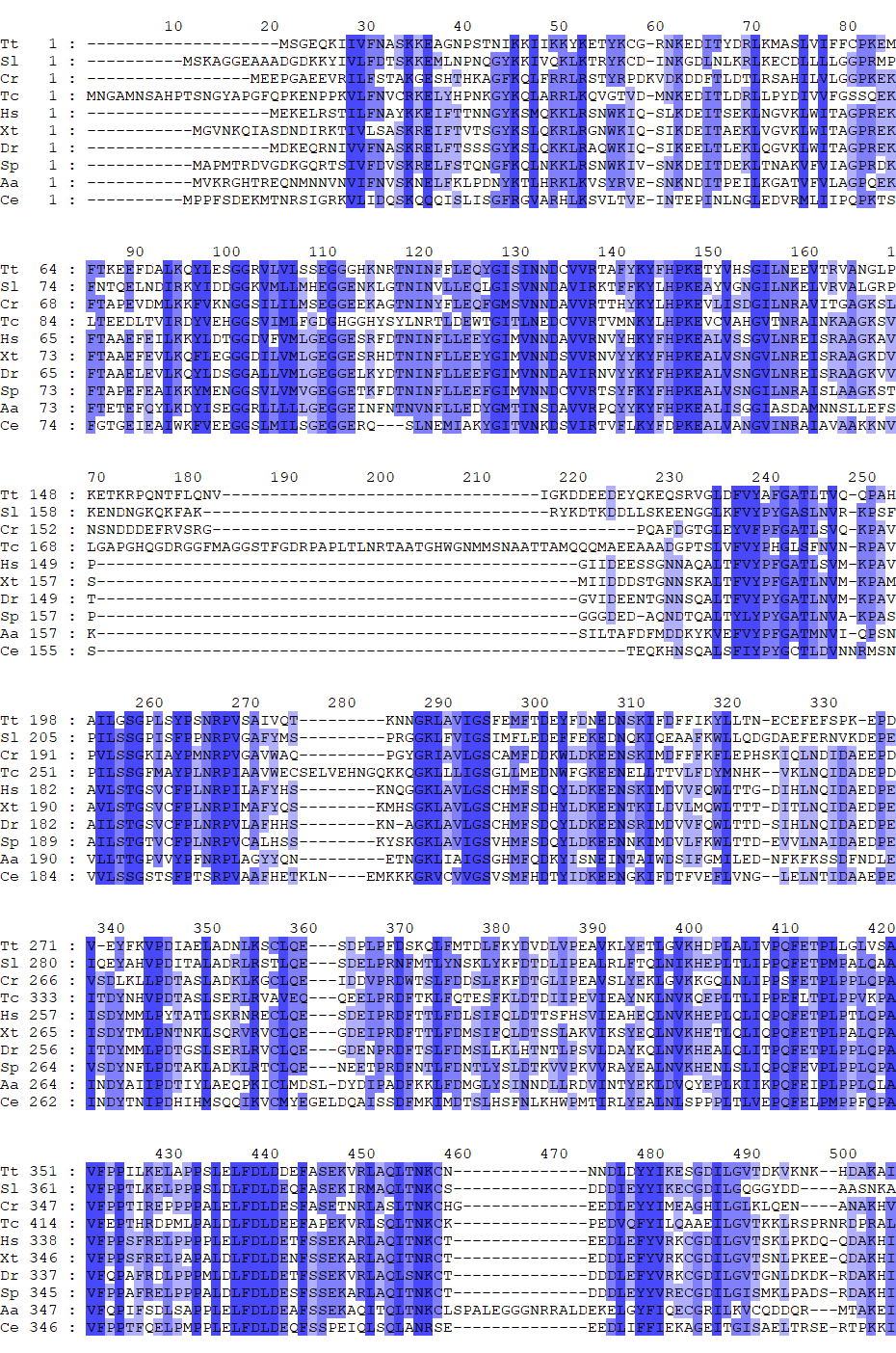


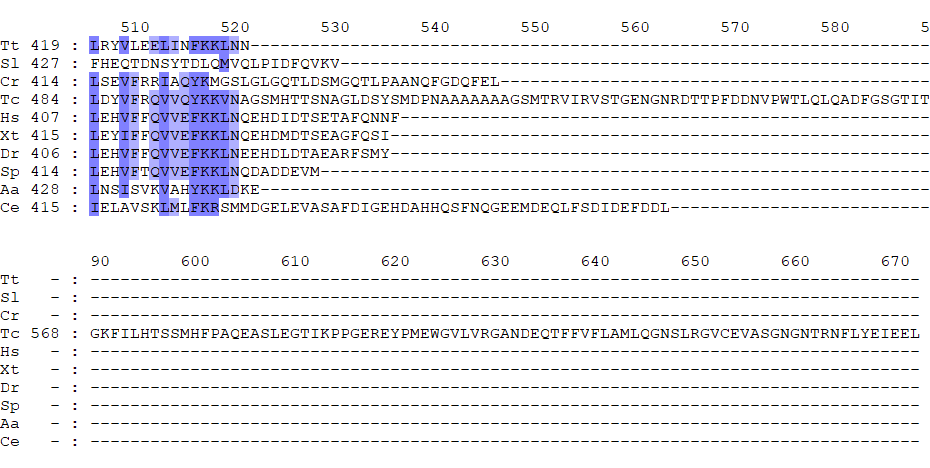


*Aedes albopictus* (Aa, XP_019535830.1), *Caenorhabditis elegans* (Ce, NP_741633.1), *Chlamydomonas reinhardtii* (Cr, AAL12162.1), *Danio rerio* (Dr, AAT27468.1), *Homo sapiens* (Hs, NP_057088.2), *Strongylocentrotus purpuratus* (Sp, XP_011667072.1), *Stylonychia lemnae* (Sl, CDW87019.1), *Tetrahymena thermophila* (Tt, TTHERM_00648910), *Trypanosoma cruzi strain CL Brener* (Tc, XP_812033.1), *Xenopus tropicalis* (Xt, AAI35679.1)

**IFT54**


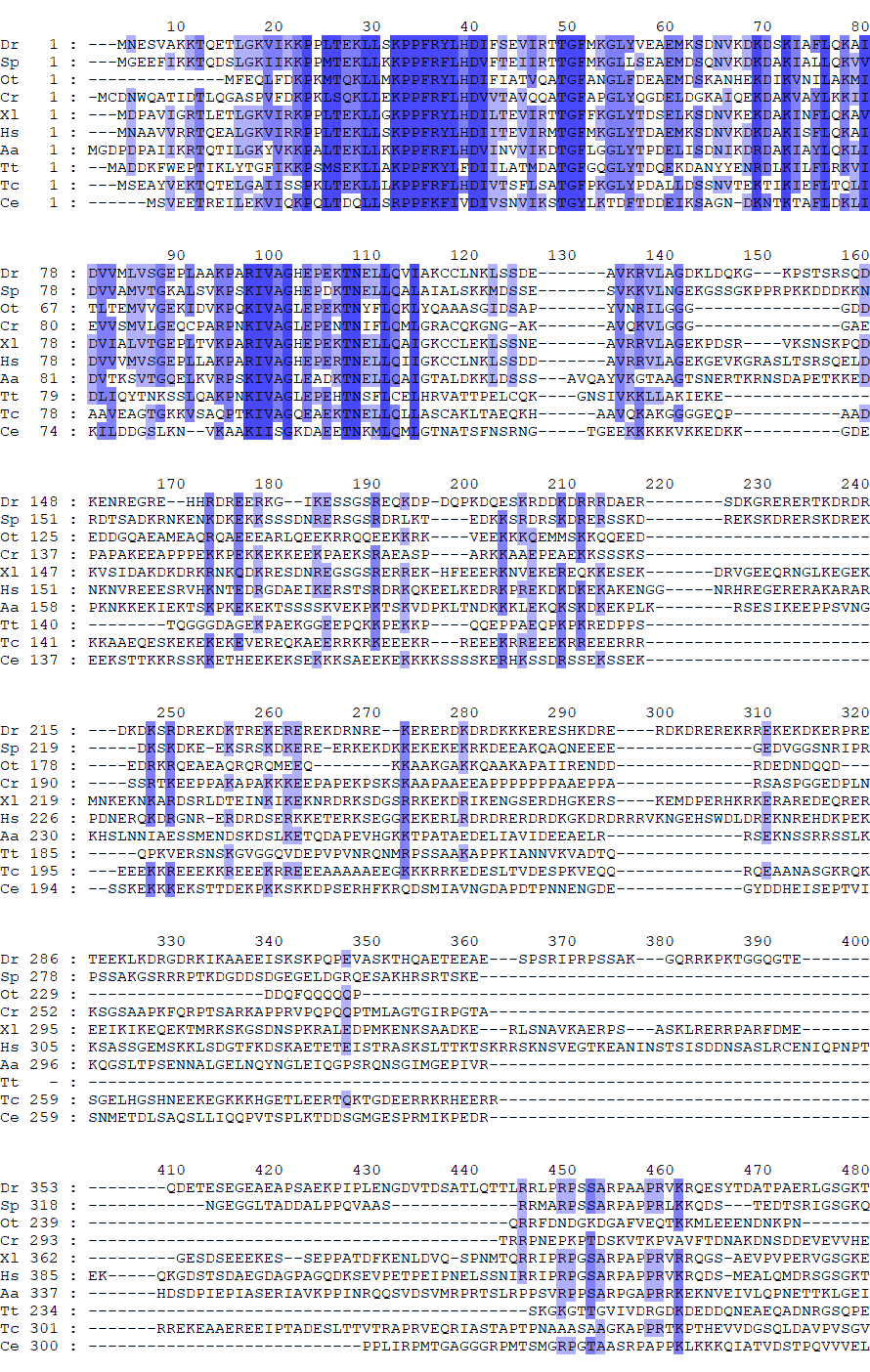


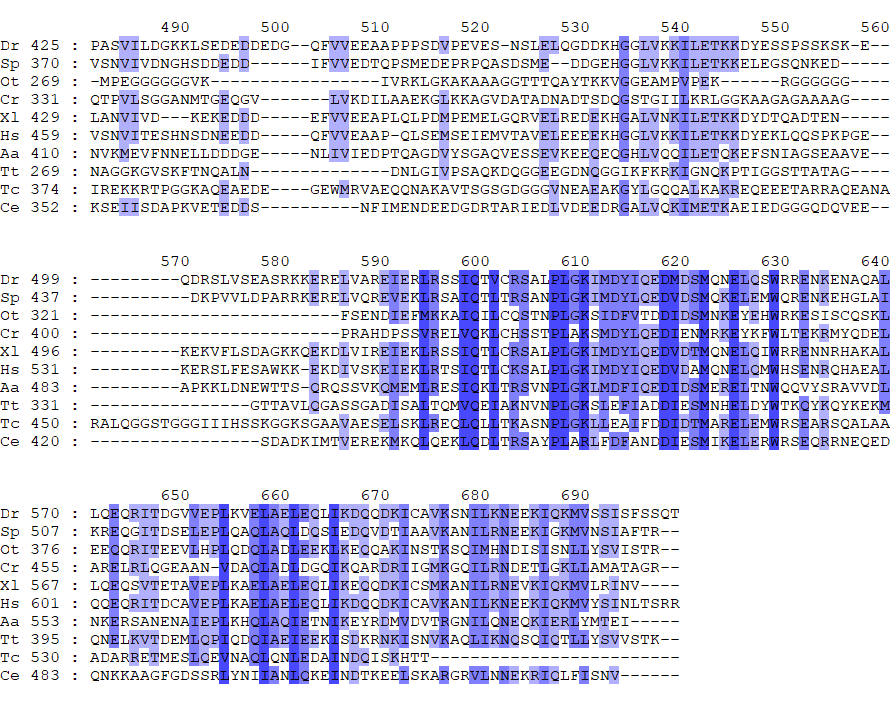


*Aedes albopictus* (Aa, XP_019565441.1), *Caenorhabditis elegans* (Ce, NP_508149.1), *Chlamydomonas reinhardtii* (Cr, XP_001699472.1), *Danio rerio* (Dr, XP_005172584.1), *Homo sapiens* (Hs, XP_011509250.1), *Oxytricha trifallax* (Ot, EJY72177.1), *Strongylocentrotus purpuratus* (Sp, XP_011674772.1), *Tetrahymena thermophila* (Tt, TTHERM_01070330), *Trypanosoma cruzi strain CL Brener* (Tc, XP_812718.1), *Xenopus laevis* (Xl, XP_018094405.1)

Red frame indicates the position of the ciliopathy-causing mutations: Hs I17S corresponding to Tt I17S).

**IFT81**


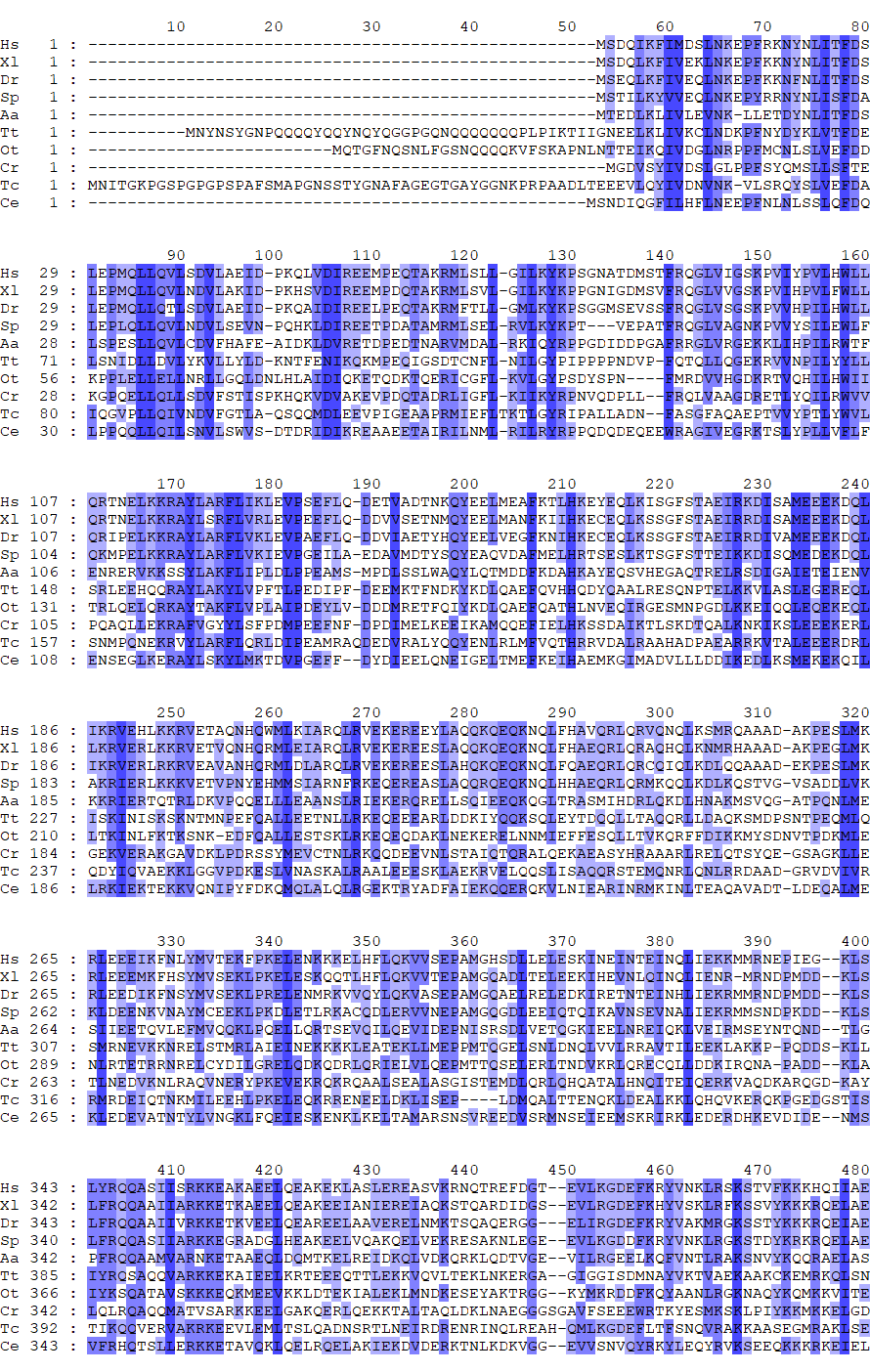


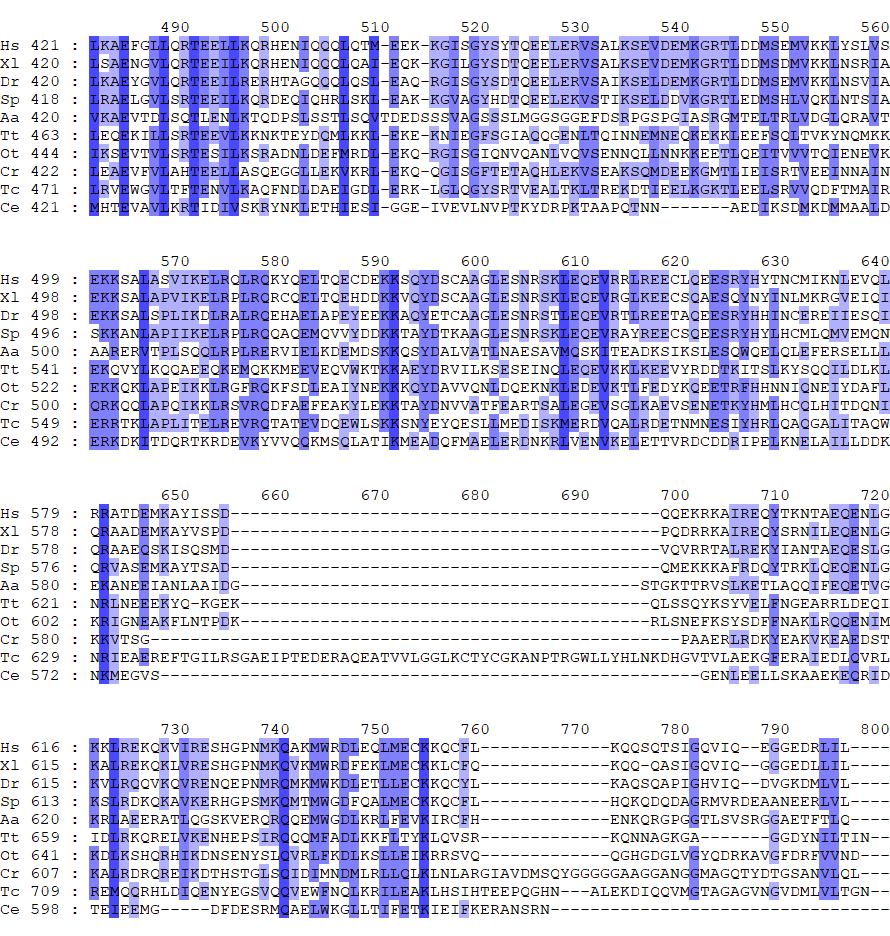


*Aedes albopictus* (Aa, XP_019544729.2), *Caenorhabditis elegans* (Ce, NP_508900.6), *Chlamydomonas reinhardtii* (Cr, XP_001697224.1), *Danio rerio* (Dr, AAI63882.1), *Homo sapiens* (Hs, NP_001137251.1), *Oxytricha trifallax* (Ot, EJY69053.1), *Strongylocentrotus purpuratus* (Sp, XP_011667377.1), *Tetrahymena thermophila* (Tt, TTHERM_01013160), *Trypanosoma cruzi strain CL Brener* (Tc, XP_812212.1), *Xenopus laevis* (Xl, XP_018118823.1)

**IFT122**


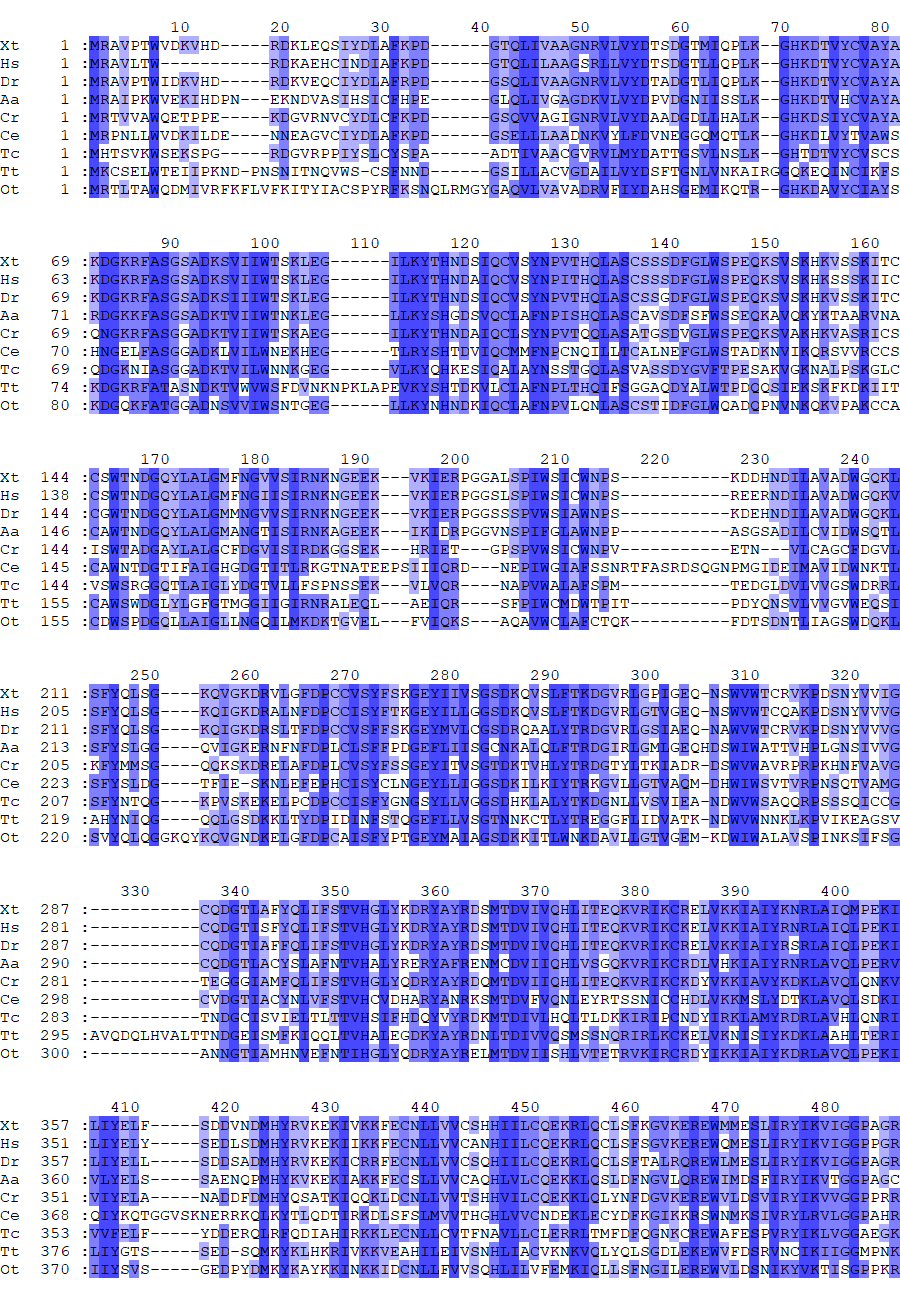


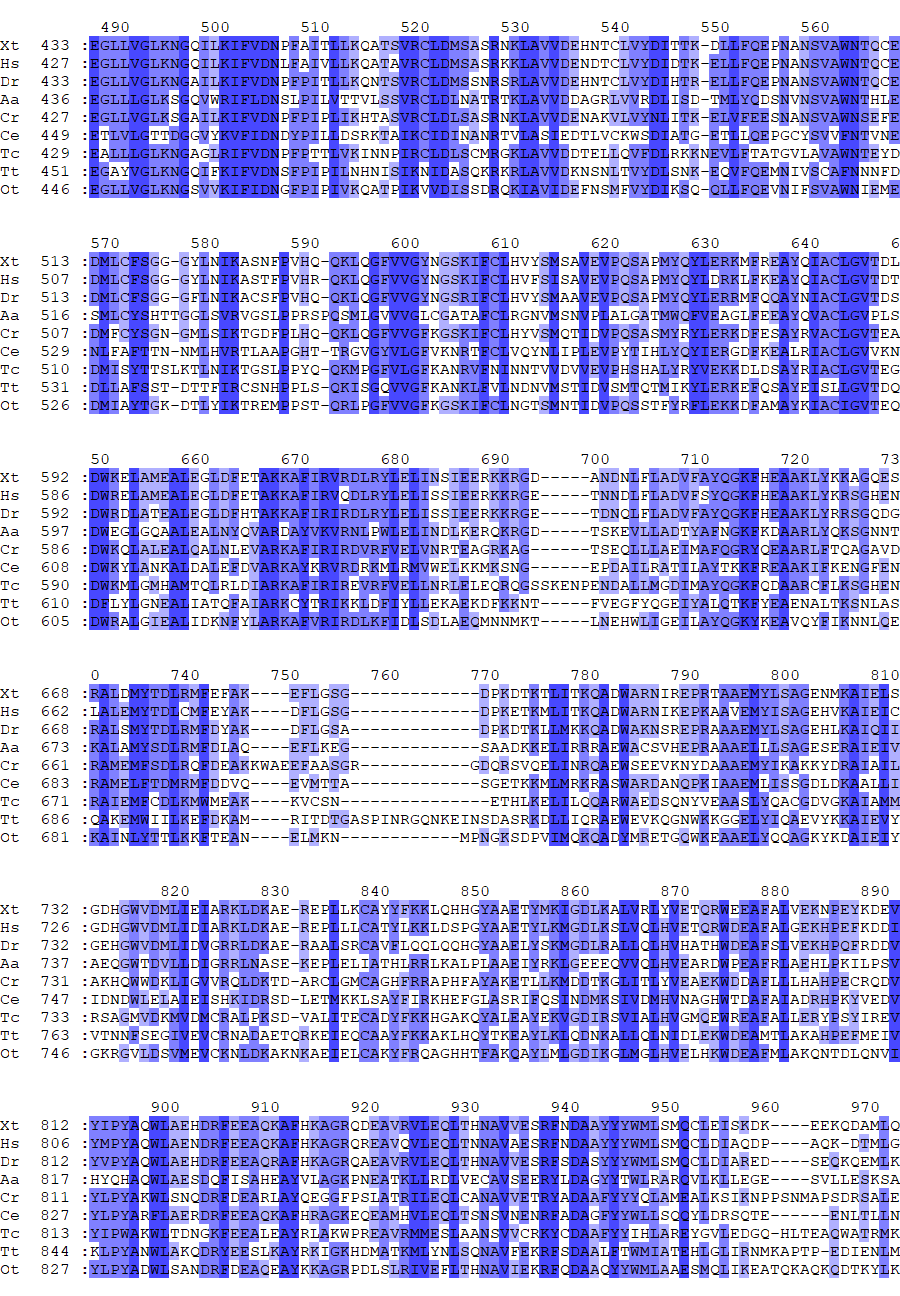


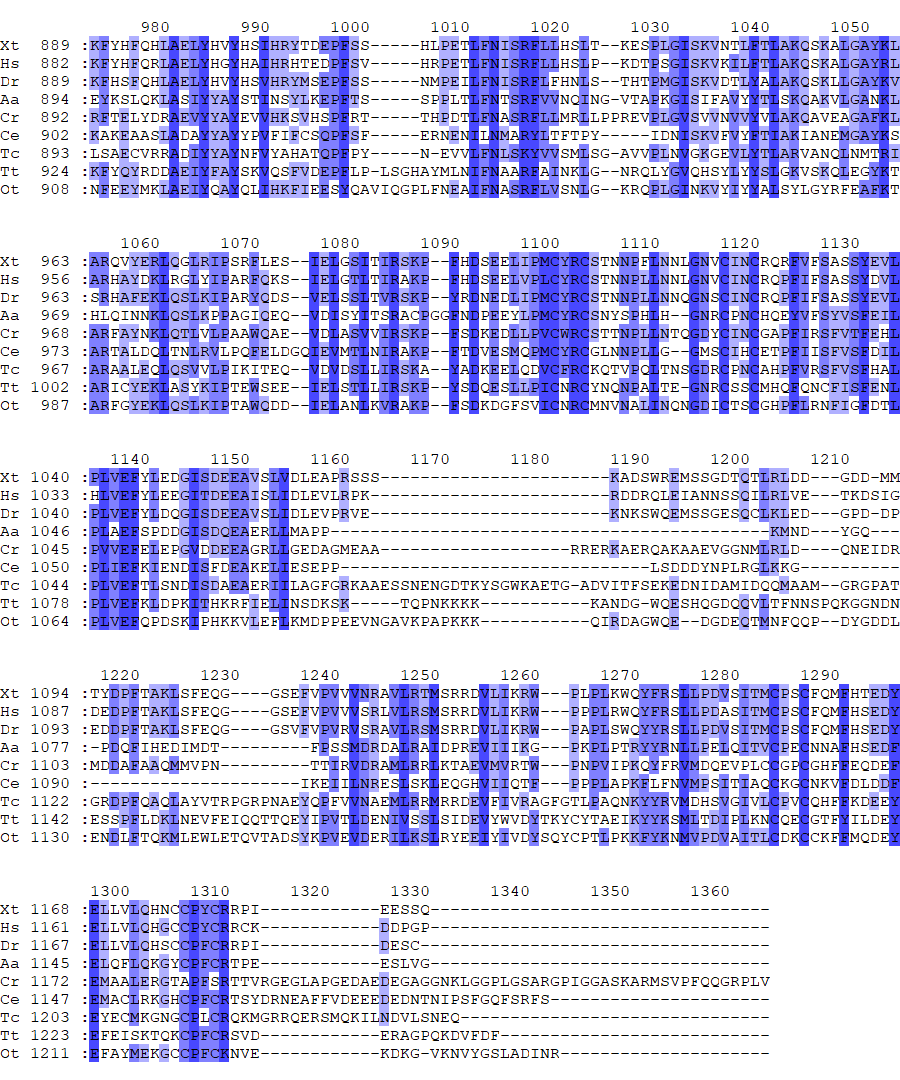


*Aedes albopictus* (Aa, XP_019531362.1), *Caenorhabditis elegans* (Ce, NP_501591.2), *Chlamydomonas reinhardtii* (Cr, AFC88886.1), *Danio rerio* (Dr, NP_998055.1), *Homo sapiens* (Hs, NP_060732.2), *Oxytricha trifallax* (Ot, EJY69814.1), *Tetrahymena thermophila* (Tt, TTHERM_00694540), *Trypanosoma cruzi strain CL Brener* (Tc, XP_820043.1), *Xenopus tropicalis* (Xt, NP_001106492.1)

**IFT140**


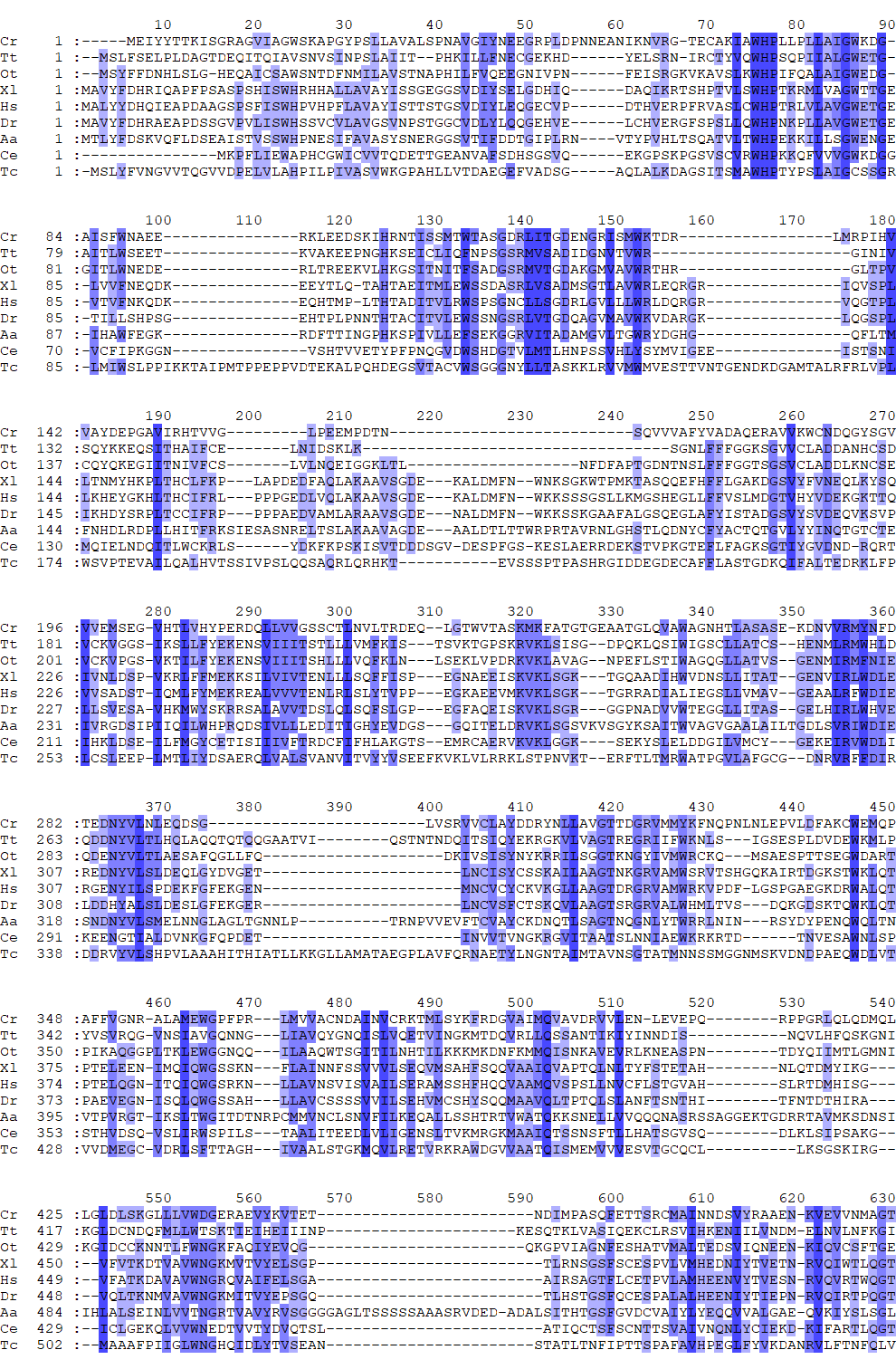


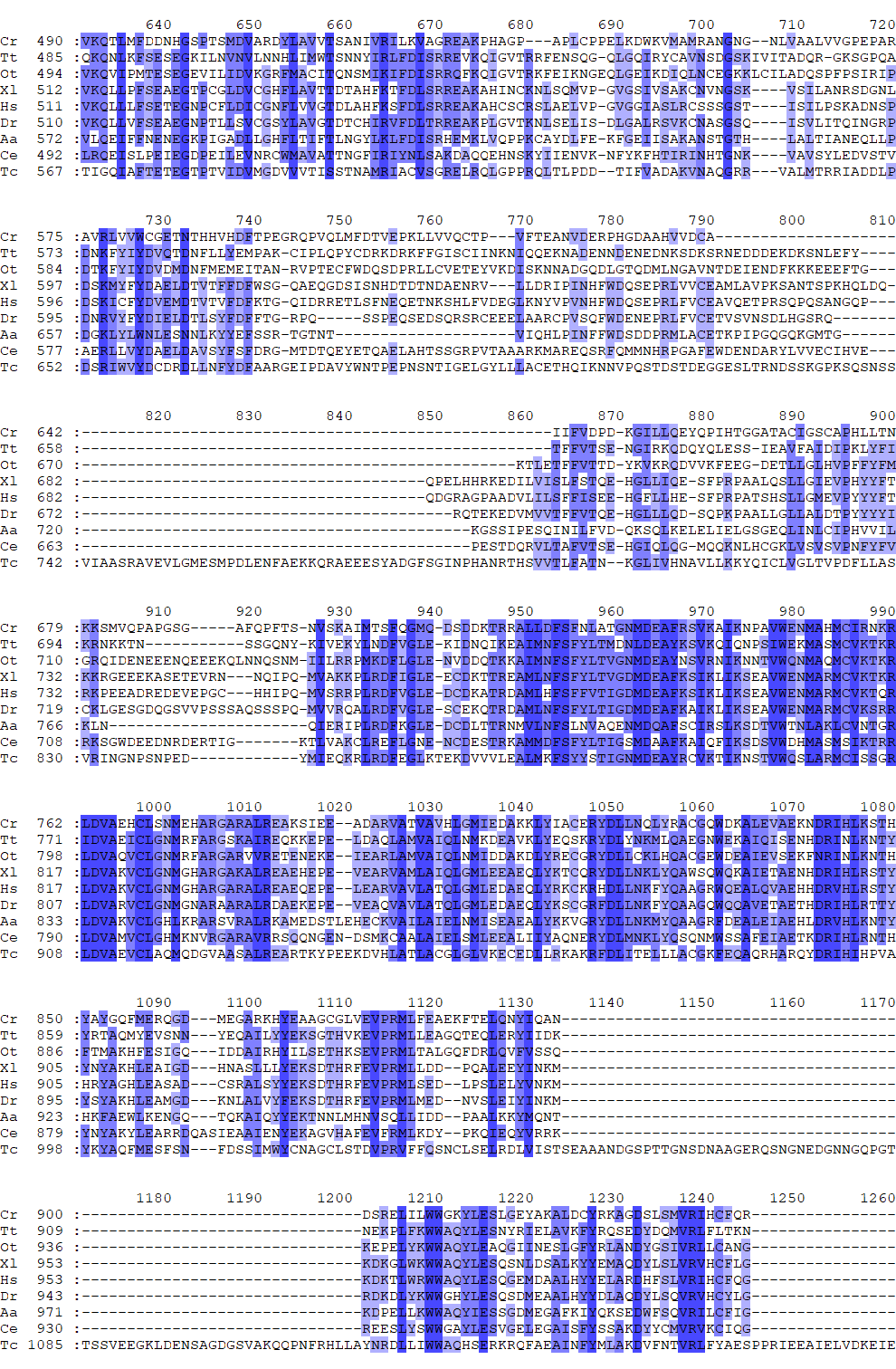


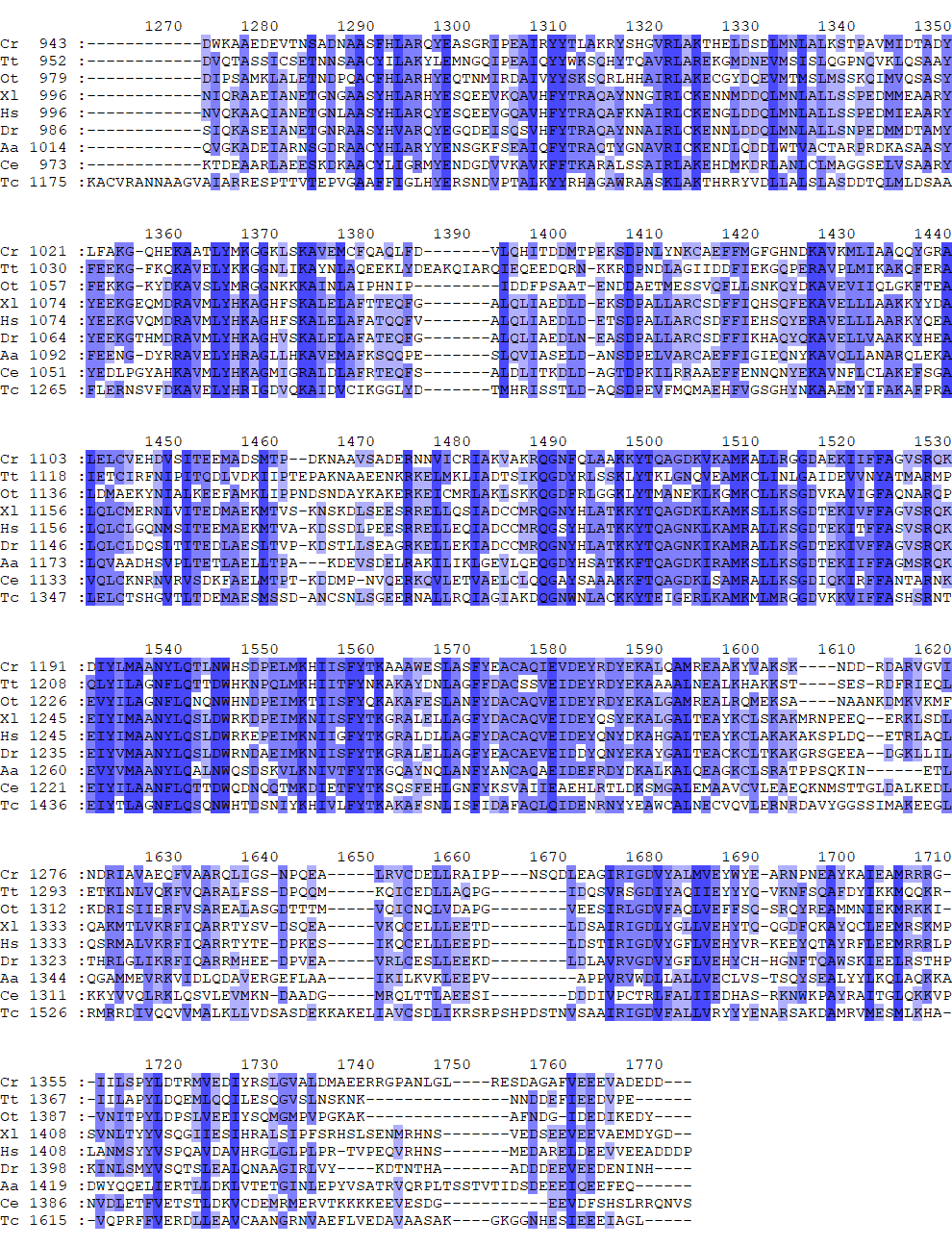


*Aedes albopictus* (Aa, KXJ70504.1), *Caenorhabditis elegans* (Ce, NP_506047.5), *Chlamydomonas reinhardtii* (Cr, PNW79678.1), *Danio rerio* (Dr, XP_695732.4), *Homo sapiens* (Hs, NP_055529.2), *Oxytricha trifallax* (Ot, EJY86706.1), *Tetrahymena thermophila* (Tt, TTHERM_00220810), *Trypanosoma cruzi marinkellei* (Tc, EKF31357) *Xenopus laevis* (Xl, XP_018091986.1)

**IFT144**


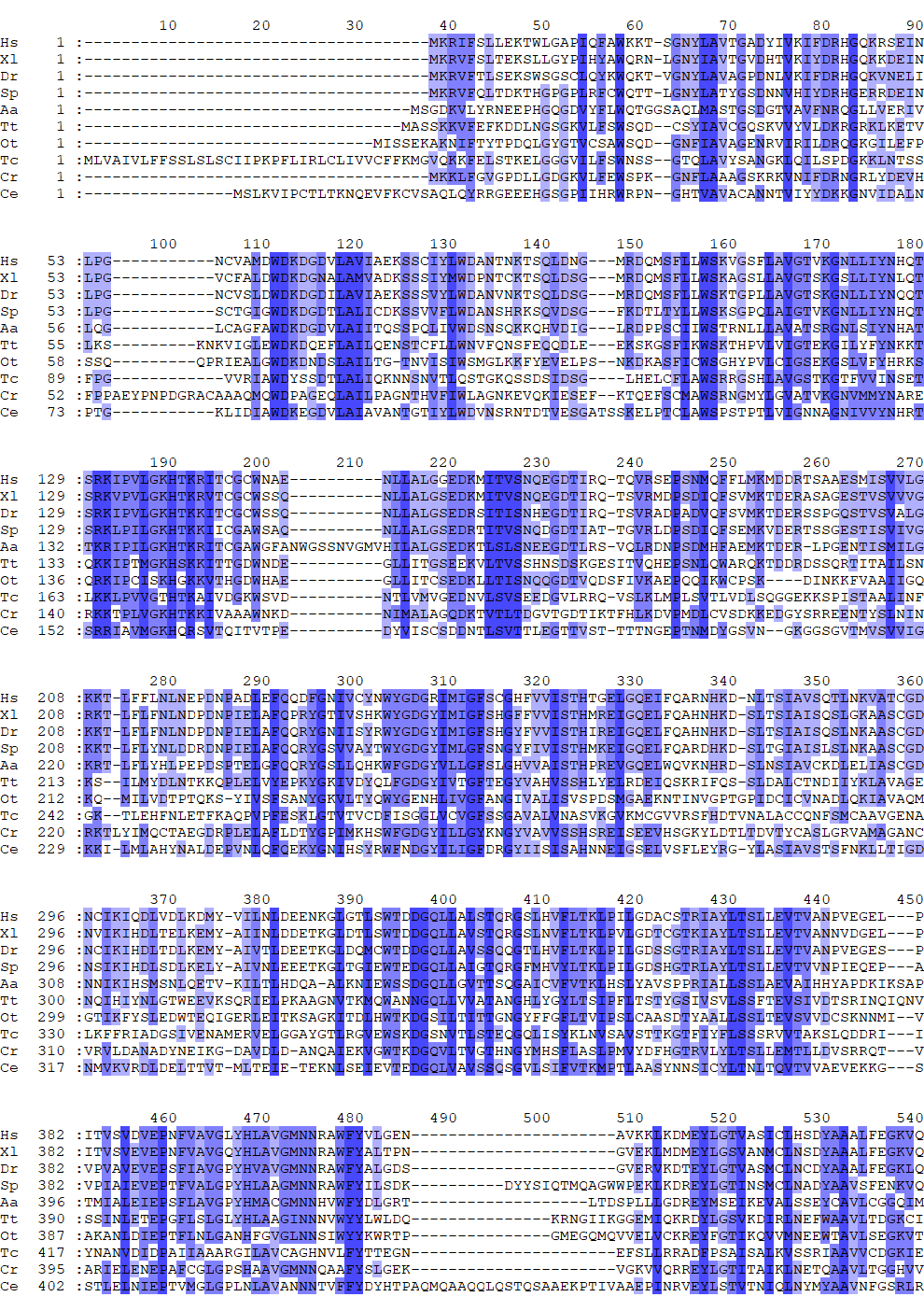


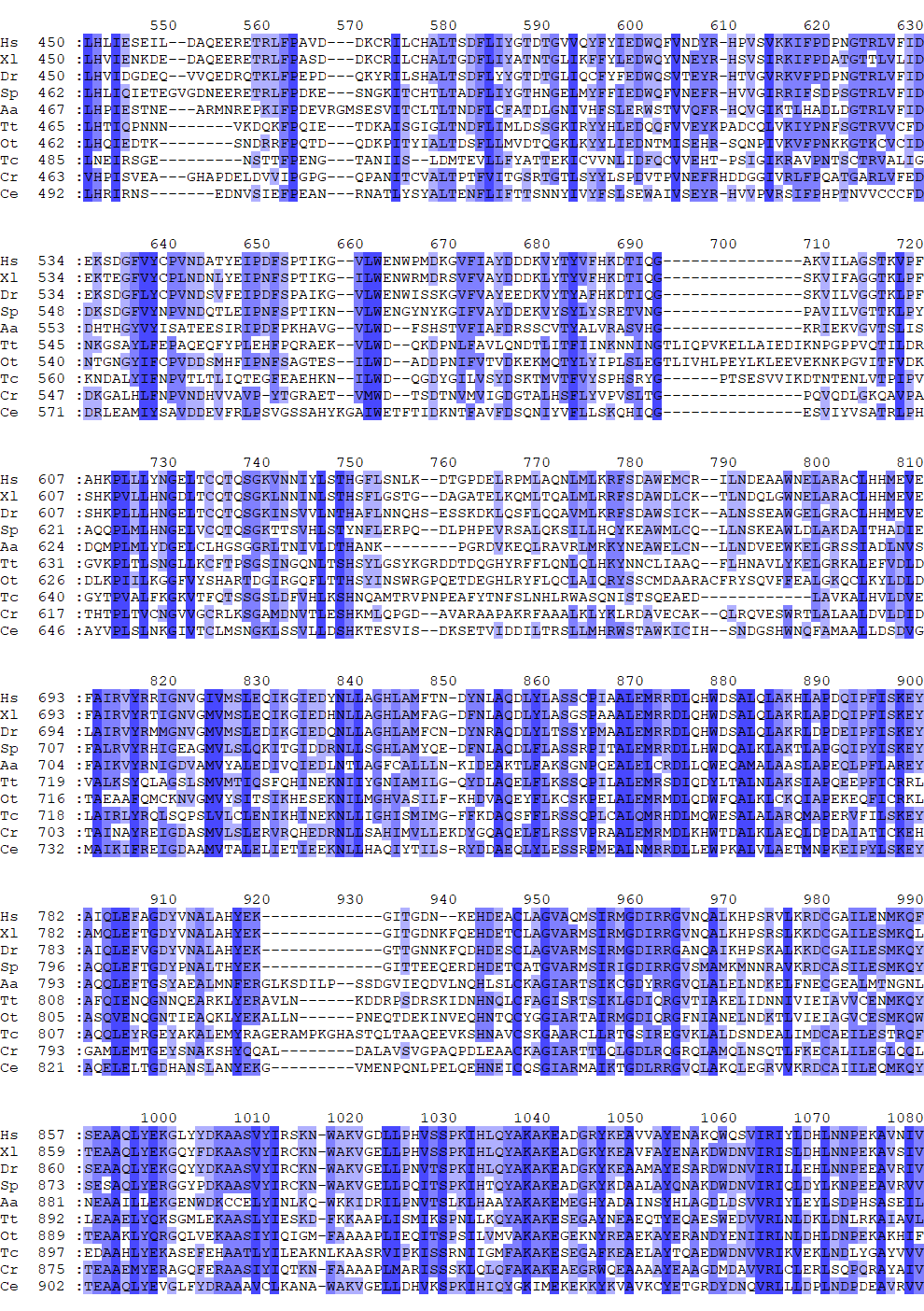


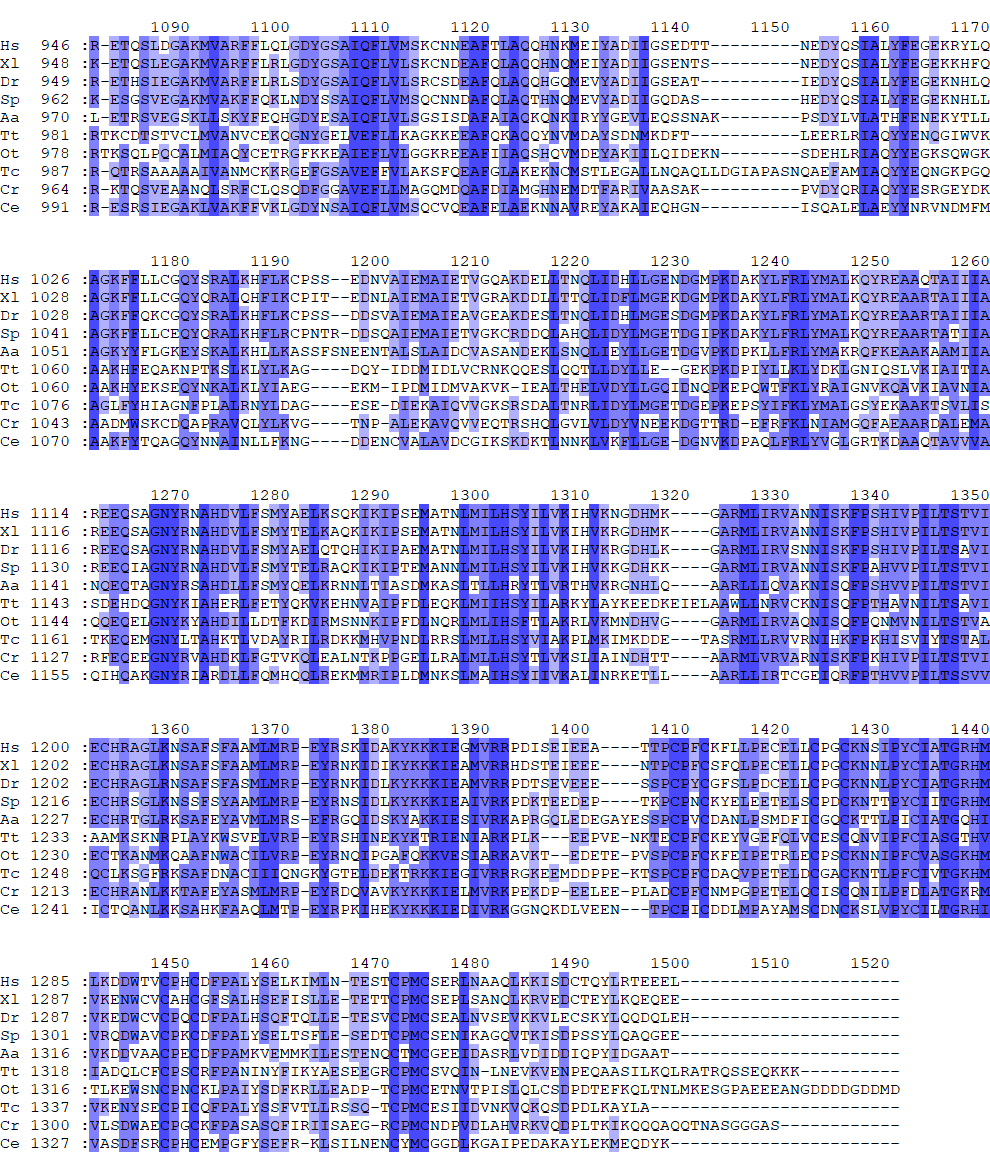


*Aedes albopictus* (Aa, XP_019530316.1), *Caenorhabditis elegans* (Ce, NP_001076655.1), *Chlamydomonas reinhardtii* (Cr, ABU95019.1), *Danio rerio* (Dr, XP_021326861.1), *Homo sapiens* (Hs, AAK38745.1), *Oxytricha trifallax* (Ot, EJY64992.1), *Strongylocentrotus purpuratus* (Sp, XP_011664189.1), *Tetrahymena thermophila* (Tt, TTHERM_01093530), *Trypanosoma cruzi Dm28c* (Tc, ESS69283.1) *Xenopus laevis* (Xl, XP_018084648.1)

**ODA16**


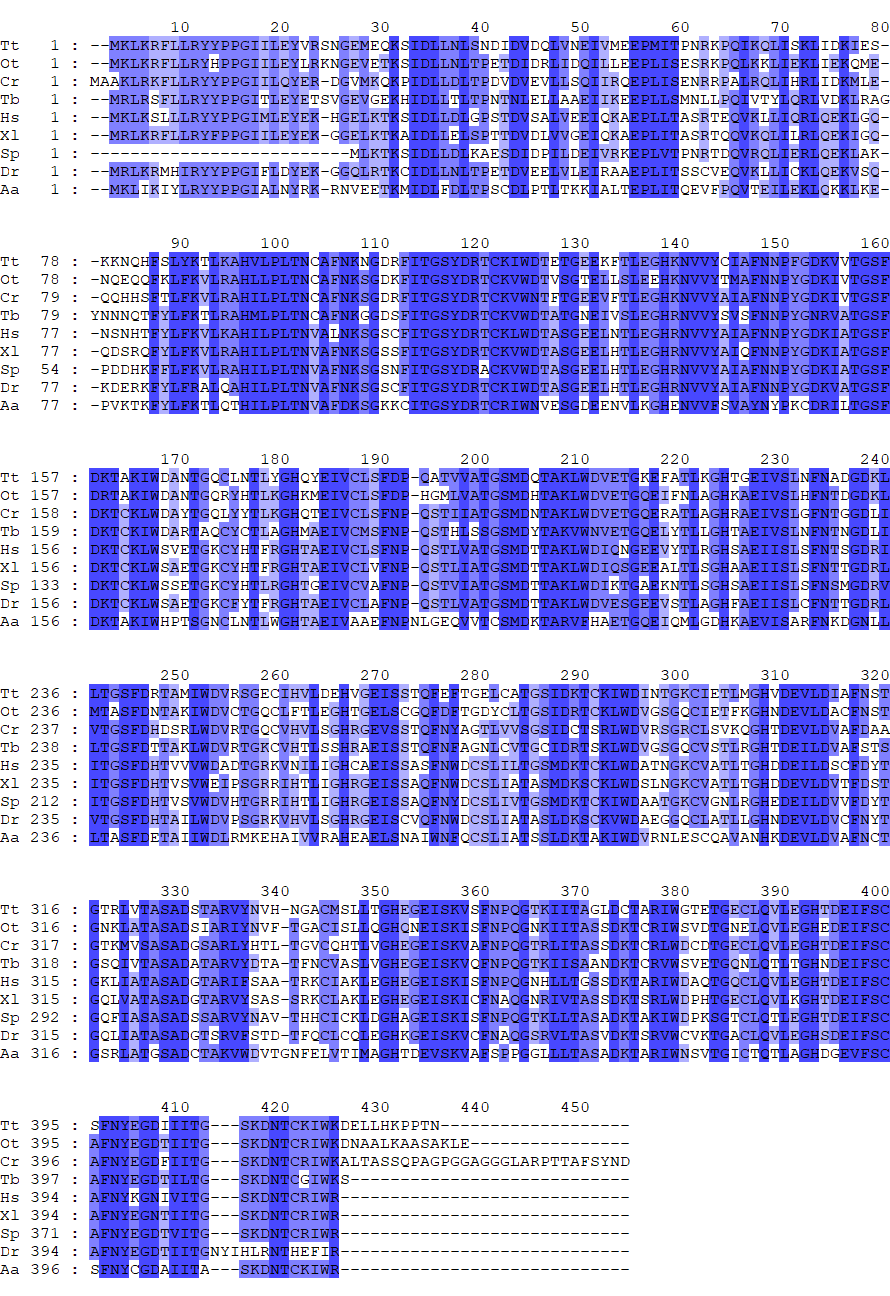


*Aedes aegypti* (Aa, XP_001650817.2), *Chlamydomonas reinhardtii* (Cr, AAZ77789.1), *Danio rerio* (Dr, NP_001037817.1), *Homo sapiens* (Hs, NP_849143.1), *Oxytricha trifallax* (Ot, EJY64778.1), *Strongylocentrotus purpuratus* (Sp, XP_011664431.1), *Tetrahymena thermophila* (Tt, TTHERM_00762930), *Trypanosoma brucei brucei TREU927* (Tb, XP_847252.1), *Xenopus laevis* (Xl, NP_001089233.

**OFD1**


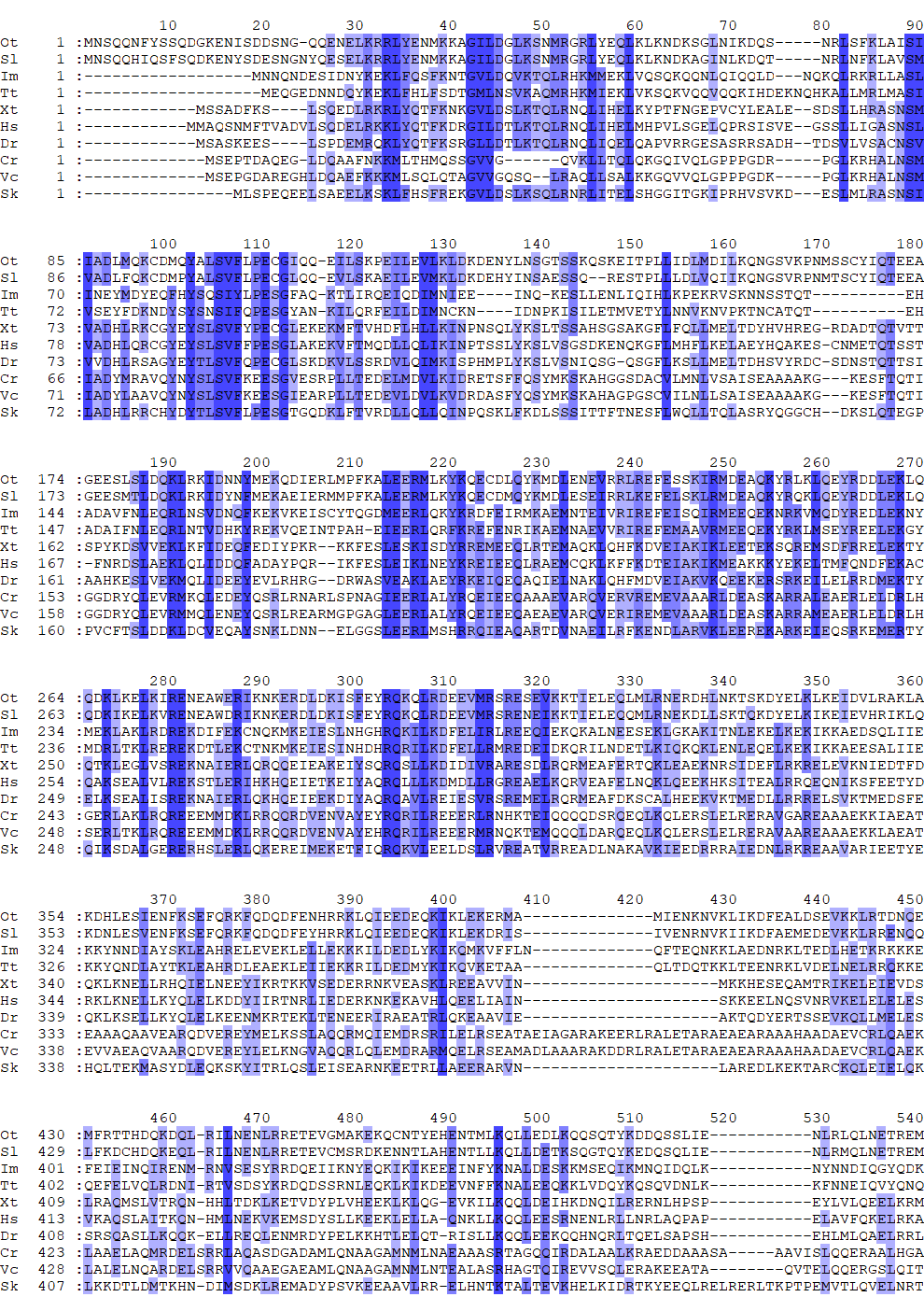


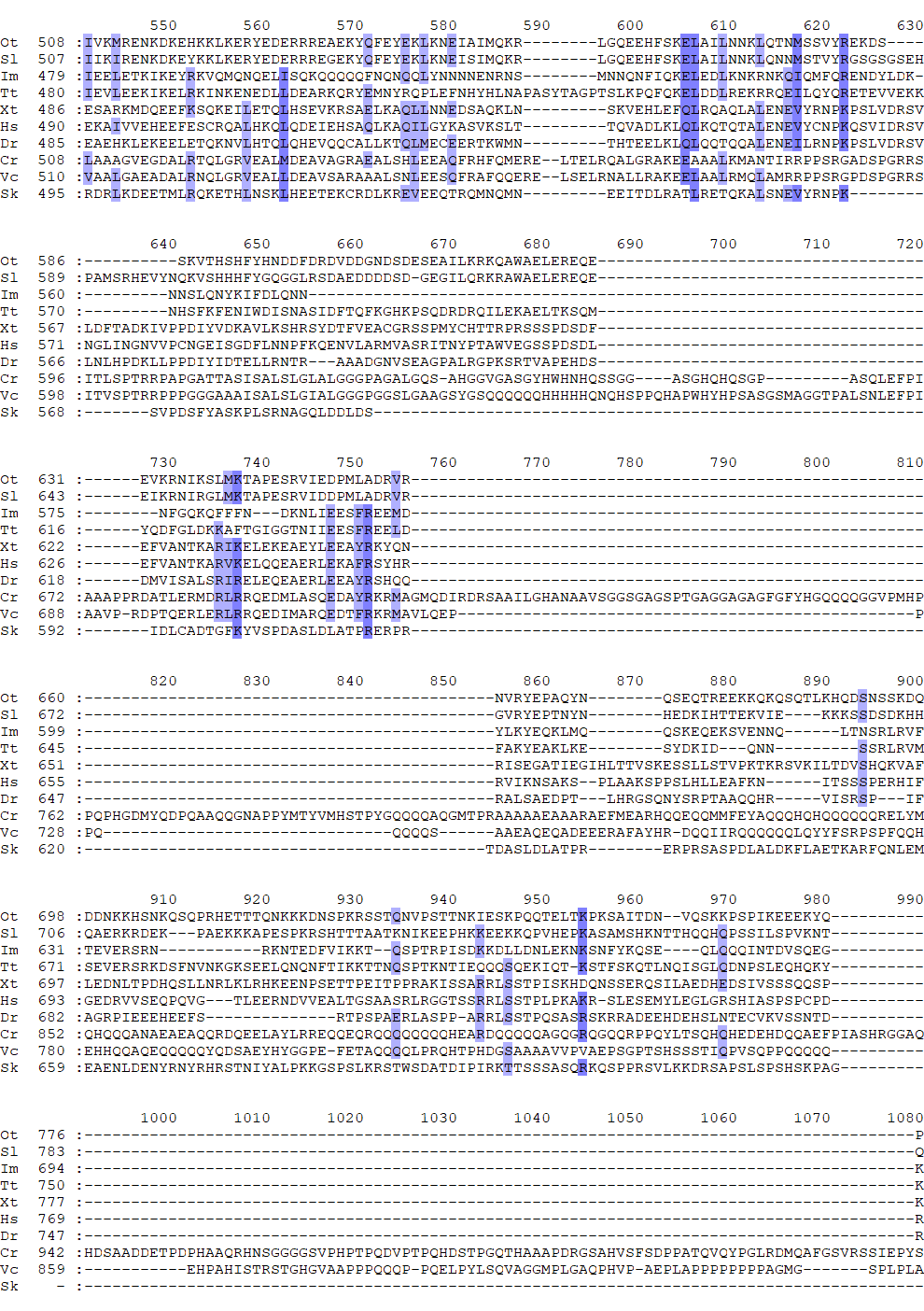


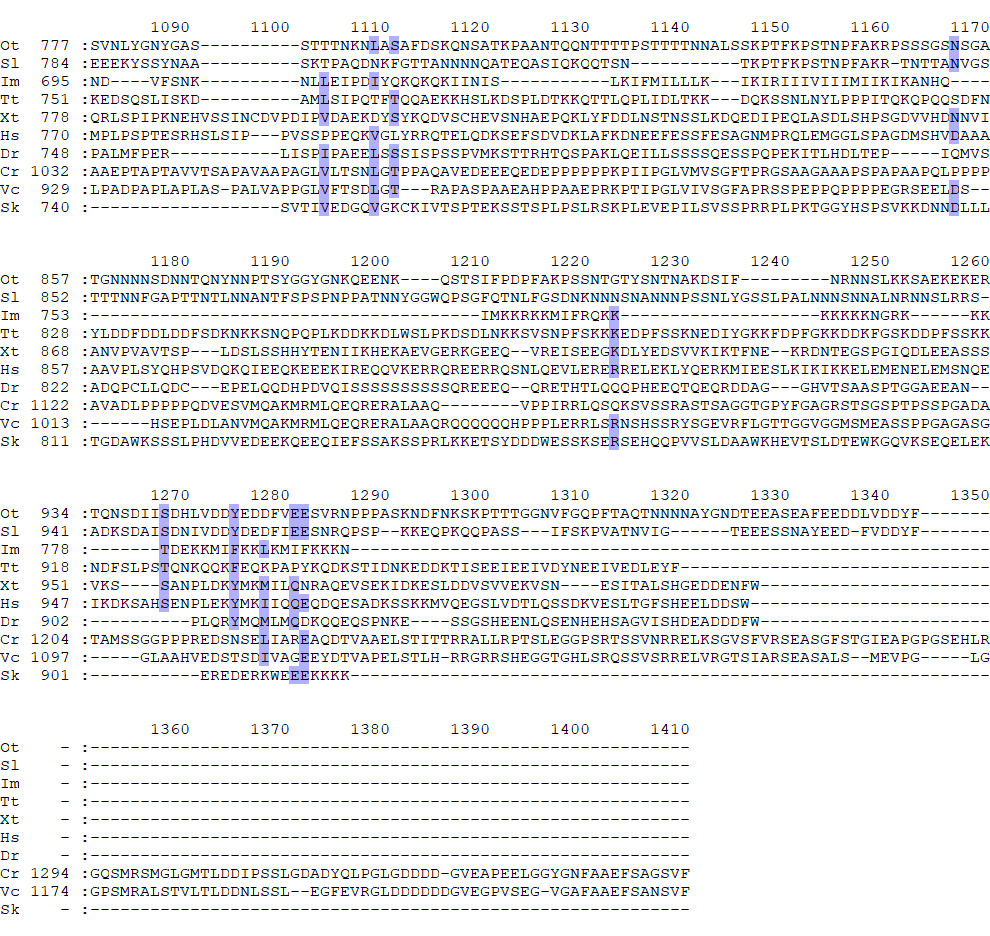


*Chlamydomonas reinhardtii* (Cr, XP_001691583.1), *Danio rerio* (Dr, XP_009303289.1), *Homo sapiens* (Hs, NP_003602.1), *Ichthyophthirius multifiliis* (Im, XP_004027357.1), *Oxytricha trifallax* (Ot, EJY64486.1), *Saccoglossus kowalevskii* (Sk, ADB22615.1 ), *Stylonychia lemnae (*Sl*,* CDW79034.1), *Tetrahymena thermophila* (Tt, TTHERM_00214750), *Volvox carteri f. nagariensis* (Vc, XP_002949660.1 ), *Xenopus tropicalis* (Xt, XP_012813078.1)

**BBS1**


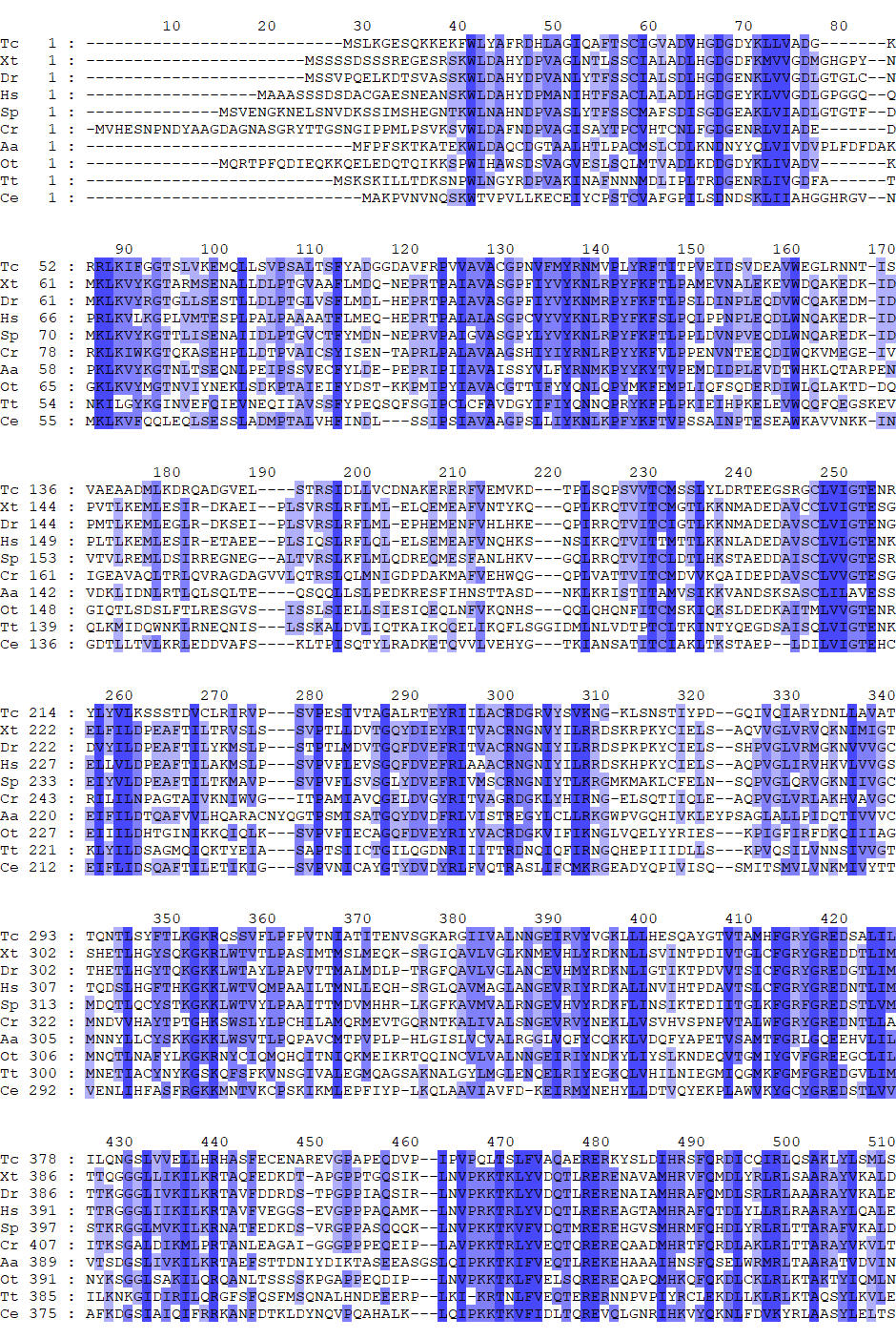


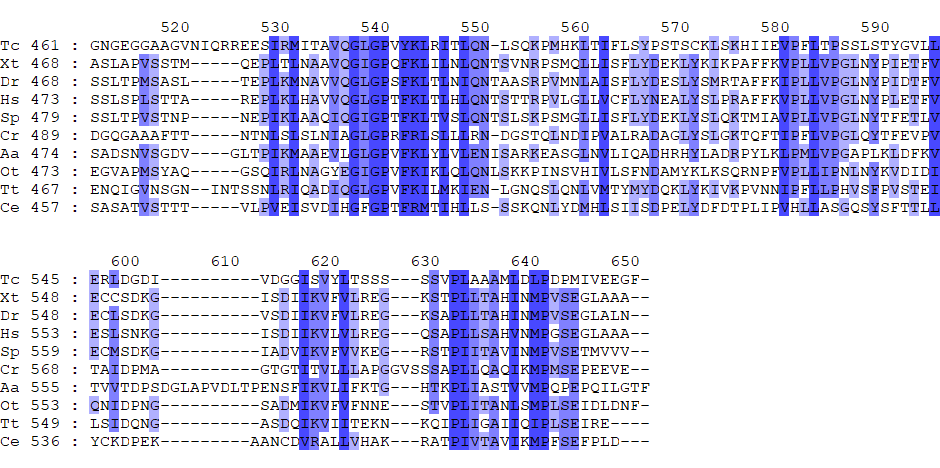


*Aedes aegypti* (Aa, XP_001659822.1), *Caenorhabditis elegans* (Ce, NP_740933.1), *Chlamydomonas reinhardtii* (Cr, XP_001701093.1), *Danio rerio* (Dr, AAI62155.1), *Homo sapiens* (Hs, NP_078925.3), *Oxytricha trifallax* (Ot, EJY73419.1), *Strongylocentrotus purpuratus* (Sp, XP_011666764.1), *Tetrahymena thermophila* (Tt, TTHERM_01084190), *Trypanosoma cruzi strain CL Brener* (Tc, XP_821605.1), *Xenopus tropicalis* (Xt, XP_012815951.1)

**BBS5**


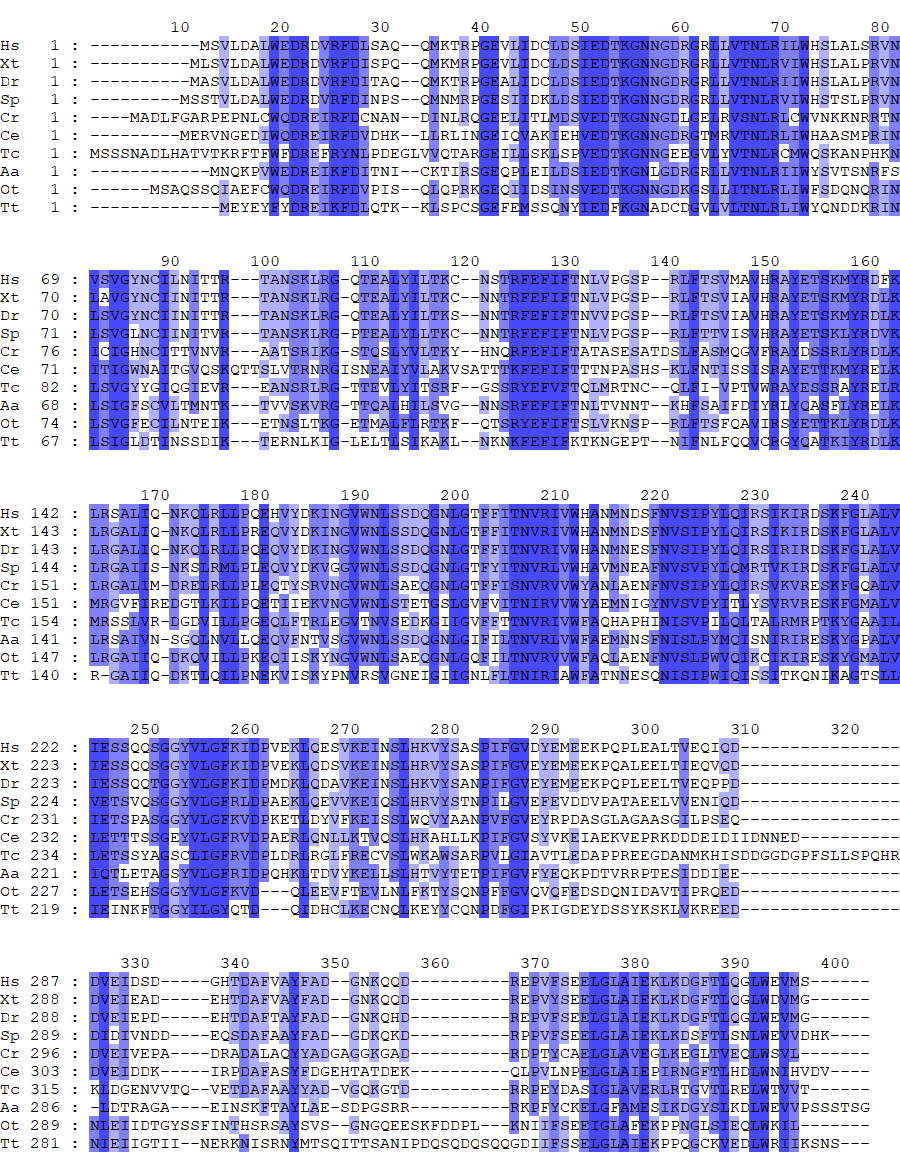


*Aedes aegypti* (Aa, XP_001653052.1), *Caenorhabditis elegans* (Ce, NP_499272.1), *Chlamydomonas reinhardtii* (Cr, AAS89977.1), *Danio rerio* (Dr, NP_956593.1), *Homo sapiens* (Hs, AAH44593.1), *Oxytricha trifallax* (Ot, EJY66062.1), *Strongylocentrotus purpuratus* (Sp, XP_011680639.1), *Tetrahymena thermophila* (Tt, TTHERM_00782070), *Trypanosoma cruzi strain CL Brener* (Tc, XP_820870.1), *Xenopus tropicalis* (Xt, NP_001016238.1). Red frame indicates the position of the ciliopathy-causing mutations: Hs A323CfsXand Tt A332Cfs.

**BBS9**


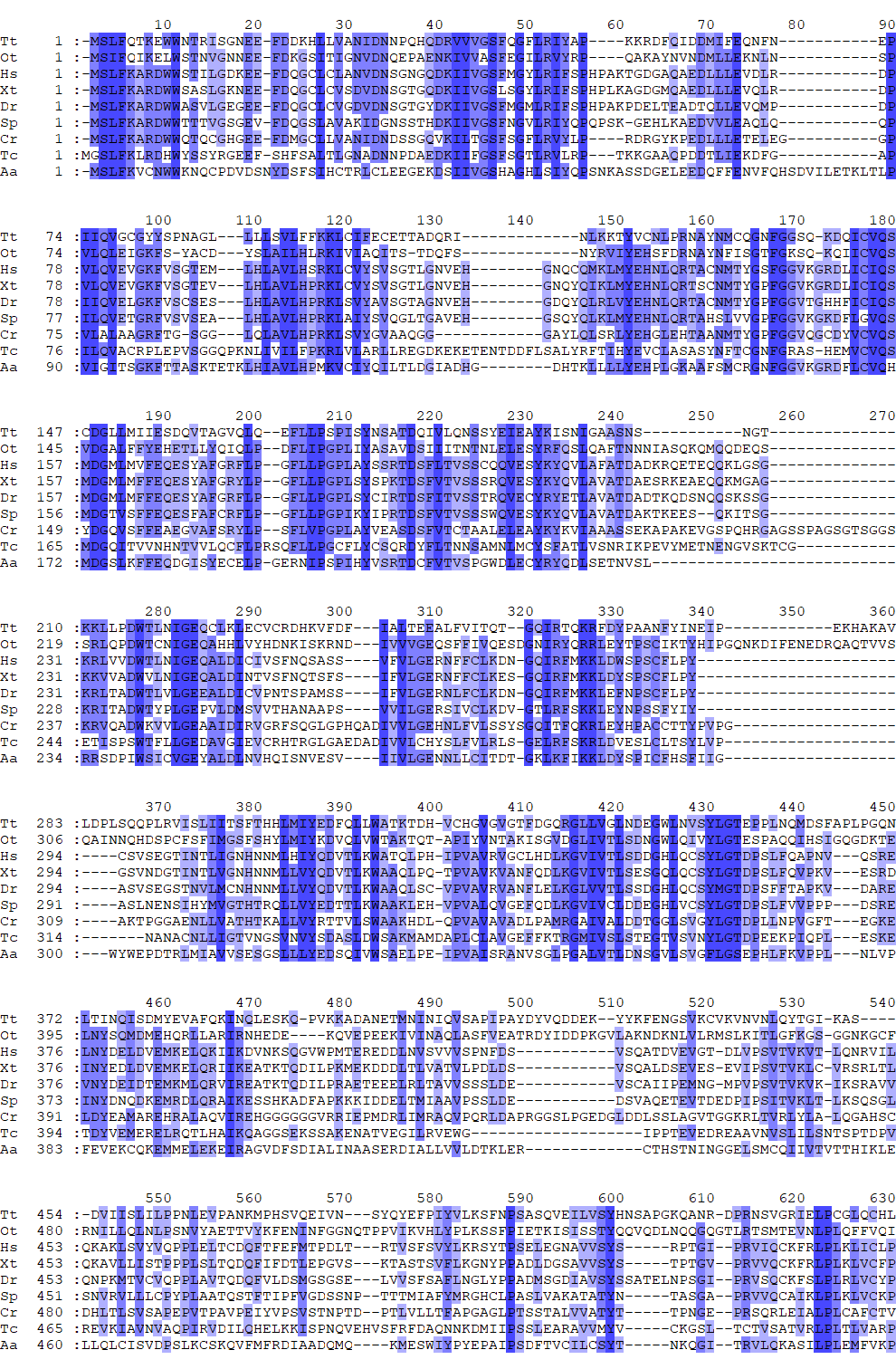


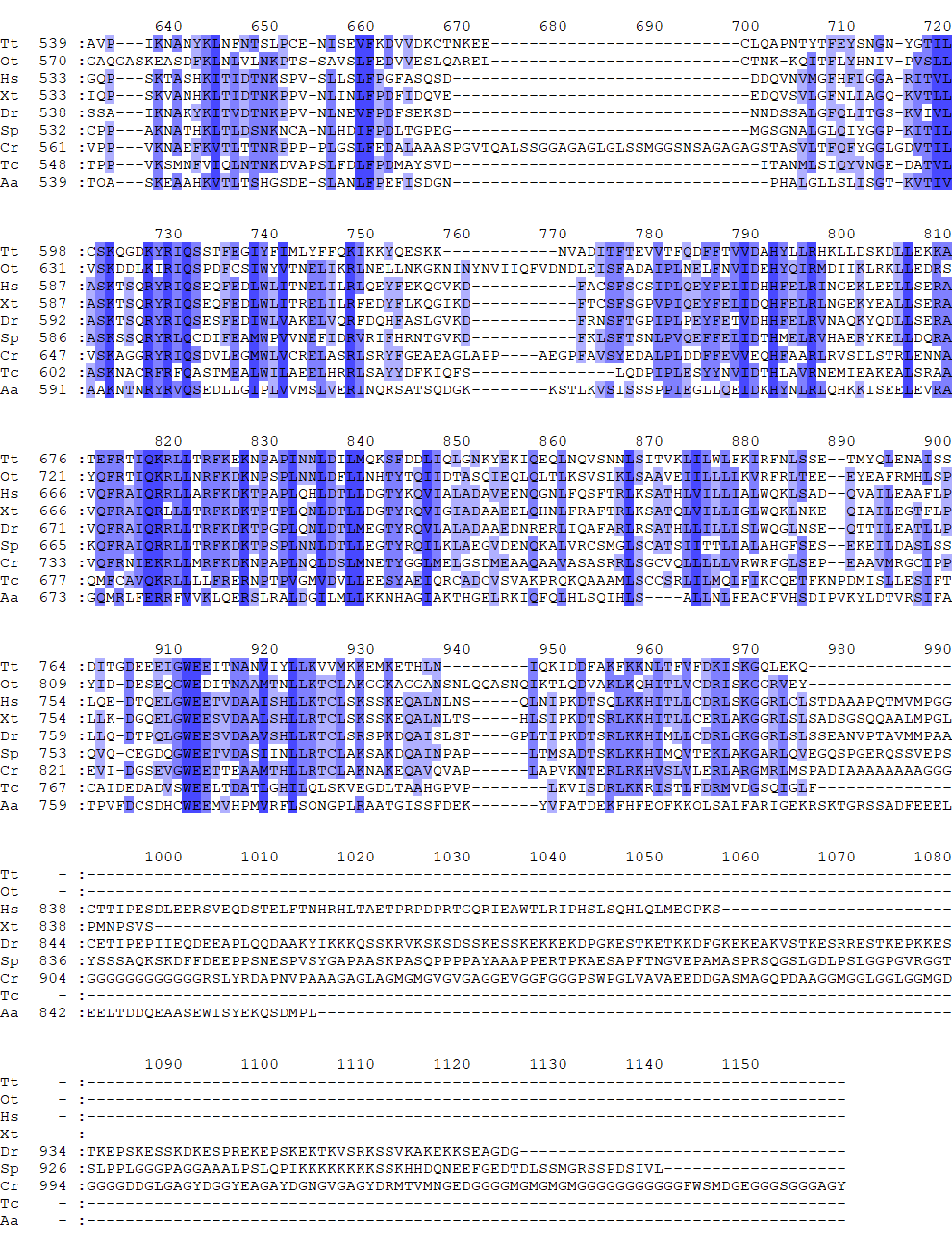


*Aedes albopictus* (Aa, XP_019554287.1), *Chlamydomonas reinhardtii* (Cr, PNW84061.1), *Danio rerio* (Dr, XP_009290332.2), *Homo sapiens* (Hs, XP_016867479.1), *Oxytricha trifallax* (Ot, EJY72742.1), *Strongylocentrotus purpuratus* (Sp, XP_791924.1), *Tetrahymena thermophila* (Tt, TTHERM_00518740), *Trypanosoma cruzi strain CL Brener* (Tc, XP_815952.1), *Xenopus tropicalis* (Xt, NP_001016821.1)

**
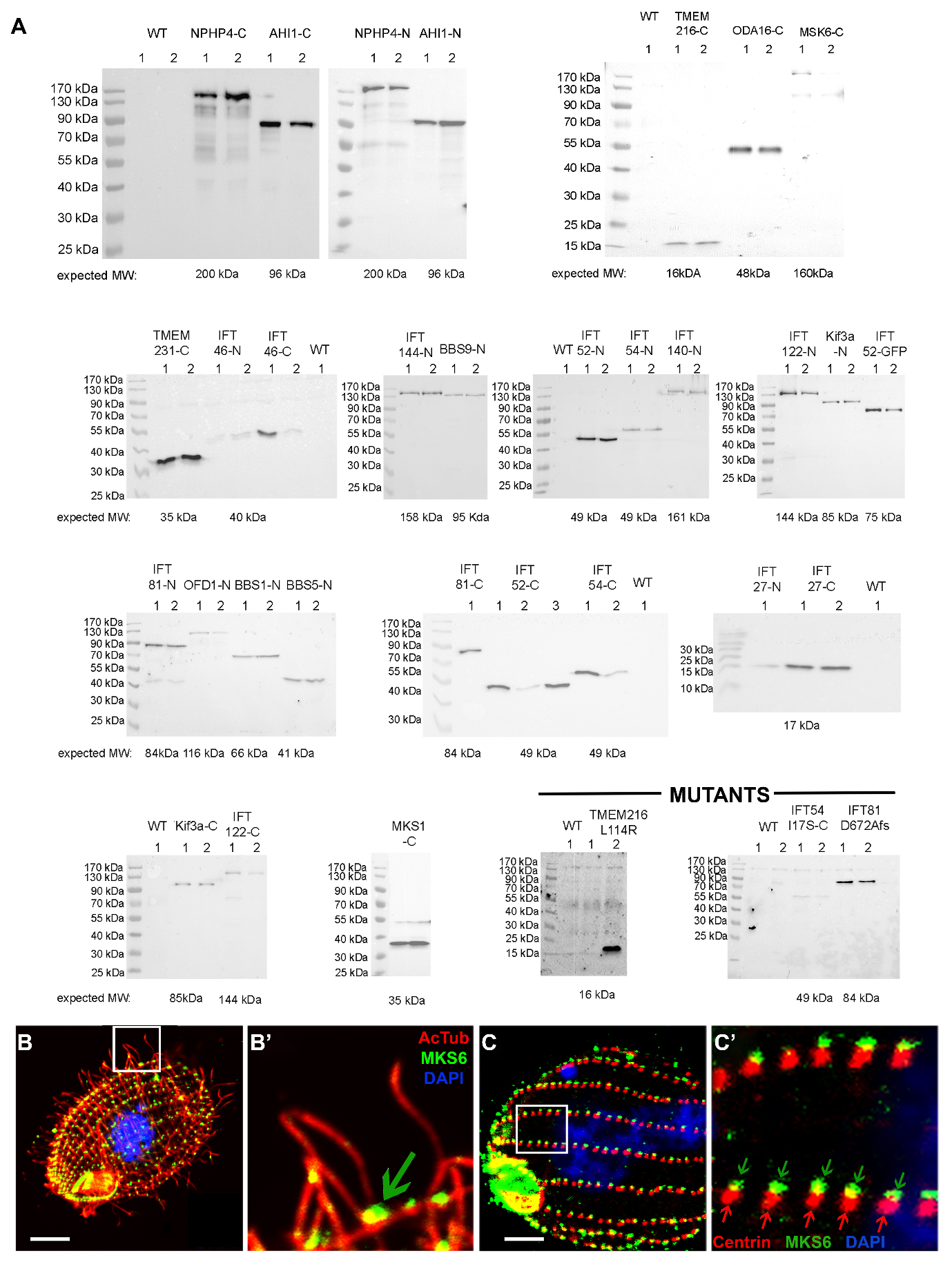
**

**Supplementary** **Figure 2. Localization of HA-tagged TZ proteins in *Tetrahymena thermophila.*** (**A)** Western blotting was performed to validate the molecular weight of fusion proteins and compare them with the theoretical values calculated based on the amino acid sequences, Western blotting was performed on proteins tagged with 3HA at either the C-, N-, or both termini as indicated. **(B-B’)** Confocal images of *Tetrahymena* cell expressing C- terminally tagged MKS6 double labeled with antibody against acetylated α-tubulin to mark cilia (red) and anti-HA antibody to detect MKS6 (green). Arrow shows the localization of MKS6 at the cilium base. (**C-C’**) Confocal images of *Tetrahymena* cell expressing C- terminally tagged MKS6 double labeled with anti-centrin antibody to mark basal bodies (red) and anti-HA antibody to detect MKS6 (green). Cells were also stained with DAPI (blue) to mark the nuclei. Arrows in C’ show localization of MKS6 distal to centrin. **B’** and **C’**, inset of **B** and **C**, respectively.

**
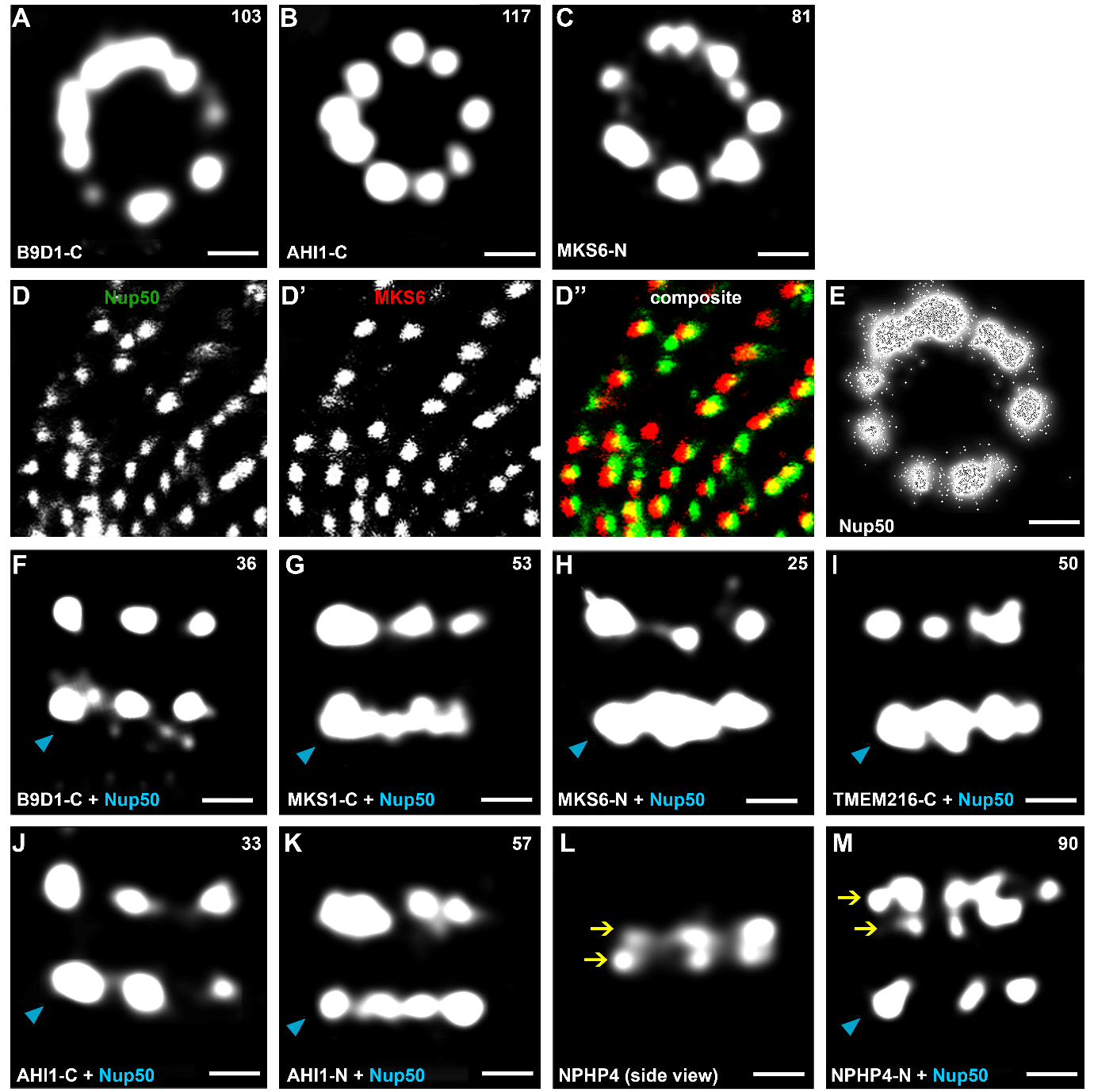
**

**Supplementary** **Figure 3. Radial and axial positions of TZ proteins. (A-C)** STORM images of the top views of C-terminally tagged B9D1 **(A)** and AHI1 **(B)**, and N-terminally tagged MKS6 **(C)**. **(D-D’’)** Confocal images showing the localization of NUP50 relative to the TZ component MKS6 (red) at the cilia base of *Tetrahymena thermophila.* (**E**) STORM image of the top view of NUP50 at the base of a single cilium revealing 9-fold symmetrical rings. **(F-K and M)** Axial positions of TZ proteins relative to NUP50 (indicated by a blue arrowhead). Panel **(L)** shows a side view of the visualization of NPHP4 N-terminus revealing two distinct positions, indicated by yellow arrows. Scale bar in (**A-C,** and **E-M)**, 100 nm. In panels **A-C** and **F-M**, the numbers of rings (or side views) used to calculate the average radius (or axial distance) of each protein are indicated (top right). The rings/side views were collected from at least 10 different cells from at least 3 independent experiments.

**
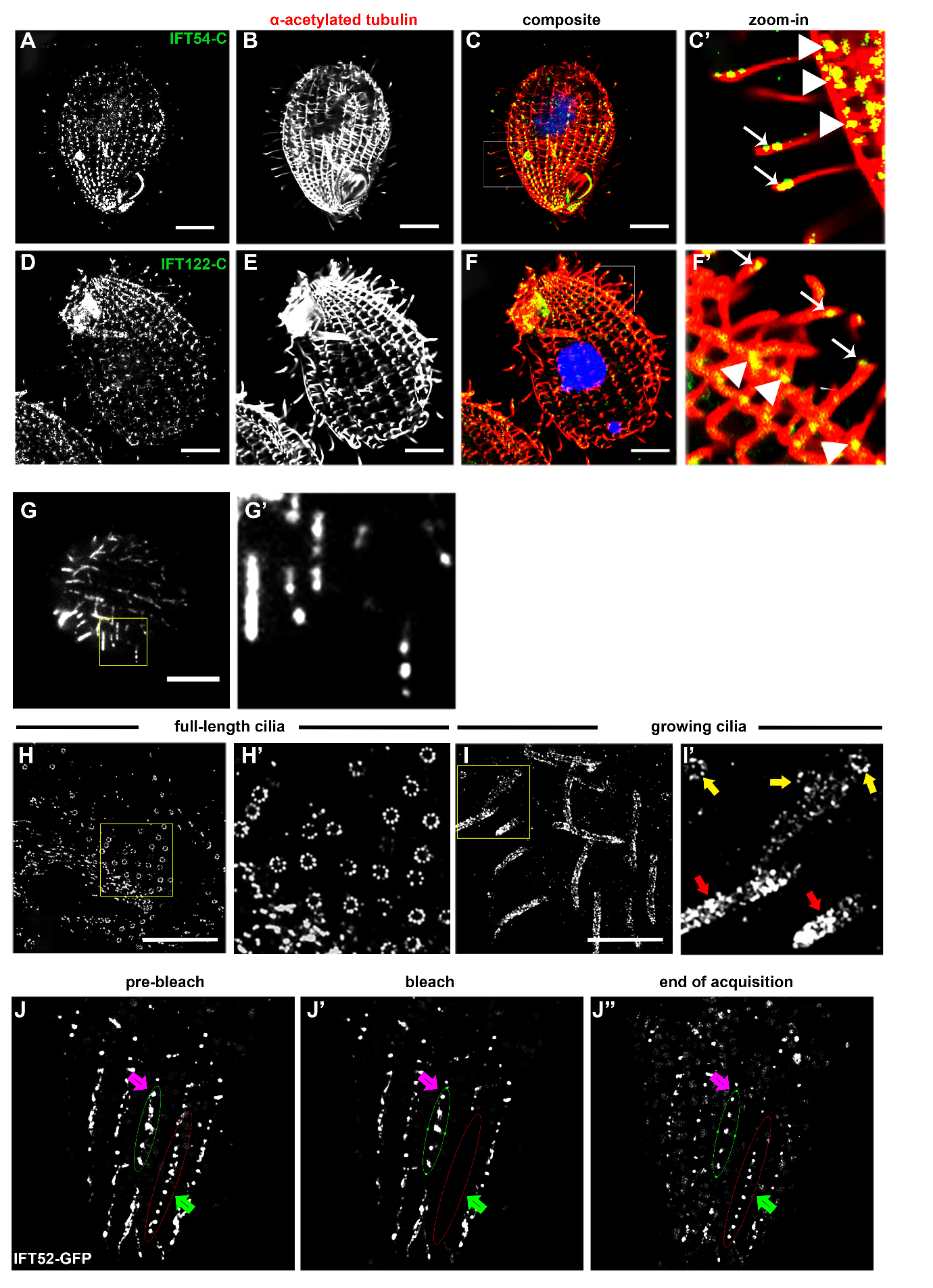
Supplementary** **Figure 4. Localization of IFT proteins in growing versus full-length cilia. (A-F’)** Confocal images of the IFT-B subcomplex component, IFT54 **(A-C’)**, and the IFT-A subcomplex component IFT122 **(D-F’)**, co-stained with anti-acetylated α-tubulin to mark cilia (red). Images show that IFT proteins localize mainly to the base (arrowheads) and tips (arrows) of full-length cilia. **C’** and **F’**, insets in **C** and **F**, respectively. **(G-G’)** TIRF image of IFT54 in regenerated growing cilia after 30 min from deciliation. **G’**, inset in **G**. (**H-I’)** STORM images of IFT54 in full-lengths (**H-H’**) compared to growing cilia (**I-I’**). In I’, yellow arrows, IFT docking sites; red arrows, growing cilia. **(J-J’’)** Still images illustrating fluorescence recovery after photobleaching of IFT54-GFP. Magenta arrows show unbleached region and green arrows show bleached region. Scale bar in **A-C** and **D**-**F**, 5 µm, in **G**, 10 µm and in **H** and **I**, 2 µm.

**
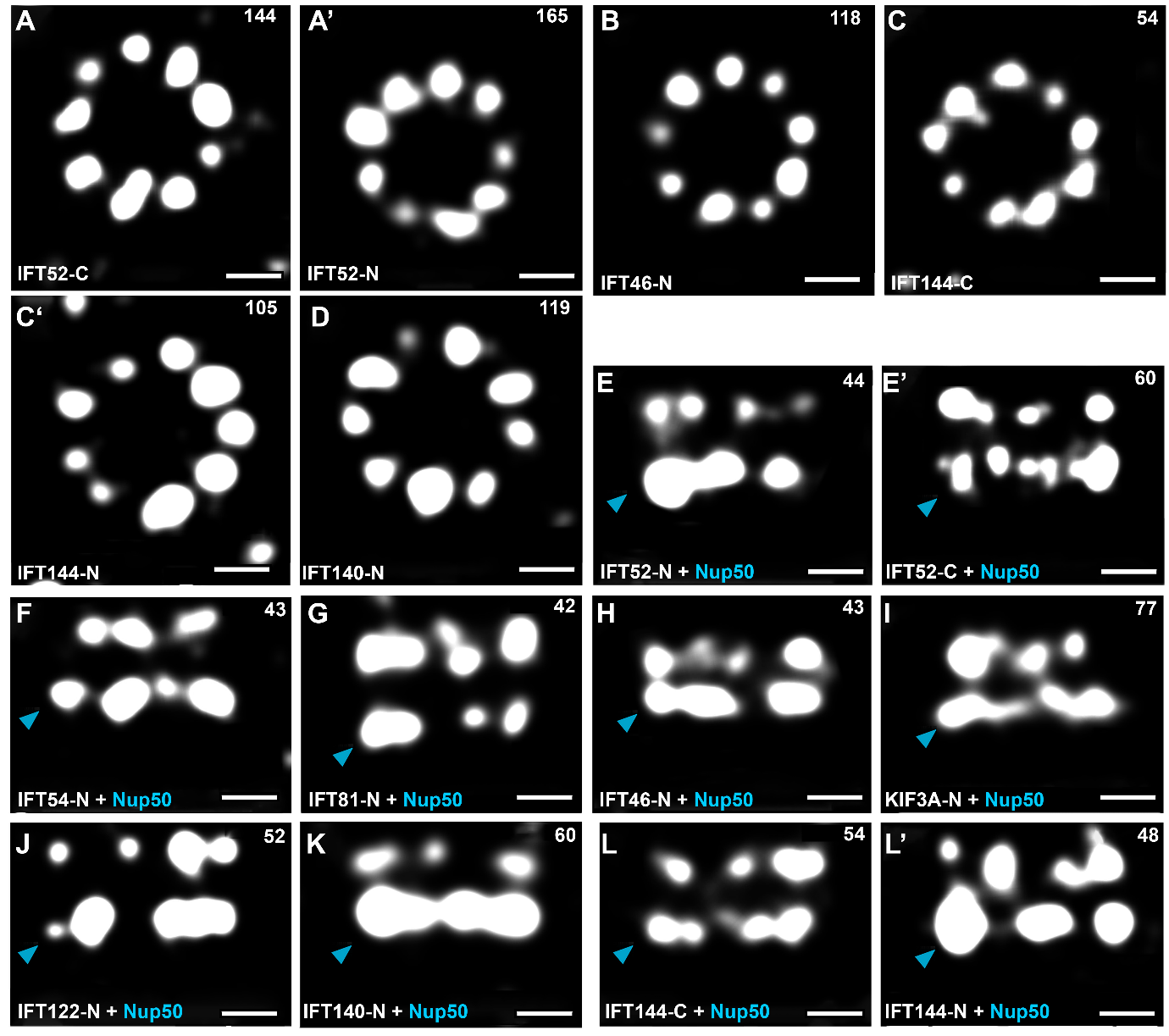
**

**Supplementary** **Figure 5. Radial and axial positions of IFT proteins. (A-E)** STORM images of top views of proteins as indicated. **(F-N’)** STORM images of axial positions of IFT proteins relatively to NUP50 as indicated. Scale bar, 100 nm. In all panels, the numbers of rings (or side views) used to calculate the average radius (or axial distance) of each protein are indicated (top right). The rings/side views were collected from at least 10 different cells from at least 3 independent experiments.

**Supplementary Table 1 – Conservation between *Tetrahymena* and human ciliary proteins investigated in this study**

| **Protein Name** | **Size of protein in humans (aa)** | ***Tetrahymena* protein acc. no.**  **(size in aa)** | **% Positives^*^**  **(% Identities^**^)** |
| --- | --- | --- | --- |
| **AHI1** | 1196 | TTHERM_00460620  (820) | 45 (24) |
| **B9D1** | 204 | TTHERM_00219110  (215) | 62 (39) |
| **MKS1** | 559 | TTHERM_00630490  (492) | 46 (25) |
| **MKS6** | 1620 | TTHERM_00348780  (1599) | 38 (21) |
| **NPHP4** | 1426 | TTHERM_00148790  (1502) | 42 (22) |
| **TMEM216** | 145 | TTHERM_00842510  (149) | 46 (33) |
| **TMEM231** | 316 | TTHERM_000441849  (295) | 36 (23) |
| **IFT27** | 186 | TTHERM_00298510  (148) | 55 (35) |
| **IFT46** | 304 | TTHERM_00193580  (342) | 74 (50) |
| **IFT52** | 437 | TTHERM_00648910  (434) | 59 (42) |
| **IFT54** | 691 | TTHERM_01070330  (451) | 56 (35) |
| **IFT81** | 676 | TTHERM_01013160  (716) | 52 (27) |
| **IFT122** | 1241 | TTHERM_00694540  (1251) | 52 (32) |
| **IFT140** | 1462 | TTHERM_00220810  (1407) | 53 (29) |
| **IFT144** | 1342 | TTHERM_01093530  (1387) | 55 (34) |
| **KIF3A/KIN1** | 699 | TTHERM_00481220  (736) | 57 (41) |
| **ODA16** | 415 | TTHERM_00762930  (426) | 75 (60) |
| **OFD-1** | 1012 | TTHERM_00214750  (976) | (23) |
| **BBS1** | 593 | TTHERM_01084190  (587) | 49 (26) |
| **BBS5** | 341 | [TTHERM_00782070](http://ciliate.org/index.php/feature/details/TTHERM_00782070)  (353) | 54 (34) |
| **BBS9** | 887 | TTHERM_00518740  (829) | 49 (29) |

^* % Positives: The extent to which protein sequences have amino acid residues with similar properties at the same position.^

^** % Identities: The extent to which two protein sequences have the same amino acid residue at the same position.^

**Supplementary Table 2 – URLs of IFT crystal structures**

| **Tetrahymena IFT52-IFT46** | <https://www.ncbi.nlm.nih.gov/Structure/pdb/4UZZ> |
| --- | --- |
| **Chlamydomonas IFT25-IFT27** | <https://www.ncbi.nlm.nih.gov/Structure/pdb/2YC2> |

**Supplementary Table 3 – Conservation of amino acids found mutated in humans**

| **GENE** | **MUTATION** | **SYMPTOMS** | **CONSERVATION** |
| --- | --- | --- | --- |
| IFT81 | Asp672AlafsX15 | RP, Cerebral atrophy | Tetrahymena, hydra, fly, man |
| IFT54 | Ileu17Ser | RP, Cognitive impairment | Tetrahymena, fly, worm, fish, man |
| BBS5 | Ala323CysfsX57 | Bardet-Biedl Syndrome | Tetrahymena, fly, worm, fish, man |
| TMEM216 | L133X | Joubert Syndrome | Tetrahymena, fish, frog, man |
| TMEM216 | L114R | Meckel Gruber Syndrome  (MKS) | Tetrahymena, fish, frog, man |
| TMEM231 | p.Ile232SerfsX | Meckel Gruber Syndrome  (MKS)-Like symptoms | Tetrahymena, fish, mouse, human |

**Supplementary Table 4 - List of primary antibodies and dilutions**

| **Primary antibody** | **Detection** | **Company** | **Catalogue number** | **Dilution** |
| --- | --- | --- | --- | --- |
| Mouse monoclonal  anti-acetylated α-tubulin | Cilia marker | Sigma | T6793S | 1:500 |
| Rabbit monoclonal  anti-HA | HA tag | Cell Signalling | C29F4 | 1:1,600 |
| Mouse anti-HA  conjugated to AF647 | HA tag | Cell signalling | 3444 | 1:100 |
| Mouse anti-HA  conjugated to AF647 | HA tag | Invitrogen | 26183 | 1:150 |
| Rabbit monoclonal  Anti-NUP50 | Basal body? | Abcam | Ab137092 | 1:250 |

**Supplementary Table 5 - List of secondary antibodies and dilutions**

| **Secondary antibody** | **Uses** | **Company** | **Catalogue number** | **Dilution** |
| --- | --- | --- | --- | --- |
| Goat anti-rabbit IgG  (H&L) HRP | WB | Abcam | Ab97051 | 1:10,000 |
| Goat anti-mouse AF488 | IF (confocal) | ThermoFischer | A11029 | 1:1,000 |
| Goat anti-mouse AF568 | IF (confocal) | ThermoFischer | A11004 | 1:1,000 |
| Goat anti-mouse AF647 | IF (confocal and  STORM) | ThermoFischer | A21235 | 1:500 |
| Goat anti-rabbit AF647 | IF (confocal and  STORM) | ThermoFischer | A27040 | 1:500 |

**WB**: Western blot; **IF**: immunofluorescence

**Supplementary Table 6 - List of primer sequences**

| **GENE FRAGMENT (TERMINUS)** | **PRIMER SEQUENCE** |
| --- | --- |
| **TZ** | |
| **AHI1**  **CODING (C)** | 5’ AAAT ACGCGT AAGTTAAGGAGGAGAATTCGAATAA 3’ |
|  | 5’ AATTGGATCCATACGTAGTGTAATAGTTATTTTAGTATTATCTATTG 3’ |
| **AHI1**  **3’UTR (C)** | 5’ AAAT CTGCAG GGTCTAAATCATTTTCCTAGCGTT 3’ |
|  | 5’ AATT CTCGAG TCTATGGGAGTAGTAGAGATTGC 3’ |
| **AHI1**  **CODING (N)** | 5’ AATT ACGCGT T ATG AAT AGC AGA GAT CGT CTT AA 3’ |
|  | 5’ AATT GGATCC GAATTC CTCTAATTAAGCAATCGCGATC 3’ |
| **AHI1 5’UTR (N)** | 5’ TTAA CCGCGG TAAGGAAAGTTTGTTTTAGTGATGA 3’ |
|  | 5’ TTAT CTGCAG AATATTGATGGATAAAGATATAGATAGC 3’ |
| **B9D-1**  **CODING (C)** | 5’ AAAT ACGCGT CTA GCA TGC ATG ATC AGT CAA T 3’ |
|  | 5’ AATT GGATCC TGAGTAACCTTCTCTTTTAAAATTTCTC 3’ |
| **B9D-1**  **3’UTR (C)** | 5’ AAAT CTGCAG GCA AAC AAT CGC TTT AGT CTT TTA G 3’ |
|  | 5’ AATT CTC GAG AAGCTT TGAGCAAATAAAGGTGG 3’ |
| **MKS1**  **CODING (C)** | 5’ AAAT ACGCGT CTCATAACTAACTTCTGCTTCCC 3’ |
|  | 5’ AATT GGATCC ATTTAATTTCTACGTAGTATATCTTAGTGAAG 3’ |
| **MKS1**  **3’UTR (C)** | 5’ AAAT CTGCAG CAACTAATTAACTAACTAACTAACTTATTCTAT 3’ |
|  | 5’ AATT CTCGAG TTGATATCTGCTATTTTCCAACAGTT 3’ |
| **MKS6**  **CODING (C)** | 5’ AATT ACGCGTGGTCATGCTATACCTGAAGG 3’ |
|  | 5’ AATT GGATCCATCTTCGTATATTACACCTACAAATACC 3’ |
| **MKS6**  **3’UTR (C)** | 5’ TTAA CTGCAGAAAAGCTTAGATATGAAACAATTTAATTATCA 3’ |
|  | 5’ TTAA CTCGAGAATATAACTCTCATTAGCAGCCA 3’ |
| **NPHP4 CODING (C)** | 5’ AAAT ACGCGT CCT CAT AGA TAG GTT GTT GAT CAC 3’ |
|  | 5’ AATT GGATCC TTTATTGACTGTAAAATGCAATTTAAATAGAA 3’ |
| **NPHP4**  **3’UTR (C)** | 5’ AAAT CTGCAG ATG AAG AGG GAG TTT GTT GG 3’ |
|  | 5’ AATT CTCGAG GAGGTGAAAGCTTCTGTTTGCATTAG 3’ |
| **TMEM216 CODING (C)** | 5’ AAAT ACGCGT T ATG TCA AGC TCA TAG AAA ATTAGAGATAG 3’ |
|  | 5’ AATT GGATCC GTATTATTGCTTTTCAAAGTTTTTAAAAC 3’ |
| **TMEM216**  **3’UTR (C)** | 5’ AAAT CTGCAG TTCTTGTGCTGGTTAAACTTAGAATA 3’ |
|  | 5’ AATT CTCGAG TCCTCTTCTACTTCTTCCTACA 3’ |
| **TMEM231 CODING (C)** | 5’ AAAT ACGCGT GGA AGG ATA AGA AAA TAG GGT AGC 3’ |
|  | 5’ AATT GGATCC ATCTTATTTATATTTTCCGAACTATCTAAATGC 3’ |
| **TMEM231**  **3’UTR (C)** | 5’ AAAT CTGCAG GTGGTATGCATTCAATCAATTAATCAG 3’ |
|  | 5’ AATT CTCGAG ATTGAACTACTCTCCCTCAATTTAG 3’ |
| **IFT** | |
| **IFT27**  **CODING (C)** | 5’ AATA ACGCGT AG AAGTTT CTTCCA AAATCA TTCC 3’ |
|  | 5’ AATT GG ATCC TA CAAGTC TTTCTG CTAAGT TTTAGA 3’ |
| **IFT27**  **3’UTR (C)** | 5’ AAAT CTGCAG CT CTCAAA ATATTT TAAATC AATTGT TGC 3’ |
|  | 5’ AATT CTCGAG CA AACTTT AGTGAT GATCCA AAGT 3’ |
| **IFT27**  **CODING (N)** | 5’ AATTGG ATCCAG AGCATA CACATC ATTGAA GTG 3’ |
|  | 5’ AATT ACGCGT TATG AAGA AAATAA AAGCAA AAGTTT TAGTTG 3’ |
| **IFT27**  **5’UTR (N)** | 5’ TTAACT GCAGAA ATGCGA TTTAAT GATTGA TTTCAA AC 3’ |
|  | 5’ TTAACC GCGGCA GACTCT GATTAT CTTCCA G 3’ |
| **IFT46 CODING (C)** | 5’ AATA ACGCGT AGG TGC TTA TAA TCC AGC TG 3’ |
|  | 5’ AATTGGATCCATTAATTTGGAGCTGCATCTAATC 3’ |
| **IFT46 3’UTR (C)** | 5’ AAAT CTGCAG CACTGTATCAGCTATAATCAGAAAG 3’ |
|  | 5’ AATTCTCGAGCATCATCTTCCTCTTCACTTTC 3’ |
| **IFT46 CODING (N)** | 5’ AATT ACGCGT T ATG TCA GAT TCA GAC GAT AGG G 3’ |
|  | 5’ AATTGGATCCGGCTTAAGCTTGGCATCTAG 3’ |
| **IFT46**  **5’UTR (N)** | 5’ TTAA CCGCGG TC ACA CTA TTA AGC TTA TTA ATG TTT AG 3’ |
|  | 5’ TTAACTGCAGATTTTGATTTTTGCAACTATTTAGTTTATC 3’ |
| **IFT52 CODING (C)** | 5’ AAAT ACGCGT GGAAAGGATGATGAAGAAGATGAAT 3’ |
|  | 5’ AATT GGATCC GTTATTGAGCTTCTTGAAATTTATCTGT 3’ |
| **IFT52 3’UTR (C)** | 5’ AAAT CTGCAG AACAAATCATGCTTCCATTCAA 3’ |
|  | 5’ AATT CTC GAG AAGCTT CTAATCCTACATGTTATGTCT 3’ |
| **IFT52 CODING (N)** | 5’ AATT ACGCGT T ATG TCT GGT GAG TAA AAG ATA ATT GTC TTC  AAC GCT TCA AAG AAGG 3’ |
|  | 5’ AATT GGATCC GTACGAGGCGTTGCTTAAAATA 3’ |
| **IFT52 5’UTR (N)** | 5’ TTAA CCGCGG GATATC ATGCAATTCTATATTTGAGGTGAG 3’ |
|  | 5’ TTAA TCGGCTAAATGA CTGCAGT 3’ |
| **IFT54 CODING (C)** | 5’ AAAT ACGCGT GCT ACA ACT CCT GAA TTA TGC T 3’ |
|  | 5’ AATT GGATCC TTTGGTTGAAACAACAGAGTAGAGC 3’ |
| **IFT54**  **3’UTR (C)** | 5’ AAAT CTGCAG CATTTGTCTATCTAGCTAATTACAAACGT 3’ |
|  | 5’ AATT CTCGAG GCACCTTAGAAAGCTTAATTATGC 3’ |
| **IFT54 CODING (N)** | 5’ AATT ACGCGT T ATGGCTGATGATAAATTTTGGGA 3’ |
|  | 5’ AATT GGATCC GTGGTTCATGCTTTCGATATC 3’ |
| **IFT54 5’UTR (N)** | 5’ TTAA CCGCGG GAATTC CAATCAAGAAGGAGTTGCACTT 3’ |
|  | 5’ TTAA CTGCAG CGCTTTAAACGTCTCTTTTGC 3’ |
| **IFT81 CODING (C)** | 5’ AAAT ACGCGT TCT TGC TTT CAC GTA CTG AAG 3’ |
|  | 5’ AATT GGATCCATTAATAGTTAATATATTATAGTCTCCACCAG 3’ |
| **IFT81 3’UTR (C)** | 5’ AAAT CTGCAG AATCATAGAATATTTGCATATTTGTTAAAAGC3’ |
|  | 5’ AATT CTCGAGCCAATCATTCATTTTCAGACGTG 3’ |
| **IFT81**  **CODING (N)** | 5’ TTAA CCGCGG CGATCTCACTACTATTCTTGAGTGT 3’ |
|  | 5’ TTAA CTGCAG GTATTTGACTATGTTAGCGATTATGC 3’ |
| **IFT81**  **5’UTR (N)** | 5’ AATT ACGCGT T ATG AATTATAATAGCTACGGAAATCCG 3’ |
|  | 5’ CTG CTT TGC GCG AAT CTT A GGATCC AATT 3’ |
| **IFT122 CODING (C)** | 5’ AAAT ACGCGT ACTTACACTGACAATTCAAAATTAATT 3’ |
|  | 5’ AATT GGATCC GAAATCAAAAACATCTTTCTAAGGTCCT 3’ |
| **IFT122 3’UTR (C)** | 5’ AAAT CTGCAG TTCTACTTAAAGACTACCCATAAACT 3’ |
|  | 5’ AATT CTCGAG GGATTAATTCTGGTGATAGGTAAG 3’ |
| **IFT122 CODING (N)** | 5’ AATT ACGCGT C ATGAAGTGTTCAGAACTATGGAC 3’ |
|  | 5’ AATT GGATCC TATTCGAGAGCTCTGTTTCTTATT 3’ |
| **IFT122 5’UTR (N)** | 5’ TTTT CCGCGGGATATCATAATGCAAATAATGGTGGTTGATAC 3’ |
|  | 5’ TTAA CTGCAG CCTAAAATTTGCTTTCTATTCTGC 3’ |
| **IFT140 CODING (N)** | 5’ AATT ACGCGT T ATG AGC TTG TTT TCA GAA CTT CC 3’ |
|  | 5’ AATT GGATCC TGCTTGGGCCTGTCTTA 3’ |
| **IFT140 5’UTR (N)** | 5’ TTAA CCGCGG GATTTAACTATAAATGATTGTTCCTCAAC 3’ |
|  | 5’ TTAA CTGCAG TGCTTGAAGTCTCTCTTTCTCT 3’ |
| **IFT144 CODING (C)** | 5’ AAAT ACGCGT CAG ACC ACT AGC TTA CAA GTG 3’ |
|  | 5’ AATTGGATCC CTTCTTCTTTTACTCAGAACTTTATCTTGTTGCTC 3’ |
| **IFT144 3’UTR (C)** | 5’ AAAT CTGCAG GTTAGATTCATGCATTAATTATTAGTTCAA AG 3’ |
|  | 5’ AATT CTCGAGCTTGAAGACTCAGATCTGGAAATAG 3’ |
| **IFT144 CODING (N)** | 5’ AATT ACGCGT T ATG GCA TCA AGC AAG AAA GTA ATA C 3’ |
|  | 5’ AATT GGATCC TTAACAATCCTTCATCGTTCCAAT 3’ |
| **IFT144**  **5’UTR (N)** | 5’ TTAA CCGCGG ATGTGTAAGCTTGTGTAATGAAC 3’ |
|  | 5’ TTAA CTGCAG GCAGGATATTCAGAAATCAATTATATTTTTT 3’ |
| **KIF3a**  **CODING (C)** | 5’ AATTGG ATCCCT TCTTAG GTTATA ATCCTT TTGCTT TGGG 3’ |
|  | 5’ AAATAC GCGTAT ATGCCC ACAGAG CTAAAT C 3’ |
| **KIF3a**  **3’UTR (C)** | 5’ AATTCT CGAGGC TTGGTG TGTAAT TGCT 3’ |
|  | 5’ AATTCT GCAGAT TAACAA ATTTGA GTAAAA AATATG CTATG 3’ |
| **KIF3a**  **CODING (N)** | 5’ AATTGG ATCCAA CGTTAG CAACCA TAACA 3’ |
|  | 5’ AATTAC GCGTTA TGAGCA AGTAAG AAAAGA ATAGTA AAAATG ATG 3’ |
| **KIF3a**  **5’UTR (N)** | 5’ TTAACT GCAGAA GCTTCT ATTATC TTTTGT CAAATT ACTCCC 3’ |
|  | 5’ TTAACC GCGGAT CTTCTG ATACCT GAATAA GCAAG 3’ |
| **BBSome** | |
| **BBS1**  **CODING (N)** | 5’ AATT AG A TCT CC TGAGTT AACTTT AAAGCT AAACTG 3’ |
|  | 5’ AATTAC GCGTTA TGTCTA AGTCTA AGATAT TACTTA CTGATA AATC 3’ |
| **BBS1**  **5’UTR (N)** | 5’ TTAACT GCAGAA TCTCAA ATTAAT TTGCTT CATTCT TG 3’ |
|  | 5’ TTAACC GCGGGA TATCCA TTTGTA AATGAC GTTATC ATCAG 3’ |
| **BBS5**  **CODING (N)** | 5’ AATTAC GCGTTA TGGAAT ACGAGT ACTTCT ATGACA GGGAGA TAA 3’ |
|  | 5’ TTAACT GCAGTG AAGAGC CTTTTC AAAATA TTAAAA GA 3’ |
| **BBS5**  **5’ UTR (N)** | 5’ TTAACC GCGGAA TTCTCC CGTACC CGTACT TT 3’ |
|  | 5’ AATT AG A TCT CC TGAGTT AACTTT AAAGCT AAACTG 3’ |
| **BBS9**  **CODING (C)** | 5’ AATTGG ATCCTT ACTTTT CTAGTT AGCCTT TTGAGA TTTTG 3’ |
|  | 5’ AAATAC GCGTGC ACCTAA TACTTA TACCTT TGAATA 3’ |
| **BBS9**  **3’ UTR (C)** | 5’ AATTCT CGAGAA GCTTCC ATATTA GTACCT AGCAGA ATTTG 3’ |
|  | 5’ AAATCT GCAGTA TTTTTA ATGAAG GAGCTA TTTTAA AATG 3’ |
| **BBS9**  **CODING (N)** | 5’ AATTGG ATCCAA ACACAC TCCAGC TTGA |
|  | 5’ AATTAC GCGTTA TGTCAT TATTTC AAACAA AAGAAT GG 3’ |
| **BBS9**  **5’ UTR (N)** | 5’ TTAACT GCAGCC TTAAAA AACAGT CAGTCT AAACTT TC 3’ |
|  | 5’ TTAACC GCGGAA TTCGAT TAATTA ATTTGA TGTACT AATCAG CA 3’ |
| **OTHERS** | |
| **OFD1**  **CODING (N)** | 5’ AATT ACGCGT T ATG GAA TAA GGA GAA GAT AAT AAT GAT T 3’ |
|  | 5’ AATT GGATCC AACCTTCACTTGCTTTATCTTG 3’ |
| **OFD1**  **5’UTR (N)** | 5’ TTAA CCGCGG AATT CCCTTTATATTTAACAAGATATTCACC 3’ |
|  | 5’ TTAA CTGCAG CCTACATTCATTAACTCATTCATATAGC 3’ |
| **OFD1 CODING (C)** | 5’ AAAT ACGCGT CTCTAGGCTAAGAGTTATGAGTGAAG 3’ |
|  | 5’ AATT GGATCCGAAATATTCTAGATCTTCAACGATTTC 5’ |
| **OFD1 3’UTR (C)** | 5’ AAAT CTGCAG AAATCTAAATATAATTAATCAGTCTACTAT 3’ |
|  | 5’ AATTCTCGAGAAGCTTGAATAACTTGCTCAGAAATGCCC 3’ |
| **ODA16 CODING (C)** | 5’ AAAT ACGCGT GAT AAG GTA GTA ACA GGT TCA TTT G 3’ |
|  | 5’ AATT GGATCC ATTTGTAGGAGGTTTATGTAATAACTC 3’ |
| **ODA16**  **3’UTR (C)** | 5’ AAAT CTGCAG GCG ATA TCT AAA CAA AGA CCT AAA TAG 3’ |
|  | 5’ AATT CTCGAG AAGCTTAACATTGAATTGTTGGTTGCT 3’ |

**Literature cited**

1. Dave, D., D. Wloga, and J. Gaertig, *Manipulating ciliary protein-encoding genes in Tetrahymena thermophila.* Methods Cell Biol, 2009. **93**: p. 1-20.

2. Urbanska, P., et al., *The CSC proteins FAP61 and FAP251 build the basal substructures of radial spoke 3 in cilia.* Mol Biol Cell, 2015. **26**(8): p. 1463-75.

3. Urbanska, P., et al., *Ciliary proteins Fap43 and Fap44 interact with each other and are essential for proper cilia and flagella beating.* Cell Mol Life Sci, 2018. **75**(24): p. 4479-4493.

4. Gaertig, J., et al., *High frequency vector-mediated transformation and gene replacement in Tetrahymena.* Nucleic Acids Res, 1994. **22**(24): p. 5391-8.

5. Shang, Y., et al., *A robust inducible-repressible promoter greatly facilitates gene knockouts, conditional expression, and overexpression of homologous and heterologous genes in Tetrahymena thermophila.* PNAS, 2002. **99**(6): p. 3734-3739.

6. Janke, C., et al., *Tubulin polyglutamylase enzymes are members of the TTL domain protein family.* Science, 2005. **308**(5729): p. 1758-62.

7. Gaertig, J., et al., *Discovery and functional evaluation of ciliary proteins in Tetrahymena thermophila.* Methods Enzymol, 2013. **525**: p. 265-84.
